# Revision of the *Merodon serrulatus* group (Diptera, Syrphidae)

**DOI:** 10.3897/zookeys.909.46838

**Published:** 2020-02-05

**Authors:** Ante Vujić, Laura Likov, Snežana Radenković, Nataša Kočiš Tubić, Mihajla Djan, Anja Šebić, Celeste Pérez-Bañón, Anatolij Barkalov, Rüstem Hayat, Santos Rojo, Andrijana Andrić, Gunilla Ståhls

**Affiliations:** 1 University of Novi Sad, Department of Biology and Ecology, Trg Dositeja Obradovića 2, Novi Sad, Serbia; 2 Department of Environmental Sciences and Natural Resources, Faculty of Sciences III, Campus of San Vicente, University of Alicante, Spain; 3 Institute of Systematics and Ecology of Animals, Russian Academy of Sciences, Siberian Branch, Novosibirsk, Russia; 4 Department of Plant Protection, Faculty of Agriculture, Akdeniz University, Antalya, Turkey; 5 University of Novi Sad, BioSense Institute, Dr Zorana Đinđića 1, Novi Sad, Serbia; 6 Zoology Unit, Finnish Museum of Natural History Luomus, University of Helsinki, Finland

**Keywords:** 28S rRNA, COI, immature stages, lectotype, morphology, new species, new synonyms, taxonomy

## Abstract

The phytophagous hoverfly genus *Merodon* Meigen, 1803 (Diptera, Syrphidae), which comprises more than 160 species distributed in Palaearctic and Afrotropical regions, can be differentiated into multiple groups of species that harbor high levels of hidden diversity. In this work, the *serrulatus* species group of *Merodon* is revised, providing an illustrated key to species, a detailed discussion on the taxonomic characters and a morphological diagnosis, including also the first data about the preimaginal morphology of this species group. The study includes characteristics of the 13 species of the *M. serrulatus* group, along with the available distributional data. Moreover, descriptions are provided for seven new species, namely *M. defectus* Vujić, Likov & Radenković **sp. nov.**, *M. disjunctus* Vujić, Likov & Radenković **sp. nov.**, *M. medium* Vujić, Likov & Radenković **sp. nov.**, *M. nigrocapillatus* Vujić, Likov & Radenković **sp. nov.**, *M. nigropunctum* Vujić, Likov & Radenković **sp. nov.**, *M. opacus* Vujić, Likov & Radenković **sp. nov.**, and *M. trianguloculus* Vujić, Likov & Radenković **sp. nov.** In addition, the taxa *M. serrulatus* (Wiedemann in Meigen, 1822), *M. bequaerti* Hurkmans, 1993, *M. hirsutus* Sack, 1913, *M. kawamurae* Matsumura, 1916, *M. sacki* (Paramonov, 1936) and *M. sophron* Hurkmans, 1993 are redefined and redescribed. Following a detailed study of the type material sourced from different entomological collections, the status of all available taxa related to *M. serrulatus* is revised and a new synonymy is proposed: *M. tener* Sack, 1913 **syn. nov.** (junior synonym of *M. serrulatus*). The identity of *M. trizonus* (Szilády, 1940) could not be assessed as the type specimens are lost. Thus, the name *M. trizonus* is considered as *nomen dubium*. The monophyly and composition of this species group are assessed through Maximum Parsimony and Maximum Likelihood analyses of the mitochondrial COI and nuclear 28S rRNA gene sequences.

## Introduction

The phytophagous hoverfly genus *Merodon* Meigen, 1803 contains more than 160 species distributed across the Palaearctic and Afrotropical regions (Ståhls et al. 2009). Adults mimic bees and bumblebees (Hymenoptera: Apidae) and feed on pollen and nectar from early spring to autumn (Hurkmans 1993; Speight 2018). Based on the immature stages of *Merodon* found to date, underground storage organs (bulbs, corms and rhizomes) of geophytes of the families Asparagaceae, Amaryllidaceae and Iridaceae are larval microhabitats of this taxon and phytophagy is its feeding mode (Ricarte et al. 2017; Preradović et al. 2018). The immature stages of only eight *Merodon* species have been described to date (Heiss 1938; Stuckenberg 1956; Ricarte et al. 2008, 2017; Andrić et al. 2014; Preradović et al. 2018) and a detailed literature review on the immature stages of *Merodon*, including host plants, has recently been published by Ricarte et al. (2017).

The taxonomic status and identification of many *Merodon* species requires further investigation, as the genus contains a high number of species groups consisting of morphologically cryptic taxa with very subtle morphological differences. In various recent publications, an integrative taxonomic approach combining morphological and molecular information has been adopted and resulted useful in resolving taxonomic ambiguities in hoverflies, e.g., in *Merodon equestris* species complex (Marcos-García et al. 2011), *Merodon avidus* complex (Popović et al. 2015; Ačanski et al. 2016), *Merodon aureus* species group (Šašić et al. 2016), genus *Chrysotoxum* Meigen, 1803 (Nedeljković et al. 2013, 2015) and *Melanostoma* Schiner, 1860 (Haarto and Ståhls 2014).

Most recent publications pertaining to the genus *Merodon* have focused on particular species groups, within which the authors delimited individual species (Vujić et al. 2012, 2013, 2015; Ačanski et al. 2016; Šašić et al. 2016; Veselić et al. 2017; Kočiš Tubić et al. 2018; Radenković et al. 2018a). The *Merodon avidus-nigritarsis* lineage was confirmed as one of four main lineages in the genus *Merodon*, alongside with three other lineages: *albifrons+desuturinus*, *aureus* and *natans*. Likov et al. (2019) presented a phylogenetic inference where the *Merodon avidus-nigritarsis* lineage was resolved in a similar way as in the studies by Šašić et al. (2016) and Radenković et al. (2018b). In the same study, Likov et al. (2019) divided the *M. avidus-nigritarsis* lineage into 10 species groups (namely *M. aberrans*, *M. aurifer*, *M. avidus*, *M. clavipes*, *M. fulcratus*, *M. italicus*, *M. nigritarsis*, *M. pruni*, *M. serrulatus*, and *M. tarsatus* groups), and five species were not included in any of these species groups (i.e., *M. clunipes* Sack, 1913, *M. crassifemoris* Paramonov, 1925, *M. eumerusi* Vujić, Radenković & Likov, 2019, *M. murinus* Sack, 1913, and *M. ottomanus* Hurkmans, 1993).

**Figure 1.**
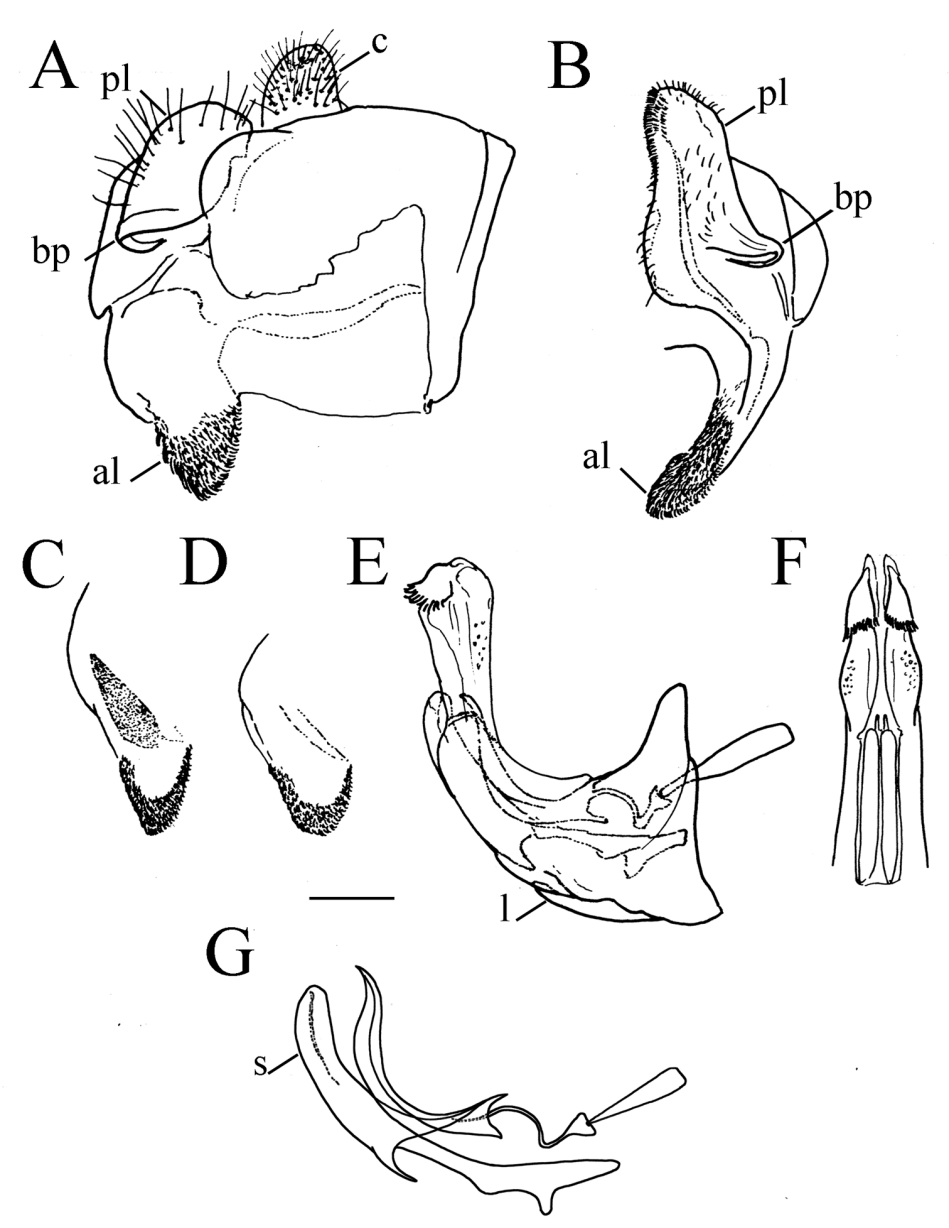
*Merodon serrulatus* male genitalia. **A** Epandrium, lateral view **B** Epandrium, ventral view **C, D** Posterior surstyle lobe, lateral view **E** Hypandrium, lateral view **F** Part of hypandrium, ventral view **G** Aedeagus, lateral view. Abbreviations: al–anterior surstyle lobe, bp–basolateral protrusion, c–cercus, l–lingula, pl–posterior surstyle lobe, s–lateral sclerite of aedeagus. Scale bar: 0.2 mm.

The *Merodon serrulatus* species group includes taxa with a characteristic basolateral protrusion on the posterior surstyle lobe (Fig. 1). Based on recently published data, this group contains six already described species, i.e., *Merodon bequaerti* Hurkmans, 1993, *M. hirsutus* Sack, 1913, *M. kawamurae* Matsumura, 1916, *M. sacki* (Paramonov, 1936), and *M. serrulatus* (Wiedemann in Meigen, 1822) (Likov et al. 2019).

In this study, we present a taxonomic review of the *serrulatus* species group based on a detailed examination of material gathered as a part of our long-term field research in the Palaearctic region, especially in the Mediterranean and the Middle East. Our aims are 1. to review materials stored in several major entomological institutions and private collections holding specimens of this group; 2. to define and describe the taxa within the *serrulatus* species group, including new species; 3. to infer the phylogenetic relationships among the members of this species group using mtDNA COI gene and the 28S rRNA gene; and 4. to present the first data about the preimaginal morphology of the *M. serrulatus* species group.

## Materials and methods

### Material examined

Most of the recently collected specimens were sampled by sweep net. Further specimens of the *Merodon serrulatus* species group were sourced from collections deposited in museums and universities which are listed below. Consisted total of 1,083 specimens collected from 1837 to 2018 across 22 countries (i.e., Algeria, China, Croatia, France, Greece, Israel, Italy, Kazakhstan, Kyrgyzstan, Libya, Montenegro, Morocco, North Macedonia, Portugal, Russia, Spain, Syria, Tajikistan, Tunisia, Turkey, Turkmenistan, and Uzbekistan) were studied for the present study.

The information on labels of the material examined is provided for each studied specimen in the following order: country name, a bullet point (indicating the beginning of a material citation), number and sex of specimen(s), locality data, geographical coordinates, altitude, collection date, collector(s) followed by “leg.”, institutional acronym and specimen codes/unique identifiers (“to” indicates range). The specimens are listed alphabetically by country and subsequently by increasing latitude (south to north) within each country. In the quotations of the type specimens’ original label data, double quotation marks were used to indicate separate labels, and the slash was adopted to indicate a new line within a label, with additional details and interpretations provided in square brackets, where applicable.

### Institutional acronyms

**A. S. coll.** Axel Ssymank collection, Achtberg, Germany (ssymanka@t-online.de)

**CEUA** Colección Entomológica de la Universidad de Alicante, Alicante, Spain

**D. D. coll.** Dieter Doczkal collection, Munich, Germany (dieter.doczkal@gmail.com)

**EMIT** Entomological Museum of Isparta, Isparta, Turkey

**FSUNS** Faculty of Sciences, Department of Biology and Ecology, University of Novi Sad, Novi Sad, Serbia

**G. V. W. coll.** Guy Van de Weyer collection, Reet (Rumst), Belgium (guido.vandeweyer@skynet.be)

**GLAHM** Hunterian Zoology Museum, University of Glasgow, Glasgow, UK

**J. T. S. coll.** John T. Smit collection, Utrecht, the Netherlands (John.Smit@naturalis.nl)

**J. v. S. coll.** Jeroen van Steenis collection, Amersfoort, the Netherlands (jvansteenis1@gmail.com)

**M. B. coll.** Miroslav Barták collection, Prague, Czech Republic (bartak@af.czu.cz)

**M. H. coll.** Martin Hauser collection, Sacramento, USA (martin.hauser@cdfa.ca.gov)

**MAegean** The Melissotheque of the Aegean, University of the Aegean, Mytilene, Greece

**MNHN** Muséum National d’Histoire Naturelle, Paris, France

**MZH** Finnish Museum of Natural History, University of Helsinki, Helsinki, Finland

**NBCN** Naturalis Biodiversity Center [formerly known as the National Museum of Natural History (RMNH)], Leiden, The Netherlands

**NHMUK** Natural History Museum, London, UK

**NHMW** Museum of Natural History (Naturhistorisches Museum Wien), Vienna, Austria

**NMNH** The Department of Entomology, of the National Museum of Natural History, Smithsonian Institution, Washington, DC, USA

**NMPC** National History Museum, Prague, Czech Republic

**NMS** National Museums Scotland, Edinburgh, UK

**S. K. coll.** Sakari Kerppola collection, Helsinki, Finland (sakari.kerppola@helsinkinet.fi)

**S. S. coll.** Süleyman Sarıbıyık collection, Kastamonu, Turkey

**SIZK** I.I. Schmalhausen Institute of Zoology of National Academy of Sciences of Ukraine, Kiev, Ukraine

**SZMN** Siberian Zoological Museum of the Institute of Systematics and Ecology of Animals, Siberian Branch of the Russian Academy of Sciences, Novosibirsk, Russia

**TAUI** Tel Aviv University, Tel Aviv, Israel

**WML** World Museum Liverpool, Liverpool, UK

**ZHMB** Zoological (Zoologisches) Museum of the Humboldt University, Berlin, Germany

**ZMKU** Zoological Museum, State University of Kiev, Kiev, Ukraine

**ZMUC** Zoological Museum, Natural History Museum of Denmark, University of Copenhagen, Copenhagen, Denmark

### Taxonomic study of adults

The type material of all described species of the *Merodon serrulatus* species group were studied, with the exception of the type material of *Merodon trizonus* (Szilády, 1940) because the type specimens are lost.

To study the male genitalia, dry specimens were relaxed in a closed pot containing water to ensure high humidity levels, and the genitalia were extracted using an insect pin with a hooked tip. Genitalia were cleared by boiling them individually in tubes of water-diluted KOH pellets for 3–5 minutes. This was followed by brief immersion in acetic acid to neutralize the KOH, immersion in ethanol to remove the acid, and storage in microvials containing glycerol. Specimens’ measurements were taken in dorsal view with a micrometer and are presented as ranges. Body length was measured from the lunule to the end of the abdomen. Drawings were made using a FSA 25 PE drawing tube attached to a binocular microscope Leica MZ16. Specimens photographs were captured by a Nikon D7100 camera connected to a personal computer, as well as a Leica DFC 320 digital camera attached to a Leica MZ16 binocular microscope. After photographing, CombineZ software (Hadley 2006) was used in order to create composite image with an extended depth of field, created from the in-focus areas of each image.

Terminology adopted in the morphological descriptions follows Thompson (1999) and, for male genitalia, Marcos-García et al. (2007), while the term “fossette” follows Doczkal and Pape (2009).

Localities were geo-referenced in Google Earth (Google Inc, California, USA, https://www.google.com/earth; accessed on 10.02.2019). Geographic coordinates of localities were represented in GenGIS (v 2.5.3) (Parks et al. 2013) in order to create distributional maps.

### The study of preimaginal morphology

Sampling

A targeted search for immature *Merodon* hoverflies was conducted in the chestnut forest of Agiassos, Lesvos Island (Greece). Abundant population of *M. serrulatus* and other *Merodon* species were found at this locality. Searches for larvae were carried out during a field trip from February 27 to March 10 2006, as owing to their biological cycle, these *Merodon* species would be in immature stages (larvae or pupae) during this period. An area of ca. 3m^2^, where the presence of many bulb species and adults of *Merodon* were reported the previous year was selected. The whole area was excavated to a depth of approximately 20 cm and the soil was sieved searching for the larvae. Only one larva (third larval stage) was found in the soil surrounding bulbs of different plant genera, such as *Fritillaria* Tourn. ex L., *Gagea* Salisb., *Muscari* Miller, and *Ornithogalum* L. This solitary larva was kept in a plastic container with the soil in which it had been found at room temperature until it pupated two days later. The adult of *M. opacus* sp. nov. emerged on 21 March 2006 after spending 17 days in the pupal stage.

Morphological study

The cephalopharyngeal skeleton was removed from the antero-ventral margin of the puparium using entomological pins. After dissection, the cephalopharyngeal skeleton was soaked in 10% KOH and heated for 15 min in order to remove the remaining tissues attached, after which it was soaked for a few minutes in acetic acid followed by 70% ethanol. Once the tissues had been cleared, the skeleton was preserved in glycerin. Debris adhering to the puparial integument was removed with pins and brushes and by placing the specimens in an ultrasonic cleaner for a few minutes. The cleaned specimen was mounted on stubs and was examined with a scanning electron microscope (S3000N Hitachi) at 20 kV using variable-pressure (or low vacuum) mode, as this technique allows a direct evaluation of the specimens without coating the samples with gold. The stereomicroscope Olympus SZX16 (equipped with Olympus U-TVO.5XC-3 camera) was used for the examination and to capture images of the puparium (general view) and the cephalopharyngeal skeleton. Dimension measurements (in mm) were performed on preserved specimens using a Leica MZ9.5 binocular microscope.

The terminology for immature stages adopted here follows Rotheray (1993) and Rotheray and Gilbert (1999), whereas certain characters of the cephalopharyngeal skeleton are determined in line with Hartley (1963), and our morphological character descriptions are based on *Merodon* puparia descriptions provided by other authors (Heiss 1938; Stuckenberg 1956; Ricarte et al. 2008, 2017; Preradović et al. 2018). The studied material has been deposited at the University of Alicante, Spain (CEUA).

### Molecular analysis

The specimens subjected to molecular analysis are presented in Supplementary file 8: Table 1. DNA voucher specimens were deposited in FSUNS, EMIT, SZMN, and MZH. The genomic DNA of each specimen was extracted from two or three legs using a slightly modified SDS extraction protocol (Chen et al. 2010). For this purpose, the D2–3 region of the nuclear 28S ribosomal RNA gene and the mitochondrial protein-coding cytochrome c oxidase subunit I (COI) gene were amplified. Primer pair F2 and 3DR was used for the amplification of 28S rRNA gene region (Belshaw et al. 2001), whereas C1-J-2183 (alias Jerry) and TL2-N-3014 (alias Pat) primer pair (Simon et al. 1994) was chosen for 3'-end of COI gene, and for 5'-end COI gene, we used LCO1490 and HCO2198 primer pair (Folmer et al. 1994). The PCR reactions were carried out according to Kočiš Tubić et al. (2018). The amplification products were enzymatically purified by Exonuclease I and FastAP Thermosensitive Alkaline Phosphatase enzymes (ThermoScientific, Lithuania) and sequenced using only forward primers on an ABI3730x1 Genetic Analyzer (Applied Biosystems, Foster City, CA, USA) at the Finnish Institute for Molecular Medicine (FIMM), Helsinki, Finland.

Data analysis

In order to establish the systematic position and composition of the *Merodon serrulatus* group, samples representing the four main *Merodon* lineages were analyzed following the approaches described by Šašić et al. (2016) and Radenković et al. (2018b), while two further Merodontini species served as outgroups, i.e., *Platynochaetus macquarti* Loew, 1862 and *Eumerus grandis* Meigen,1822 (see Supplementary file 8: Table 1 for GB accession numbers of all analyzed species and outgroups). Alignment of the obtained COI sequences was achieved using the Clustal W algorithm (Thompson et al. 1994) implemented in BioEdit (Hall 1999), while rRNA 28S gene was aligned by the multiple alignment using Fast Fourier Transform (MAFFT) program (Katoh et al. 2005, 2009), version 7, which implements iterative refinement methods (Katoh and Standley 2013). The E-INS-i strategy was chosen (Katoh et al. 2009). All sequences in the analyzed two-gene dataset (concatenated COI and 28S rRNA gene sequences) were trimmed to equal lengths. Phylogenetic tree construction was performed by conducting Maximum Parsimony (MP) and Maximum Likelihood (ML) analyses. The parsimony analysis was conducted using NONA (Goloboff 1999), spawned with the aid of ASADO, version 1.85 (Nixon 2008), using the heuristic search algorithm (settings: mult*1,000, hold/100, max trees 100,000, TBR branch swapping). GTRGAMMA model was determined as the best choice model for the analysed dataset using MEGA 7 (Kumar et al. 2016). The dataset was divided into two partitions: COI gene and 28S rRNA gene. The ML tree was constructed by RAxML 8.2.8 (Stamatakis 2014) using the CIPRES Science Gateway (Miller et al. 2010) and applying the general time-reversible (GTR) evolutionary model with a gamma distribution (GTRGAMMA) (Rodríguez et al. 1990). Nodal support was estimated using nonparametric bootstrapping with 1,000 replicates for both MP and ML trees, which were rooted on *Platynochaetus macquarti*.

## Results

### *Merodon serrulatus* group

**Figure 2.**
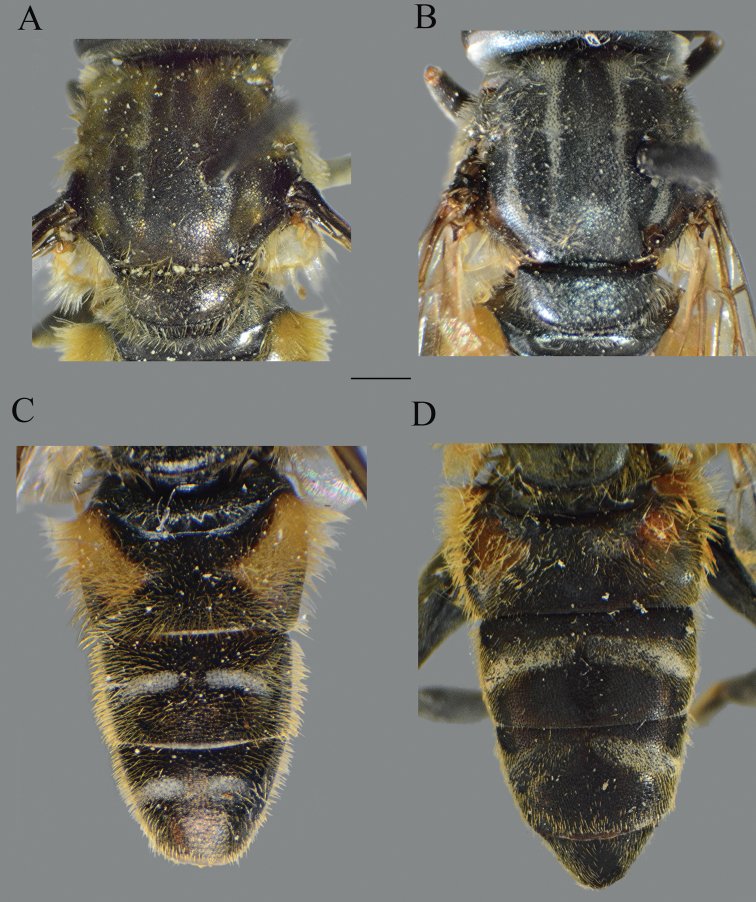
*Merodon serrulatus* body parts, dorsal view. **A** thorax, male **B** thorax, female **C** abdomen, male **D** abdomen, female. Scale bar: 2 mm.

**Diagnosis.** Member species of the *Merodon serrulatus* species group exhibit a distinctive and characteristic basolateral protrusion (lateral hump) on the posterior surstyle lobe (Fig. 1A, B: bp, 6: bp, 14C: bp). They are relatively large (11–15 mm) species with a dark scutum and white microtrichose fasciae (at least in females) on the dark olive brown terga 2–4 (as in Fig. 2); tergum 2 usually with a pair of reddish orange lateral maculae. Antennae dark brown (as in Fig. 5).

**Figure 3.**
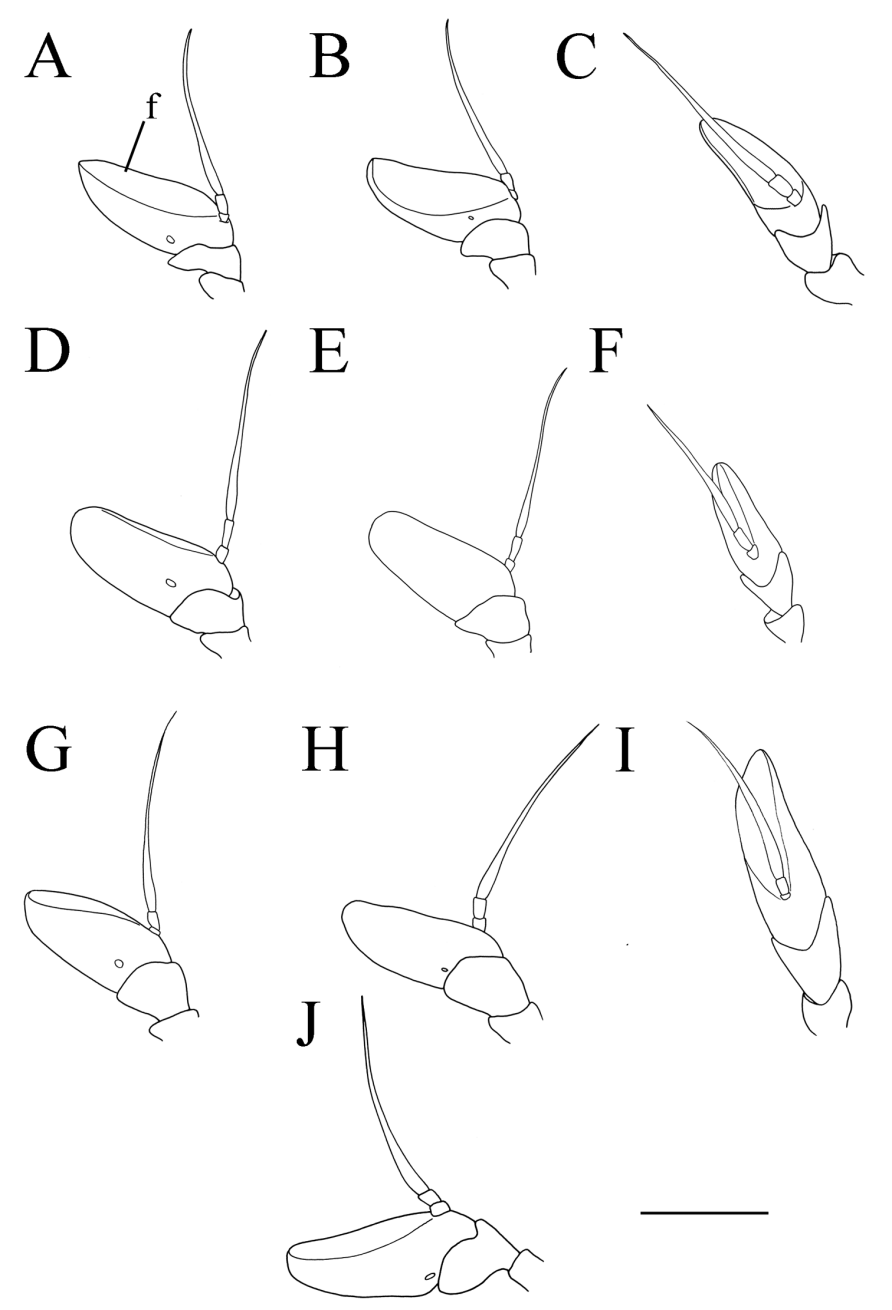
*Merodon serrulatus* antenna. **A** outer side, lateral view, male (Spain) **B** inner side, lateral view, male (Spain) **C** dorsal view, male (Spain) **D** outer side, lateral view, female (Spain) **E** inner side, lateral view, female (Spain) **F** dorsal view, female (Spain) **G** outer side, lateral view, male (Greece) **H** inner side, lateral view, male (Greece) **I** dorsal view, male (Greece) **J** outer side, lateral view, male (Russia). Abbreviation: f–fossette. Scale bar: 1 mm.

**Figure 4.**
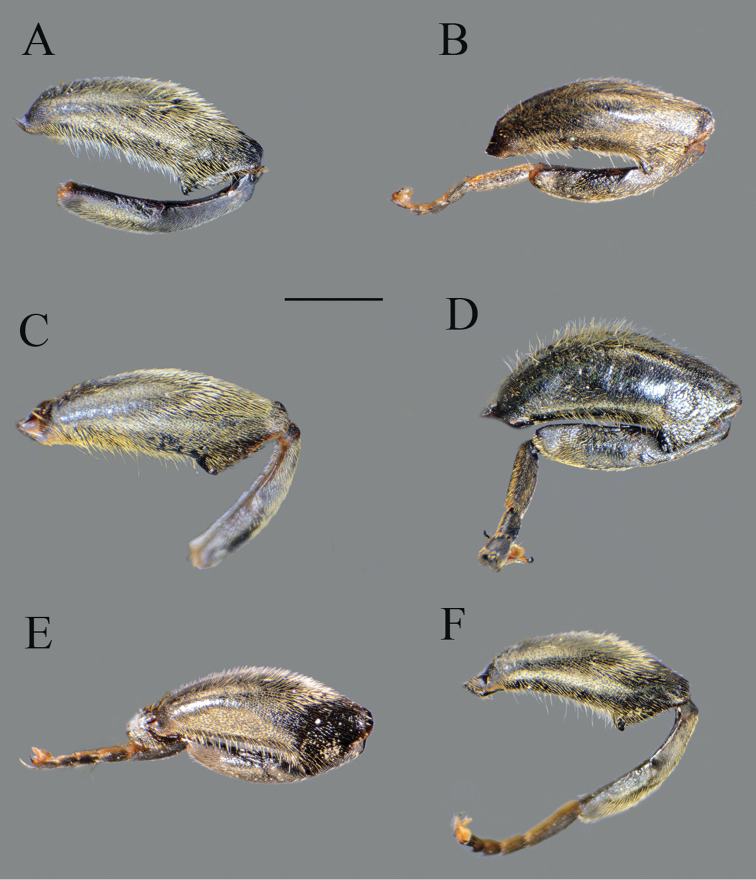
*Merodon serrulatus*, lateral view. **A**, **C** metatrochanter, metafemur and metatibia **B**, **D–F** metaleg. **A–B** male (Spain) **C** female (Spain) **D** male (France) **E** male (Greece) **F** male (Russia). Scale bar: 2 mm.

Basoflagellomere 1.5–2.2 times as long as wide, usually obviously concave dorsally (as in Fig. 3A, D, G, J). Scutum covered with erect, usually yellow pile. Pile on metasternum erect, and as long as those on metacoxa. Posterior part of mesocoxa bare, without long pile. Legs mostly black, without spinae or other protuberances (as in Fig. 4). Metafemora incrassate (as on Fig. 4). Tarsi black dorsally and dark brown ventrally. Abdomen elongated, narrow and tapering (as on Fig. 2), slightly longer than scutum and scutellum together. Male genitalia: apical part of anterior surstyle lobe more or less of rhomboid or triangular in shape (as on Fig. 1A: al, C, D), covered with dense short pile; posterior surstyle lobe oval with basolateral protrusion (lateral hump) (Fig. 1A, B: bp, 14C: bp); cercus rectangular, without prominences (Fig. 1A: c). Hypandrium elongated and sickle shaped (Fig. 1E); lateral sclerite of aedeagus finger-like with basal thorn-like process (Fig. 1G: s); lingula usually present (as on Fig. 1E: l).

**Figure 5.**
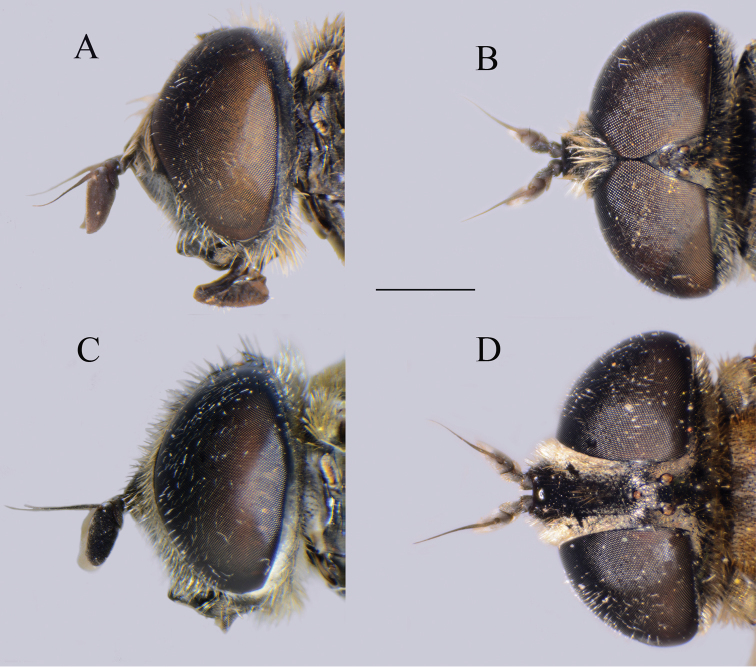
*Merodon serrulatus* head. **A** lateral view, male **B** dorsal view, male **C** lateral view, female **D** dorsal view, female. Scale bar: 2 mm.

**Intraspecific variability**. In most of the taxa in the *Merodon serrulatus* species group, the length of pile on the metafemur and the presence of microtrichia on the scutum and terga is highly variable among specimens of the same species.

The *Merodon serrulatus* species group consists of 13 species, namely *M. bequaerti*, *M. defectus* sp. nov., *M. disjunctus* sp. nov., *M. hirsutus*, *M. kawamurae*, *M. medium* sp. nov., *M. nigrocapillatus* sp. nov., *M. nigropunctum* sp. nov., *M. opacus* sp. nov., *M. sacki*, *M. serrulatus*, *M. sophron* Hurkmans, 1993, and *M. trianguloculus* sp. nov.

### Taxonomy and nomenclature of the species belonging to the *Merodon serrulatus* species group

#### Merodon bequaerti

Taxon classificationAnimaliaDipteraSyrphidae

Hurkmans, 1993

9609A5E4-106F-56A1-850F-210AECB181AE

Figs 8A, B , 9A–C, J , 10A, B , 11A–C

##### Diagnosis.

Large (8–11.9 mm), dark brown species with pairs of narrow microtrichose fasciae on terga 2–4 in males, in some specimens absent; metafemur with long pile on ventral margin; the longest pile as long as one third to half of width of metafemur (Fig. 8); apical part of anterior surstyle lobe rhomboid shape, covered with dense, short pile, and strong dark brown marginal pile on posterior surstyle lobe (Fig. 9A: al, J); females with very narrow microtrichose fasciae on terga 2–4 and sparse pilosity on ventral margin of metafemur, only with few longer pile. Similar to *Merodon sacki* but differs in a less curved metafemur and generally shorter body pile in males, clearly visible on tergum 4 (Fig. 10), and by well separated anterior and posterior surstyle lobe (Fig. 9A), almost fused in *M. sacki* (Fig. 9D). Related to *M. sophron*, but differs in more incrassate metafemur (Fig. 8A, D), longer pile on ventral margin of metafemur in both sexes (Fig. 8), and presence of dense, dark brown marginal pile on apical part of anterior surstyle lobe (Fig. 9A, J), less dense and light yellow in *M. sophron* (Fig. 9G, K) .

**Figure 6.**
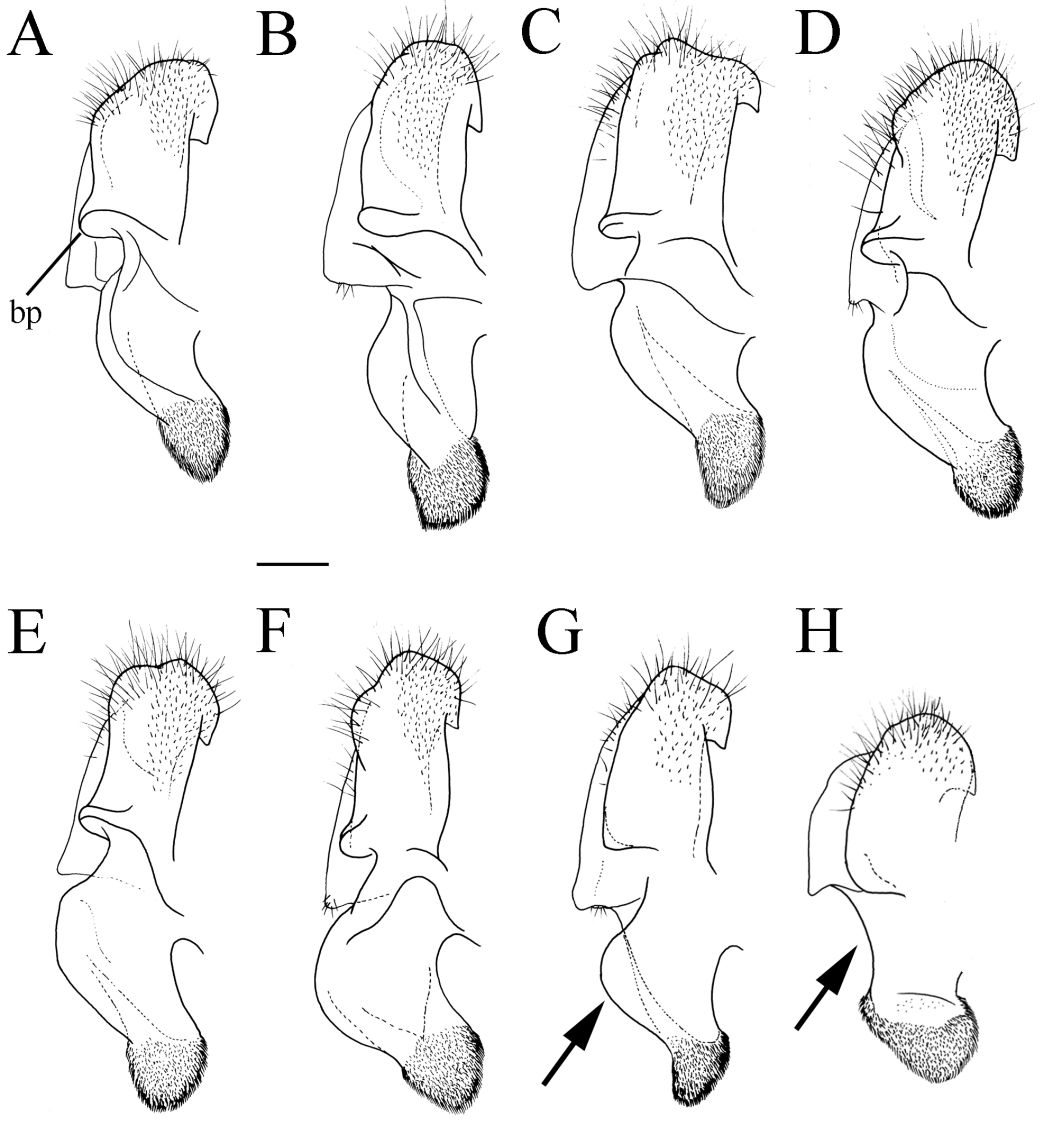
Male genitalia, surstylus, lateral view. **A***Merodon serrulatus*, Spain **B***Merodon serrulatus*, France **C***Merodon serrulatus*, Greece (Pindos) **D***Merodon serrulatus*, Greece (Olympos) **E***Merodon serrulatus*, Greece (Peloponnese) **F***Merodon serrulatus*, Montenegro **G***Merodon serrulatus*, Russia **H***Merodon medium* sp. nov. G, **H** margin of anterior surstyle lobe marked with arrow. Abbreviation: bp–basolateral protrusion. Scale bar: 0.2 mm.

**Figure 7.**
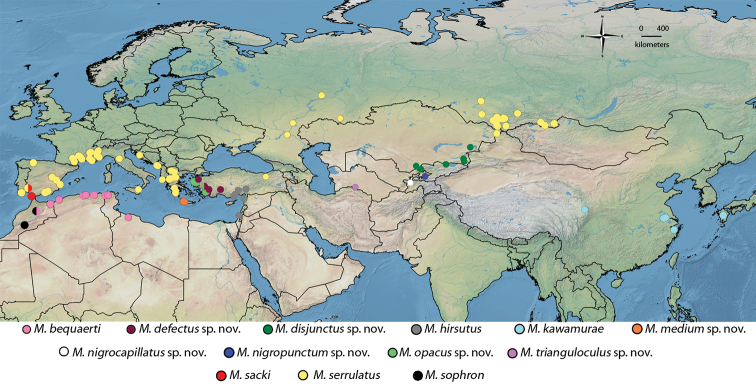
Distribution map of *Merodon serrulatus* species group.

**Figure 8.**
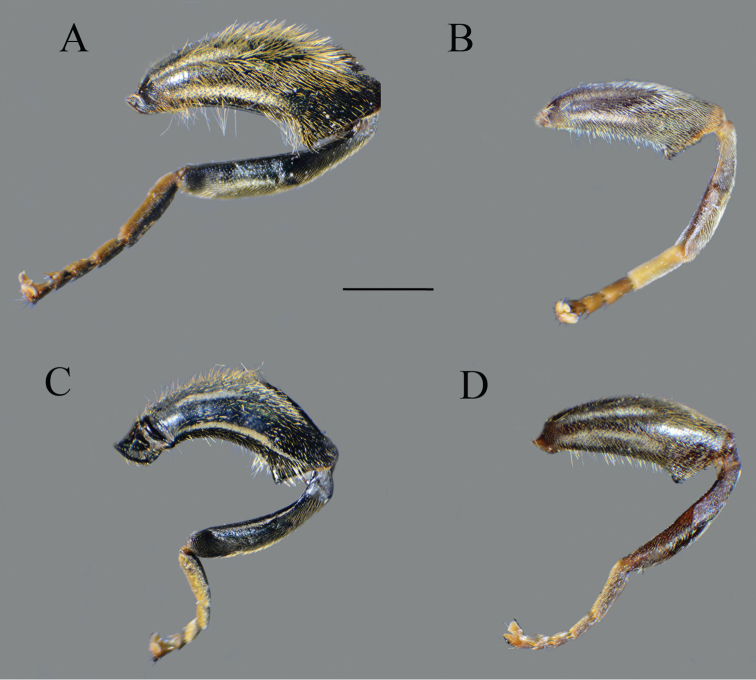
Metaleg, lateral view. **A***Merodon bequaerti*, male **B***Merodon bequaerti*, female **C***Merodon sacki*, male **D***Merodon sophron*, male. Scale bar: 2 mm.

**Figure 9.**
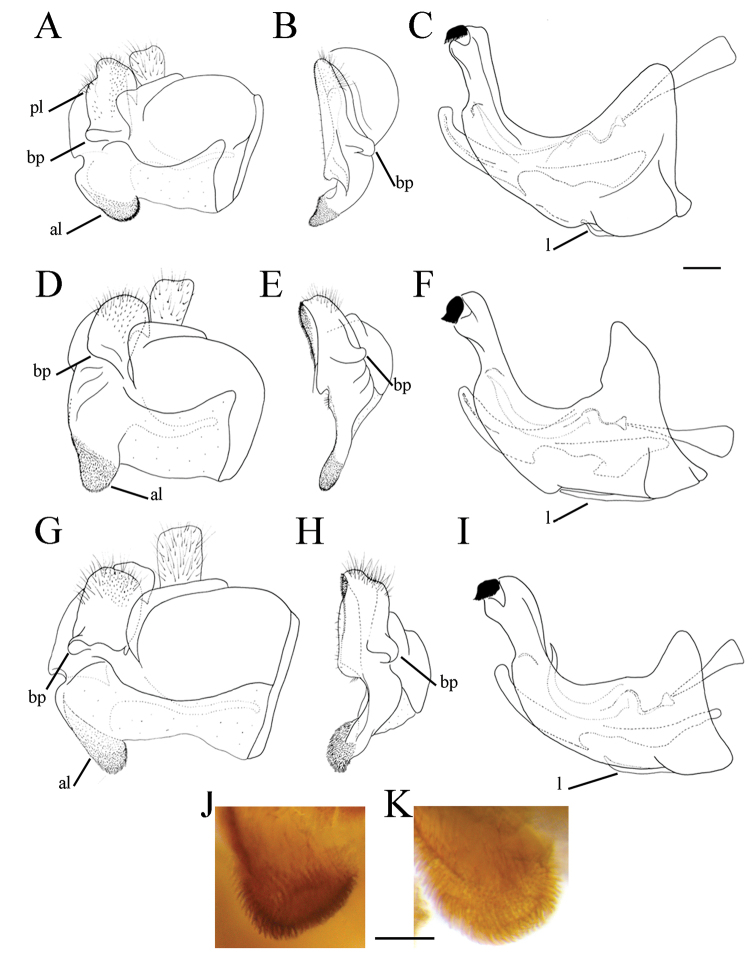
Male genitalia. **A***Merodon bequaerti*, epandrium, lateral view **B***Merodon bequaerti*, epandrium, ventral view **C***Merodon bequaerti*, hypandrium, lateral view **D***Merodon sacki*, epandrium, lateral view **E***Merodon sacki*, epandrium, ventral view **F***Merodon sacki*, hypandrium, lateral view **G***Merodon sophron*, epandrium, lateral view **H***Merodon sophron*, epandrium, ventral view **I***Merodon sophron*, hypandrium, lateral view **J***Merodon bequaerti*, anterior surstyle lobe, lateral view **K***Merodon sophron*, anterior surstyle lobe, lateral view. Abbreviations: al–anterior surstyle lobe, bp–basolateral protrusion, l–lingula, pl–posterior surstyle lobe. Scale bar: 0.2 mm.

**Figure 10.**
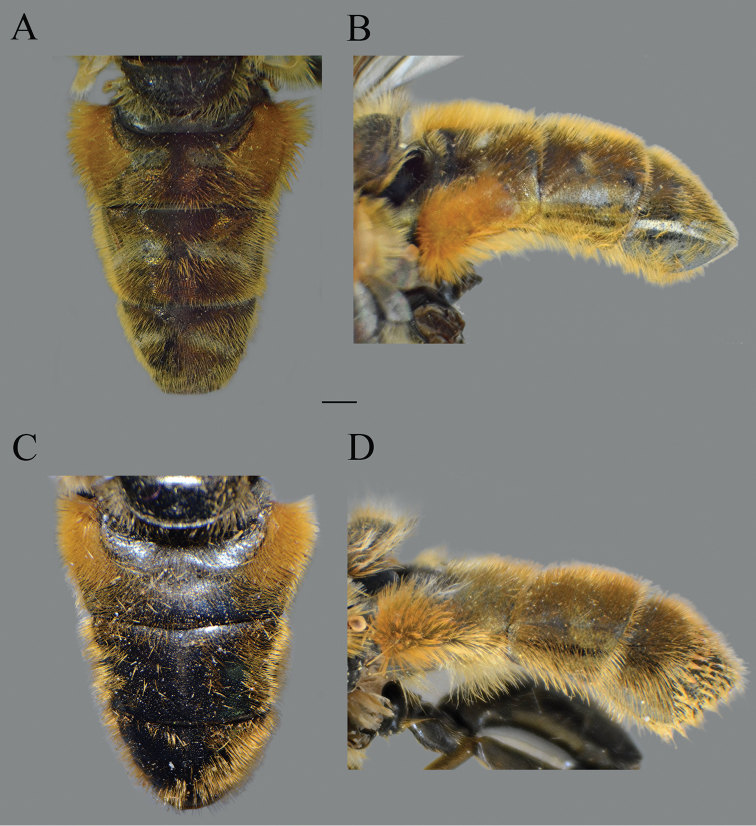
Abdomen of males. **A***Merodon bequaerti*, dorsal view **B***Merodon bequaerti*, lateral view **C***Merodon sacki*, dorsal view **D***Merodon sacki*, lateral view. Scale bar: 2 mm.

##### Redescription

**Figure 11.**
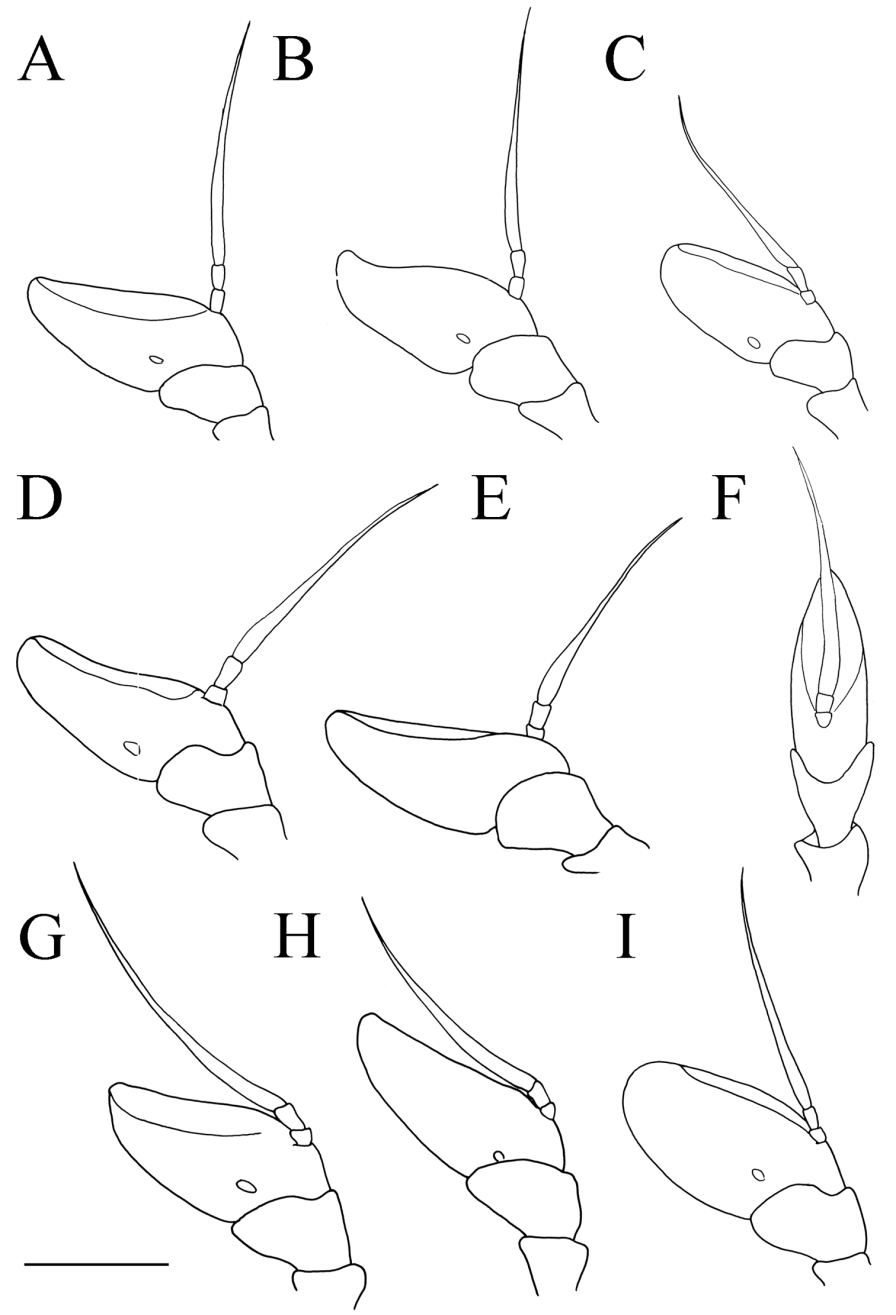
Antenna. **A***Merodon bequaerti*, outer side, lateral view, male **B***Merodon bequaerti*, inner side, lateral view, male **C***Merodon bequaerti*, outer side, lateral view, female **D***Merodon sacki*, outer side, lateral view, male **E***Merodon sacki*, inner side, lateral view, male **F***Merodon sacki*, dorsal view, male **G***Merodon sophron*, outer side, lateral view, male **H***Merodon sophron*, inner side, lateral view, male **I***Merodon sophron*, outer side, lateral view, female. Scale bar: 1 mm.

(based on the type material and additional specimens). **Male.** Head. Antennae black to dark brown; basoflagellomere 1.7–2.1 times as long as wide, and 2.3 times as long as pedicel, concave dorsally with acute apex; fossette dorsolateral (Fig. 11); arista dark and thickened at basal one third, covered with dense microtrichia; arista 1.4–1.7 times as long as basoflagellomere (Fig. 11A, B); face and frons black with gray microtrichia, face covered with dense whitish gray, and frons with yellowish gray pile; oral margin shiny with microtrichose lateral areas; lunule shiny black, bare; eye contiguity 10–12 facets long; vertex isosceles, shiny covered with golden microtrichia in front of ocellar triangle; vertex with long, pale whitish yellow pile mixed with black pile on the ocellar triangle; ocellar triangle equilateral; occiput shiny, with gray-yellow pile, covered with a dense, gray microtrichia in ventral half; eyes covered with dense pile.

Thorax. Scutum and scutellum black with bronze luster, covered with dense, erect, yellow pile; scutum at wing basis with short black pile, in some specimens with fascia of black pile between wing basis; scutum with two or more microtrichose vittae, anteriorly connected and posteriorly reaching the scutellum; posterodorsal part of anterior anepisternum, posterior anepisternum (except anteroventral angle), anterior anepimeron, dorsomedial anepimeron, and posterodorsal and anteroventral parts of katepisternum with long, pale yellow pile and grayish microtrichia; wings entirely covered with microtrichia; wing veins brown; calypteres and halteres pale yellow; legs without spinae or other protuberances; legs mostly black, except brown tarsi ventrally in some specimens; pile on legs pale yellow, except black pile at apical one fourth of metafemur; metafemur curved and incrassate, ca. three times longer than wide; pile on postero- and anteroventral surface long, and ca. one third to half of width of metafemur, slightly longer than pile on dorsal margin (Fig. 8A).

Abdomen. Wide, tapering, 1.2 times longer than mesonotum; terga dark brown to black, with or without pairs of narrow microtrichose fasciae; tergum 2 with orange lateral maculae; pile on terga all yellow, except few black pile on medial part of terga 3 and 4 in some specimens (Fig. 10A, B); sterna dark brown, covered with long whitish/yellow pile.

Male genitalia. Apical part of anterior surstyle lobe rhomboid shape, 1.5 times longer than wide, covered with dense, short pile, and strong dark brown marginal pile (Fig. 9A: al, J); posterior surstyle lobe oval (Fig. 9A: pl) with basolateral protrusion (lateral hump) (Fig. 9A, B: bp); hypandrium sickle-shaped, without lateral projections; lingula small (Fig. 9C: l).

**Female.** Similar to the male except for normal sexual dimorphism and for the following characteristics: antennae with rounded tip, basoflagellomere 1.7–1.9 times longer than wide (Fig. 11C); frons with microtrichose vittae along eye margins; frons covered with pilosity of variable color, from mostly gray-yellow until predominantly black; ocellar triangle covered with black pile; ventral margin of metafemur with sparse pilosity, only few pile longer (Fig. 8B); lateral side of terga, anterior two third of tergum 2 and all tergum 5 with yellow pile; terga 2–4 with short adpressed black pile and with very narrow microtrichose fascia.

##### Distribution.

*Merodon bequaerti* is distributed in north-western Africa (Algeria, Libya, Morocco, and Tunisia) (Fig. 7).

##### Ecology.

Preferred environment: unimproved montane grassland, including open, grassy areas in pine forest or Mediterranean scrub. Flowers visited: no data. Flight period: February-June.

##### Type material.

**Holotype** [original designation by Hurkmans (1993: 194)]: male. Original label: “*Merodon bequaerti* / *spec. nov. HOLOTYPE* / ♂. *W. Hurkmans 1988*.” [red label handwritten], “*Merodon / parietum* / *Mg* ♂” [label handwritten], “Noiseux Oran / Algeria / Dr. J. Bequaert” “23-IV-l0” [handwritten on the back side] (MNHN) (See Supplementary file 1: Figure 1A). **Paratype**: female. Same label data as holotype (MNHN) (studied).

##### Other material.

Algeria • 1 ♂; Kabylie, Tikjda; 36°27'00"N, 4°07'60"E; 28 Jun. 1954; NBCN • 1 ♂; Jijel, Oued el Kebir; 36°35'22"N, 6°16'16"E; 20 May 1981; NBCN • 1 ♂; Jijel, Foce Oued El Kebir; 36°35'45"N, 6°15'29"E; 20 May 1981; I. Aslan leg.; NBCN 05636 • 1 ♀; El Kseur, Akfadou; 36°37'60"N, 4°36'00"E; 22–23 May 1981; NBCN 04079.

Libya • 1 ♀; Tripolitania, Garian; 32°10'46"N, 13°01'53"E; “2.500 feet” [760 m a.s.l.]; 22 Feb. 1954; K. M. Guichard leg.; NHMUK 04353.

Morocco • 1 ♂; Moyen Atlas, Azrou; 33°25'48"N, 5°12'36"W; 15 Jun. 1928; R. Benoist leg.; MNHN 22623 • 8 ♂♂; Mountain de Beni-Snassen 2; 34°48'43"N, 2°24'08"W; 29 Apr. 2013; A. Vujić, S. Radenković leg.; FSUNS Đ13, Đ14, Đ16 to Đ21.

Tunisia • 1 ♂; Jundubah, 40 km W from Jendouba; 36°31'54"N, 8°28'25"E; 17 May 1988; ZMUC 02497 • 1 ♂; same data as for preceding; 36°34'33"N, 9°02'12"E; ZMUC 02498.

#### Merodon defectus

Taxon classificationAnimaliaDipteraSyrphidae

Vujić, Likov & Radenković sp. nov.

58FE4CBE-E842-5367-A69C-1AD62C713593

http://zoobank.org/7EDC43D1-8B17-46D0-8C28-B18485482741

**Figure 12.**
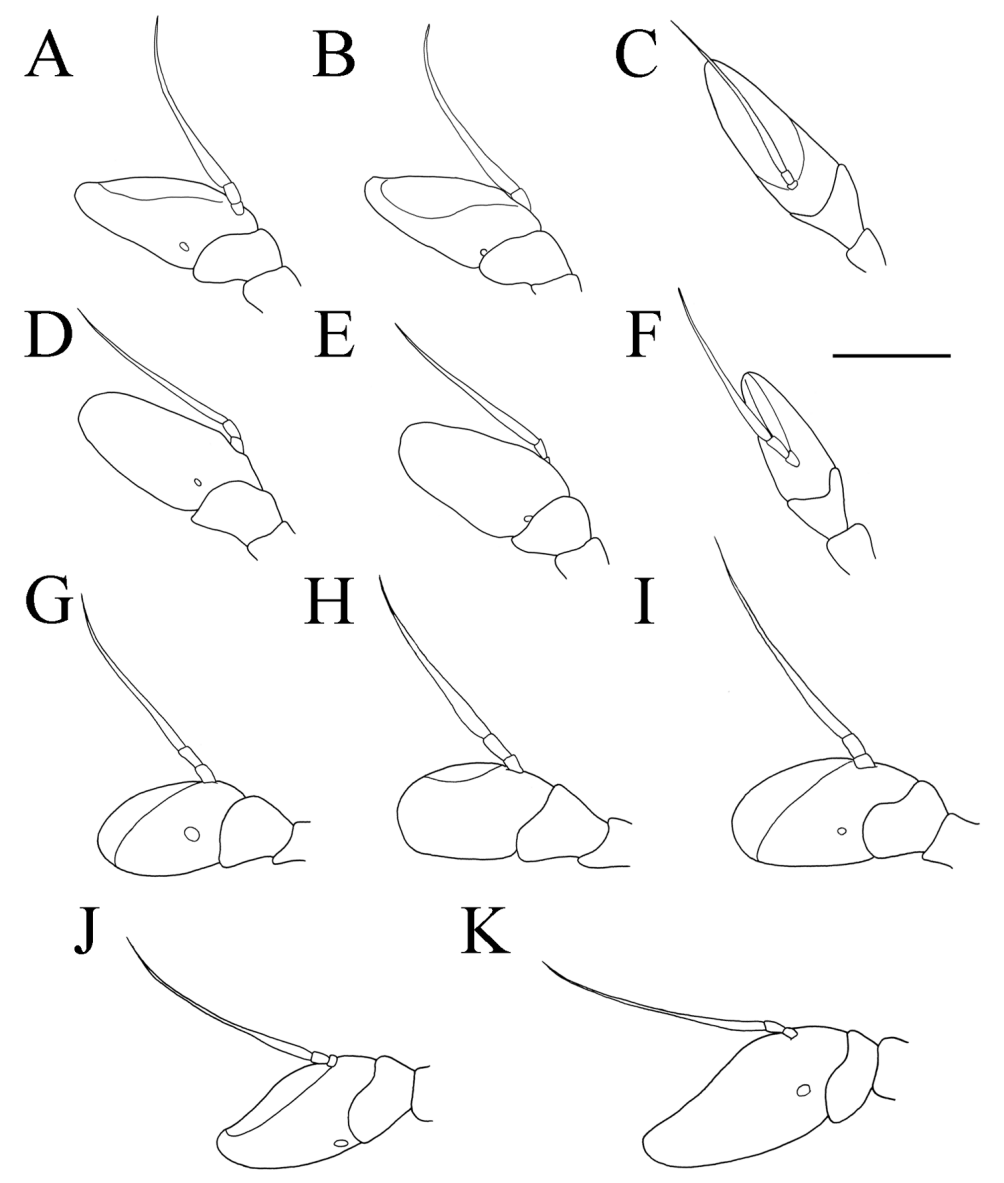
Antenna. **A***Merodon opacus* sp. nov., outer side, lateral view, male **B***Merodon opacus* sp. nov., inner side, lateral view, male **C***Merodon opacus* sp. nov., dorsal view, male **D***Merodon opacus* sp. nov., outer side, lateral view, female **E***Merodon opacus* sp. nov., inner side, lateral view, female **F***Merodon opacus* sp. nov., dorsal view, female **G***Merodon disjunctus* sp. nov., outer side, lateral view, male **H***Merodon disjunctus* sp. nov., inner side, lateral view, male **I***Merodon disjunctus* sp. nov., outer side, lateral view, female **J***Merodon defectus* sp. nov., outer side, lateral view, male **K***Merodon defectus* sp. nov., outer side, lateral view, female. Scale bar: 1 mm.

**Figure 13.**
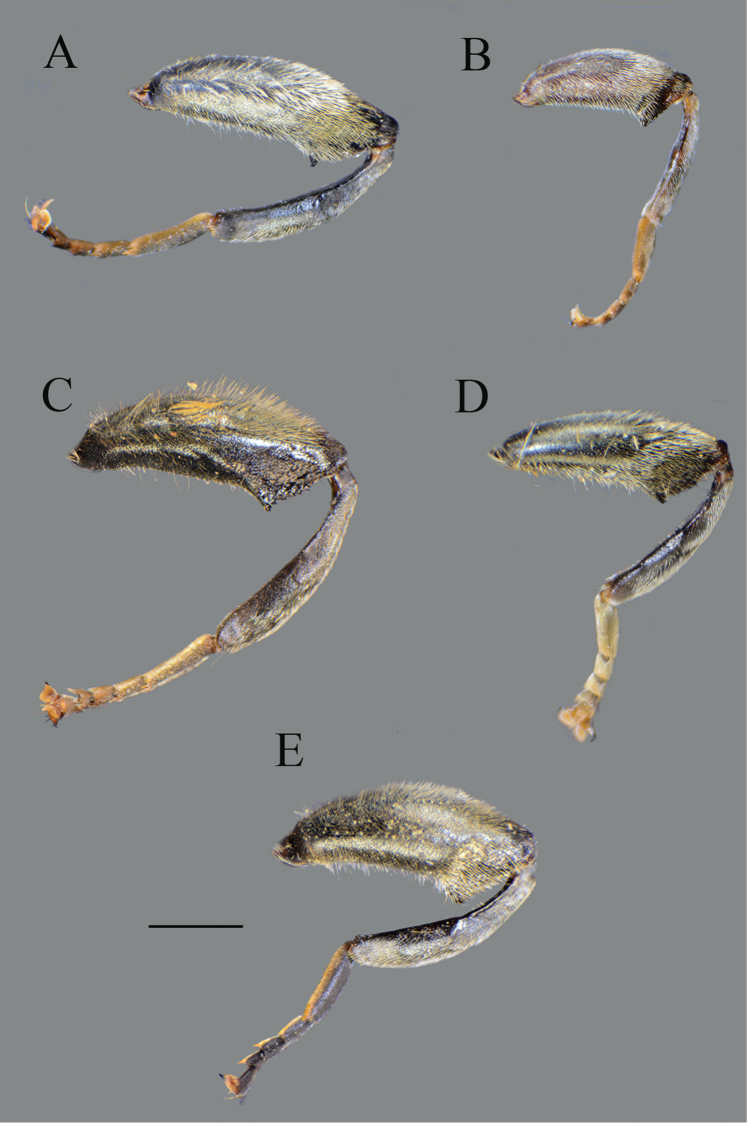
Metaleg, lateral view. **A***Merodon opacus* sp. nov., male **B***Merodon opacus* sp. nov., female **C***Merodon hirsutus*, male **D***Merodon hirsutus*, female **E***Merodon defectus* sp. nov., male. Scale bar: 2 mm.

**Figure 14.**
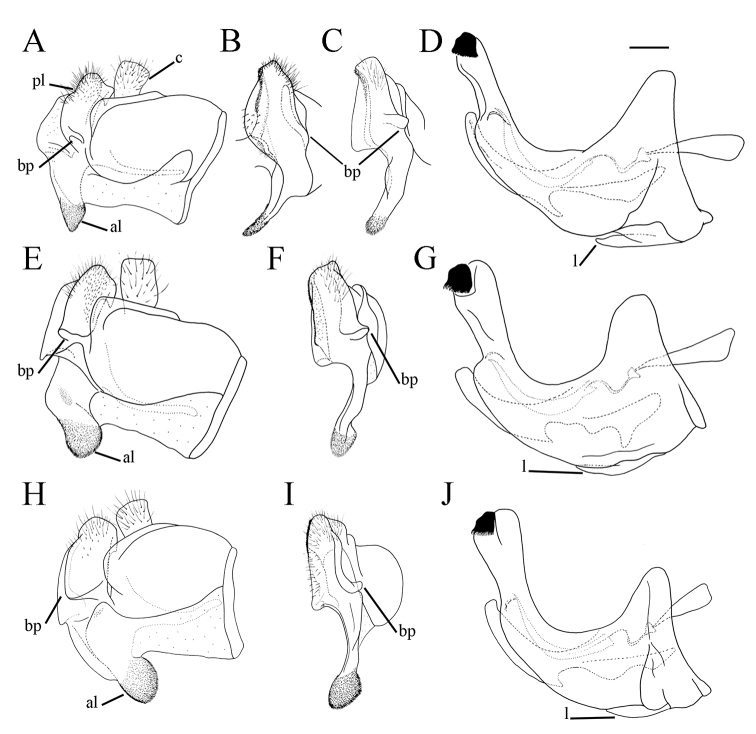
Male genitalia. **A***Merodon defectus* sp. nov., epandrium, lateral view **B***Merodon defectus* sp. nov., epandrium, ventral view **C***Merodon serrulatus*, epandrium, ventral view **D***Merodon defectus* sp. nov., hypandrium, lateral view **E***Merodon hirsutus*, epandrium, lateral view **F***Merodon hirsutus*, epandrium, ventral view **G***Merodon hirsutus*, hypandrium, lateral view, **H***Merodon opacus* sp. nov., epandrium, lateral view **I***Merodon opacus* sp. nov., epandrium, ventral view **J***Merodon opacus* sp. nov., hypandrium, lateral view. Abbreviations: al–anterior surstyle lobe, bp–basolateral protrusion, c–cercus, l–lingula, pl–posterior surstyle lobe. Scale bar: 0.2 mm.

**Figure 15.**
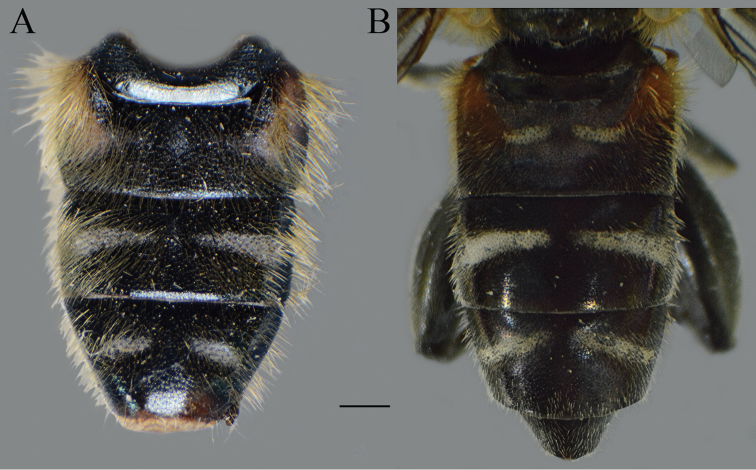
Abdomen, dorsal view. **A***Merodon disjunctus* sp. nov., male **B***Merodon defectus* sp. nov., female. Scale bar: 2 mm.

**Figure 16.**
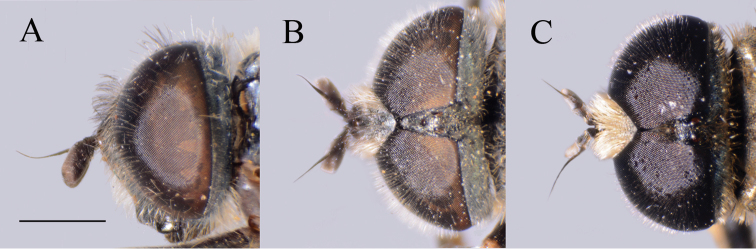
Head of male. **A***Merodon disjunctus* sp. nov., lateral view **B***Merodon disjunctus* sp. nov., dorsal view **C***Merodon defectus* sp. nov., dorsal view. Scale bar: 2 mm.

Figs 12J, K , 13E , 14A, B, D , 15B , 16C

##### Diagnosis.

Medium sized (7.6–10.9 mm), dark species with olive-brown reflection; antennae dark brown; legs mostly black; basoflagellomere elongated (in males 1.8 times as long as wide) obviously concave dorsally; arista short, 1.6–1.8 times as long as basoflagellomere (Fig. 12J, K); terga dark brown to black, except pale yellow-orange lateral maculae on tergum 2; metafemur incrassate, ventrally covered with pilosity of medium length (Fig. 13E); male genitalia: posterior surstyle lobe with very small lateral hump (Fig. 14A, B: bp); apical part of anterior surstyle lobe triangular (Fig. 14A: al); lingula medium sized (Fig. 14D: l). Similar to *Merodon serrulatus* from which differs in reduced lateral hump on posterior surstyle lobe (Fig. 14A, B: bp) (in *M. serrulatus* distinct, Fig. 1A, B: bp, 14C: bp). Morphologically related to *M. opacus* sp. nov. and *M. hirsutus* from which can be distinguished by the presence of yellow-orange lateral maculae on tergum 2 (in *M. opacus* sp. nov. and *M. hirsutus* tergum 2 dark). Additionally, differing from *M. hirsutus* by shorter dorsolateral pile on metafemur (Fig. 13E) and posterior surstyle lobe with very small lateral hump (Fig. 14A, B: bp), well developed in *M. hirsutus* (Fig. 14E, F: bp) and *M. opacus* sp. nov. (14H, I: bp).

##### Description.

**Male.** Head. Antennae black to dark brown; basoflagellomere elongated, 1.8 times as long as wide, and 2.3 times as long as pedicel, concave dorsally with acute apex; dorsolateral fossette large; arista dark brown and thickened at basal one third, covered with dense microtrichia, 1.6–1.8 times as long as basoflagellomere (Fig. 12J); face black with gray microtrichia, covered with whitish pile; oral margin microtrichose, with small, shiny, lateral bare area; lunule shiny black; frons microtrichose, with yellowish gray pile; eye contiguity ca. eight facets long; vertex isosceles, with long, pale whitish yellow pile, mixed with black pile on the ocellar triangle; ocellar triangle isosceles (Fig. 16C); occiput shiny, with gray-yellow pile; eyes covered with dense pile; vertical triangle: eye contiguity: frons = 3 : 1 : 3.

Thorax. Scutum and scutellum black with bronze luster, covered with dense, erect, yellow pile; scutum at wing basis with short black pile and some black pile between wing basis, from few ones to fascia of black pilosity in some specimens; scutum usually with two or more microtrichose vittae, anteriorly connected and posteriorly reaching the scutellum; scutum dull; posterodorsal part of anterior anepisternum, posterior anepisternum (except anteroventral angle), anterior anepimeron, dorsomedial anepimeron, and posterodorsal and anteroventral parts of katepisternum with long, pale yellow pile and grayish microtrichia; wings entirely covered with microtrichia; wing veins brown; calypteres yellowish; halteres brown-yellow; legs mostly black, except brown tarsi ventrally in some specimens; pile on legs gray-yellow; metafemur moderately incrassate, ca. three times longer than wide; pile on postero- and anteroventral surface of medium length; pile on dorsolateral surface dense and length ca. one third to one fourth of width of metafemur (Fig. 13E).

Abdomen. Tapering, 1.2 times longer than mesonotum; terga dark brown to black, except pale yellow-orange lateral maculae on tergum 2; terga 2–4 each with a pair of white microtrichose, oblique fasciae (on tergum 2 triangular); pile on terga long, yellow, except some black pile on terga 3 and 4 medially; sterna dark brown, covered with long whitish yellow pile.

Male genitalia. Apical part of anterior surstyle lobe triangular, ca. two times longer than wide, covered with dense, short pile (Fig. 14A: al); posterior surstyle lobe with very small basolateral protrusion (Fig. 14A, B: bp); hypandrium sickle-shaped, without lateral projections; lingula medium size (Fig. 14D: l).

**Female.** Similar to the male except for normal sexual dimorphism and for the following characteristics: basoflagellomere ca. two times longer than wide (Fig. 12K), fossette dorsal; frons with broad microtrichose vittae along eye margins; frons covered with pilosity of variable color, from mostly gray-yellow to predominantly black; ocellar triangle covered with black pile; terga with whitish pile, except terga 2–5 medially with short black pile; microtrichose fasciae on terga 3 and 4 conspicuous (Fig. 15C).

##### Etymology.

Latin adjective *defectus* (reduced in size, smaller) refers to small basolateral protrusion (lateral hump) on posterior surstyle lobe.

##### Distribution.

*Merodon defectus* sp. nov. has been identified in western Turkey (Fig. 7).

##### Ecology.

Preferred environment: forest/open ground; thermophilous and evergreen *Quercus* forest; *Castanea* forest, dry *Pinus* forest; unimproved grassland and tracksides; coniferous forest with some yellow flowers along a stream [Reemer and Smit (2007) refer to this last observation as being *Merodon alexeji* Paramonov, 1925]. Flowers visited: *Ornithogalum* spp., *Potentilla* spp., and *Thymus* spp. Flight period: May-July.

##### Type material.

**Holotype**. Turkey • ♂; Bozdağ mountain, Near Bozdağ; 38°22'28"N, 28°04'38"E; 1140 m a.s.l.; 7 Jun. 2014; A. Vujić, J. Ačanski leg.; FSUNS 06950. Original label: “HOLOTYPE of *Merodon* / *defectus* Vujić, Likov et / Radenković sp.n. 2019” [red label], “Turkey, Bozdağ Mountain, / near Bozdağ 7/6/2014 / 38.374523 28.077339 1140m / Leg. Vujić, Ačanski”, “AU305”, “06950” (See Supplementary file 2: Figure 2A). **Paratypes**. Turkey • 1 ♂; Muğla, 14 km NE from Ağla, Lake Kartar; 37°01'50"N, 28°45'09"E; 1600 m a.s.l.; 31 May 2000; J. T. Smit leg.; J. T. S. coll. 04066 [published in Reemer and Smit (2007) under name *Merodon alexeji*] • 1 ♀; Muğla, 14 km NE from Ağla, Lake Kartar; 37°01'50"N, 28°45'09"E; 1600 m a.s.l.; 31 May 2000; J. T. Smit leg.; J. T. S. coll. 04067 [published in Reemer and Smit (2007) under name *Merodon alexeji*] • 4 ♀♀; Isparta, Yenişarbademli, Melikler Yaylası 2; 37°41'38"N, 31°17'56"E; 1770 m a.s.l.; 21 Jun. 2016; R. Hayat, A. Vujić, O. Demirözer, J. Ačanski leg.; EMIT 12301 to 12304 • 1 ♂; Babadağ, Near Denizli valley I; 37°41'43"N, 28°59'35"E; 1870 m a.s.l.; 5 Jul. 2015; A. Vujić, S. Radenković, J. Ačanski, S. Gökhan, N. Veličković leg.; FSUNS 09774 • 2 ♀♀; Babadağ, Near Denizli valley I; 37°41'43"N, 28°59'35"E; 1870 m a.s.l.; 5 Jul. 2015; A. Vujić, S. Radenković, J. Ačanski, S. Gökhan, N. Veličković leg.; FSUNS 09773, 09775 • 3 ♂♂; Isparta, Yenişarbademli, Melikler Yaylası; 37°41'52"N, 31°17'39"E; 1730 m a.s.l.; 30 Jun. 2015; A. Vujić, R. Hayat, O. Dermirözer, A. Uzal leg.; EMIT 09953 to 09955 • 3 ♀♀; Isparta, Yenişarbademli, Melikler Yaylası; 37°41'52"N, 31°17'39"E; 1730 m a.s.l.; 30 Jun. 2015; A. Vujić, R. Hayat, O. Demirözer, A. Uzal leg.; EMIT 09952, 09956, 09957 • 8 ♂♂; Isparta, Yenişarbademli, Melikler Yaylası 1; 37°41'52"N, 31°17'39"E; 1730 m a.s.l.; 21 Jun. 2016; R. Hayat, A. Vujić, O. Demirözer, J. Ačanski leg.; EMIT 12249 to 12251, 12256, 12257, 12260, 12264 • 9 ♀♀; Isparta, Yenişarbademli, Melikler Yaylası 1; 37°41'52"N, 31°17'39"E; 1730 m a.s.l.; 21 Jun. 2016; R. Hayat, A. Vujić, O. Demirözer, J. Ačanski leg.; EMIT 12253 to 12255, 12258, 12259, 12261 to 12263, 12265 • 3 ♀♀; Babadağ, Near Denizli on the top; 37°42'33"N, 28°59'23"E; 2060 m a.s.l.; 5 Jul. 2015; A. Vujić, S. Radenković, J. Ačanski, S. Gökhan, N. Veličković leg.; FSUNS 09769, 09770, 09772 • 1 ♀; Bozdağ mountain, Near Bozdağ; 38°19'58"N, 28°06'35"E; 1570 m a.s.l.; 7 Jun. 2014; A. Vujić, J. Ačanski leg.; FSUNS 06931 • 8 ♂♂; Bozdağ mountain, Near Bozdağ; 38°20'50"N, 28°04'08"E; 1170 m a.s.l.; 7 Jun. 2014; A. Vujić, J. Ačanski leg.; FSUNS 06952, 06953, 06956, 06957, 06959 to 06962 • 6 ♀♀; Bozdağ mountain, Near Bozdağ; 38°20'50"N 28°04'08"E; 1170 m a.s.l.; 7 Jun. 2014; A. Vujić, J. Ačanski leg.; FSUNS 06954, 06955, 06958, 06963 to 06965 • 1 ♂; Balıkesir, Edremit-Akçay; 39°40'40"N, 26°54'09"E; 27 Jul. 2015; J. Devalez leg.; MAegean 10131.

#### Merodon disjunctus

Taxon classificationAnimaliaDipteraSyrphidae

Vujić, Likov & Radenković sp. nov.

59A710EB-EF01-5A65-BD4E-446B6CB7A80E

http://zoobank.org/2A04080B-F1B9-4CE1-A2B1-42AECB902F65

**Figure 17.**
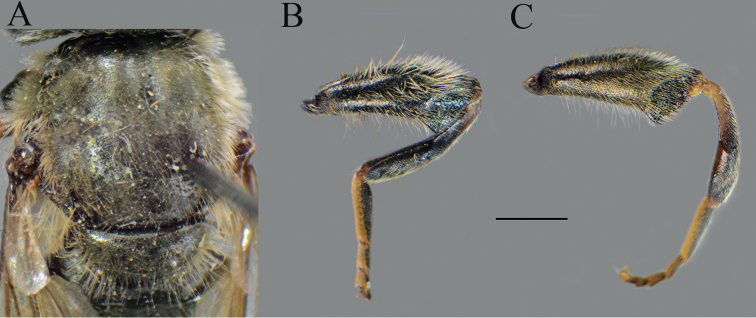
*Merodon disjunctus* sp. nov. **A** thorax, dorsal view, male **B** metaleg, lateral view, male **C** metaleg, lateral view, female. Scale bar: 2 mm.

**Figure 18.**
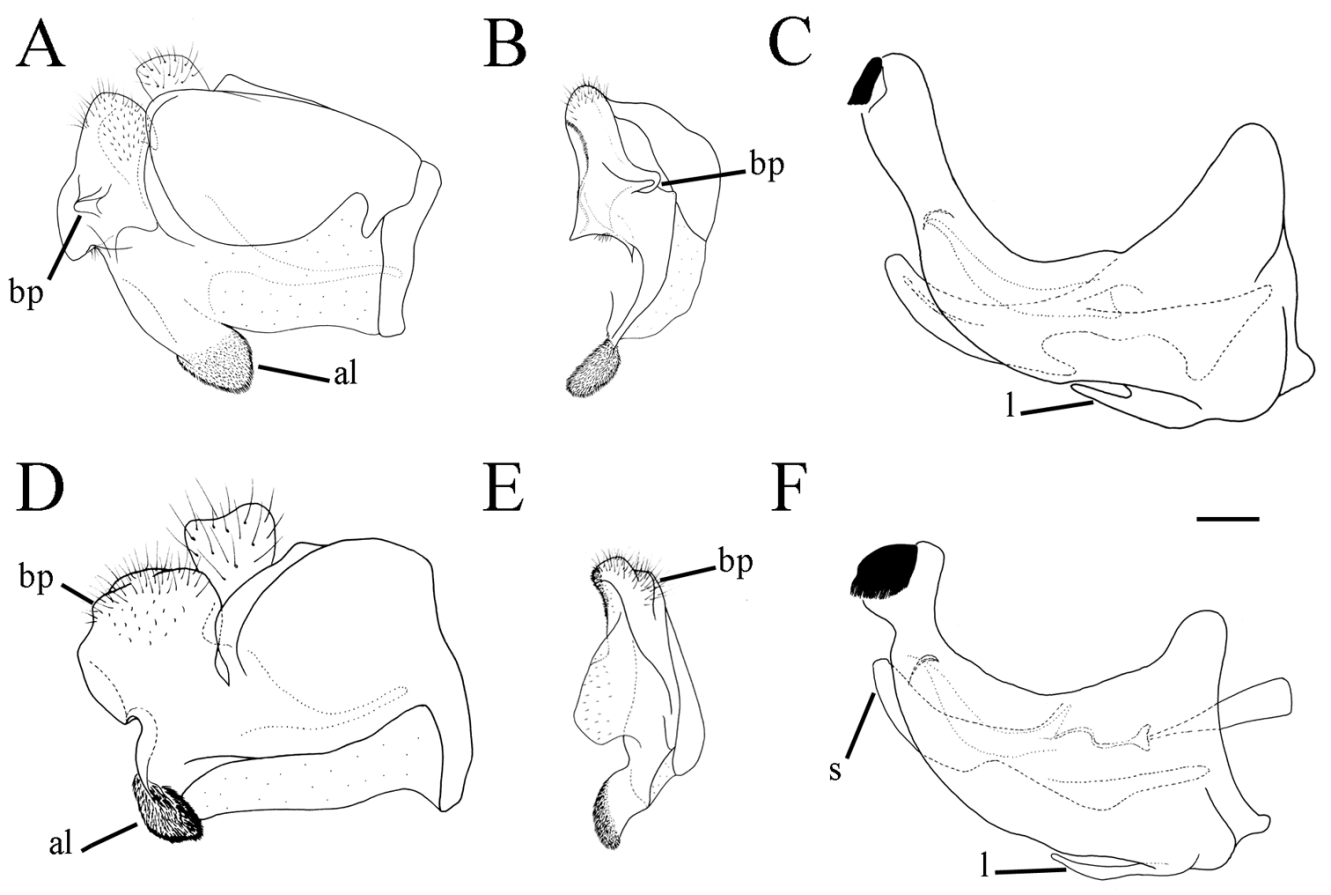
Male genitalia. **A***Merodon disjunctus* sp. nov., epandrium, lateral view **B***Merodon disjunctus* sp. nov., epandrium, ventral view **C***Merodon disjunctus* sp. nov., hypandrium, lateral view **D***Merodon kawamurae*, epandrium, lateral view **E***Merodon kawamurae*, epandrium, ventral view **F***Merodon kawamurae*, hypandrium, lateral view. Abbreviations: al–anterior surstyle lobe, bp–basolateral protrusion, l–lingula, pl–posterior surstyle lobe, s–lateral sclerite of aedeagus. Scale bar: 0.2 mm.

Figs 12G–I , 15A , 16A, B , 17A–C , 18A–C

##### Diagnosis.

Medium sized (8.5–10.8 mm), dark, olive-brown species, covered with pale yellow pile; males with dichoptic eyes, separated by a distance of 3–5 facets (Fig. 16B); terga 2–4 with pairs of white microtrichose fasciae, differently developed (from conspicuous to vague) (Fig. 15A); in male basoflagellomere short, 1.2 times longer than wide, with large fossette extending to the apex of basoflagellomere (Fig. 12G–I).

##### Description.

**Male.** Head. Antennae black; basoflagellomere short, 1.2 times as long as wide, and ca. 1.7 times as long as pedicel, and with rounded apex; large fossette dorsomedial and dorsolateral including apex of basoflagellomere (Fig. 12G); arista black and thickened at basal one third, covered with dense microtrichia, 1.5 times as long as basoflagellomere (Fig. 12G–H); face and frons black with gray microtrichia; face covered with dense whitish gray, while frons with mostly black pile (Fig. 16A); oral margin shiny, with lateral microtrichose area; lunule shiny black, bare; eye dichoptic, separated by distance of 3–5 facets (Fig. 16B); vertex isosceles, covered with dark gray microtrichia and long, black pile; ocellar triangle equilateral; occiput shiny covered with black pile in upper half, ventrally with gray-yellow pile and dense, gray microtrichia; eyes covered with dense pile.

Thorax. Scutum and scutellum dull, with bronze luster, covered with dense, erect, yellowish pile, except posterior half medially with few to many black pile intermixed; in some specimens scutellum with few black pile; scutum with indistinct microtrichose vittae (Fig. 17A); posterodorsal part of anterior anepisternum, posterior anepisternum (except anteroventral angle), anterior anepimeron, dorsomedial anepimeron, and posterodorsal and anteroventral parts of katepisternum with long, dark gray pile and grayish microtrichia; wings entirely covered with microtrichia; wing veins yellow-brown; calypteres and halteres whitish yellow; legs black, except yellow-brown tarsi, tip of femora and basal part of tibiae; pile on legs mostly yellowish; metafemur moderate incrassate, ca. five times longer than wide; pile on postero- and anteroventral surface long, ca. half to two thirds of width of metafemur, as same length as pile dorsally (Fig. 17B).

Abdomen. Tapering, ca. 1.2 times longer than mesonotum; terga dark brown to black, except for a pair of yellow-orange, triangular, lateral maculae on tergum 2; terga 2–4 with conspicuous or with trace of white microtrichose pair of fasciae (variable character); pile on terga mostly yellow, except terga 3 and 4 medially with black pile; sterna dark brown to black, covered with long whitish pile.

Male genitalia. Apical part of anterior surstyle lobe rhomboid in shape, ca. 1.5 times longer than wide, covered with dense, short pile (Fig. 18A: al); posterior surstyle lobe oval with basolateral protrusion (lateral hump) (Fig. 18A, B: bp); hypandrium sickle-shaped, without lateral projections; lingula medium sized (Fig. 18C: l).

**Female.** Similar to the male except for normal sexual dimorphism and for the following characteristics: basoflagellomere 1.6–1.8 times longer than wide, fossette large, dorsolateral (Fig. 12I); frons mostly microtrichose and predominantly covered with black pile; ocellar triangle covered with black pile; microtrichose fasciae on terga 3 and 4 conspicuous.

##### Etymology.

The name derives from the Latin adjective *disjunctus* meaning separated, disconnected which pertains to the dichoptic eyes in the males.

##### Distribution.

*Merodon disjunctus* sp. nov. has so far only been recorded in Kyrgyzstan and Kazakhstan (Fig. 7).

##### Ecology.

Preferred environment: no data. Flowers visited: no data. Flight period: May-July.

##### Type material.

**Holotype**. Kyrgyzstan • ♂; Talassky Mt.R, Ara Bijik rav., 13 km NNE Majdantal Pass; 42°22'00"N, 70°00'00"E; 2700 m a.s.l.; 4 Jul. 1988; Milko leg.; SZMN 05847. Original label: “HOLOTYPE of *Merodon* / *disjunctus* Vujić, Likov et / Radenković sp.n. 2019” [red label], “NW KIRG., Talassky Mt.R, / Ara Bijik rav., 13 km NNE / Majdantal Pass ~2700 m / 42°22’N 70°57’E / 04.07.1998 *D. Milko leg*.”, “05847” (See Supplementary file 2: Figure 2B). **Paratypes**. Kazakhstan • 1 ♂; Ketmen, Mt. Kirgyzsay; 43°16'60"N, 79°31'00"E; 2200 m a.s.l.; 3 Jun. 2001; M. Hauser leg.; M. H. coll. 02470 • 2 ♂♂; Ketmen, Mt. Kirgyzsay; 43°16'60"N, 79°31'00"E; 1800 m a.s.l.; 1–3 Jun. 2001; M. Hauser leg.; M. H. coll. 02467, 02472 • 1 ♀; Almaty, Charyn; 43°46'58"N, 79°23'24"E; 20 May 2003; A. Selin leg.; S. K. coll. 03971 • 2 ♀♀; Kyzyltchy; 46°03'31"N, 80°43'10"E; 21 May 2004; A. Selin leg.; S. K. coll. 03970, 02460 • 1 ♂; Kyzyltchy; 46°03'31"N, 80°43'10"E; 21 May 2004; A. Selin leg.; S. K. coll. 02461 • 1 ♂; Talasskip hr., R. Kara-Bura; 1600 m a.s.l.; 18 Jul. 1968; Pek leg.; SZMN 05815.

Kyrgyzstan • 1 ♂; Tchatkal Valley, 4 SW Ajgyr-Dzhal vill.; 41°42'00"N, 70°57'00"E; 1800 m a.s.l.; 12 Jul. 1998; Milko leg.; SZMN 05848 • 1 ♂; Issyk Kul, Chong Kemin-Tal; 42°40'60"N, 75°55'00"E; 1350 m a.s.l.; 3 Jun. 1998; M. Kraus M. leg.; NBCN 02468 • 1 ♀; Issyk Kul, Chong Kemin-Tal; 42°42'00"N, 75°54'00"E; 1350 m a.s.l.; 3 Jun. 1998; M. Kraus leg.; NBCN 02464 • 1 ♂; Issyk Kul, Chong Kemin-Tal; 42°42'00"N, 75°54'00"E; 1350 m a.s.l.; 3 Jun. 1998; M. Kraus leg.; NBCN 02469.

#### Merodon hirsutus

Taxon classificationAnimaliaDipteraSyrphidae

Sack, 1913

FD6AAB04-0A71-5819-93A2-A81A7DB4E044

**Figure 19.**
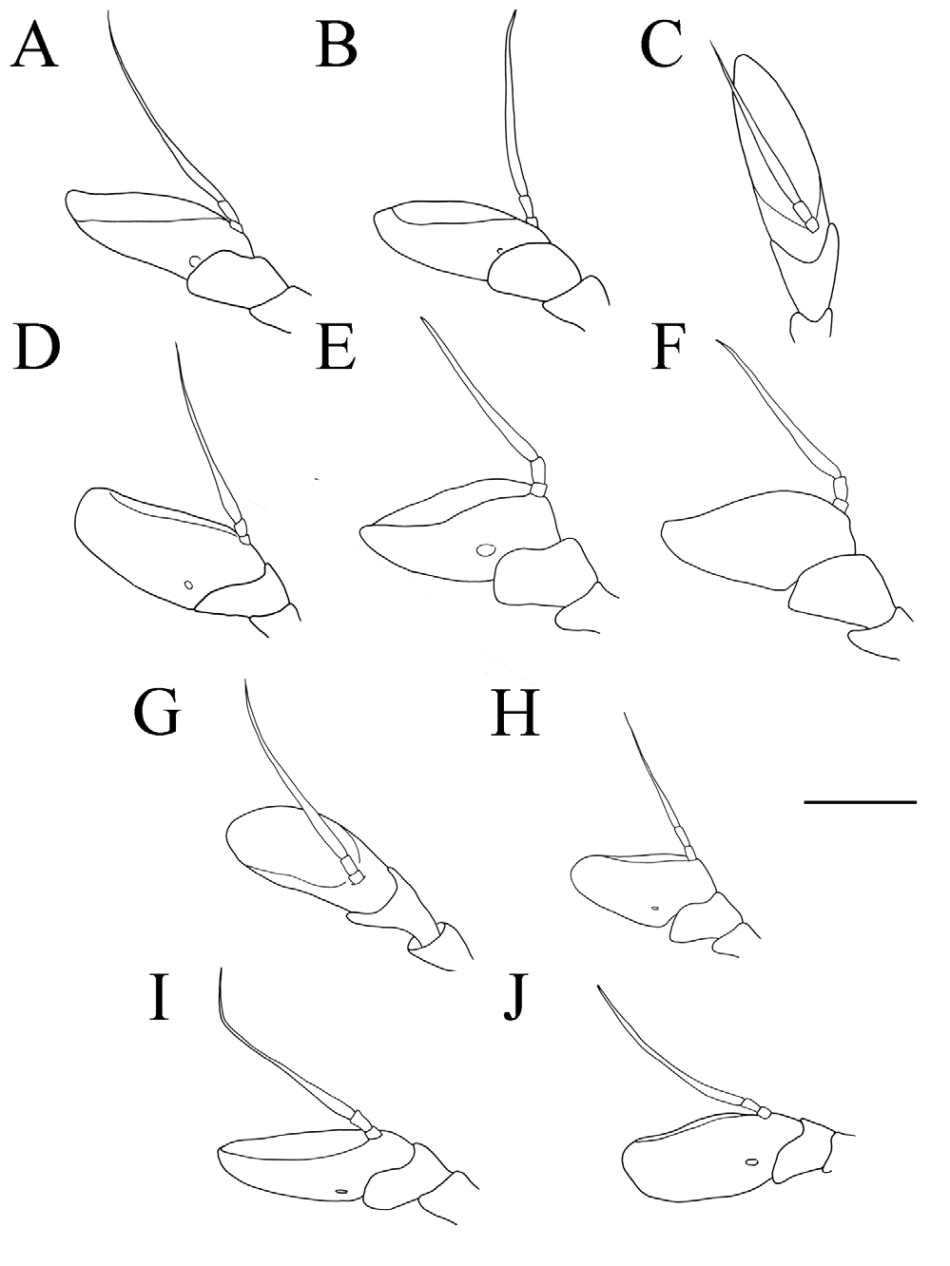
Antenna. **A***Merodon hirsutus*, outer side, lateral view, male **B***Merodon hirsutus*, inner side, lateral view, male **C***Merodon hirsutus*, dorsal view, male **D***Merodon hirsutus*, outer side, lateral view, female **E***Merodon kawamurae*, outer side, lateral view, male **F***Merodon kawamurae*, inner side, lateral view, male **G***Merodon kawamurae*, dorsal view, male **H***Merodon kawamurae*, outer side, lateral view, female **I***Merodon medium* sp. nov., outer side, lateral view, male **J***Merodon medium* sp. nov., outer side, lateral view, female. Scale bar: 1 mm.

**Figure 20.**
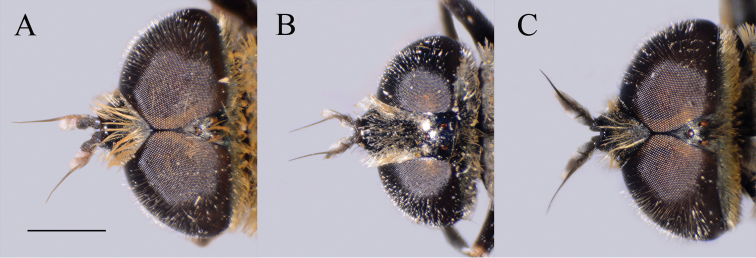
Head, dorsal view. **A***Merodon hirsutus*, male **B***Merodon hirsutus*, female **C***Merodon opacus* sp. nov., male. Scale bar: 2 mm.

**Figure 21.**
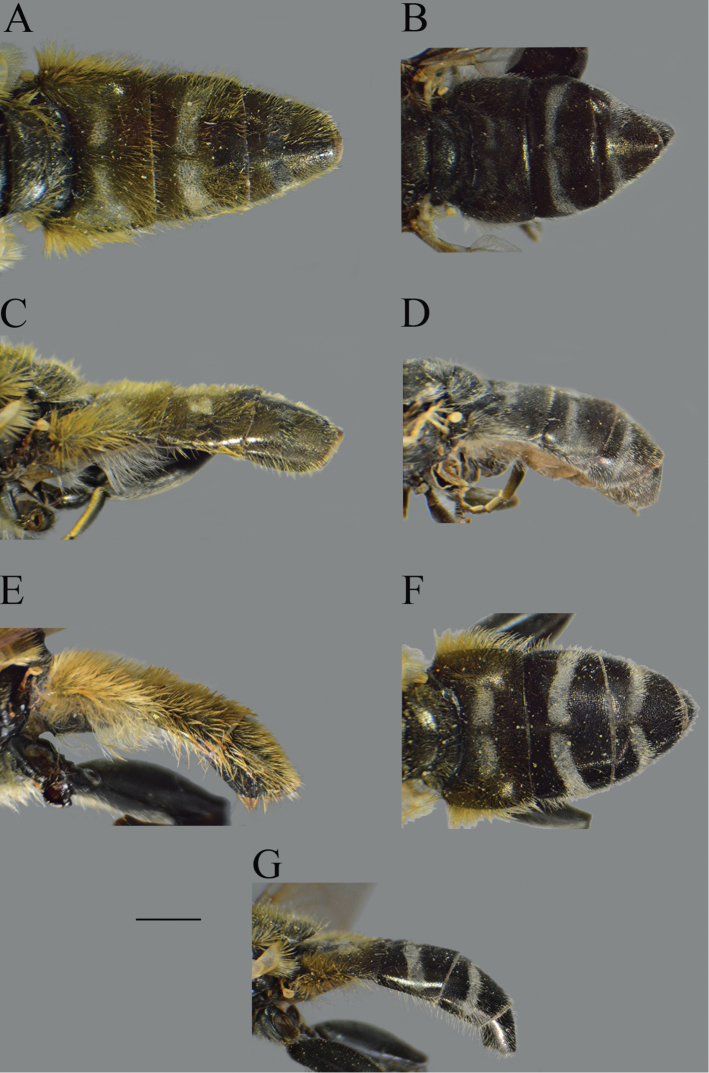
Abdomen. **A***Merodon opacus* sp. nov., dorsal view, male **B***Merodon opacus* sp. nov., dorsal view, female **C***Merodon opacus* sp. nov., lateral view, male **D***Merodon opacus* sp. nov., lateral view, female **E***Merodon hirsutus*, lateral view, male **F***Merodon hirsutus*, dorsal view, female **G***Merodon hirsutus*, lateral view, female. Scale bar: 2 mm.

Figs 13C, D , 14E–G , 19A–D , 20A, B , 21E–G

##### Diagnosis.

Medium sized (8.1–10.4 mm), dark species with olive-brown reflection; antennae dark brown; legs mostly black; basoflagellomere elongated (ca. two times as long as wide) obviously concave dorsally; arista 1.6–1.7 times as long as basoflagellomere (Fig. 19A–D); terga dark brown to black; metafemur incrassate, covered with short pilosity ventrally, and with long pile on dorsolateral surface (Fig. 13C); male genitalia: posterior surstyle lobe with lateral hump (Fig. 14E, F: bp); apical part of anterior surstyle lobe rhomboid (Fig. 14E: al); lingula large (Fig. 14G: l). Similar to *Merodon serrulatus* from which differs in dark tergum 2 (in *M. serrulatus* with small pale lateral maculae, at least in females). Morphologically related to *M. opacus* sp. nov. from which can be distinguished by longer dorsolateral and ventral pile on metafemur (Fig. 13C) and longer pile on terga (Fig. 21F, G); females with mostly shiny frons (Fig. 20B) (in *M. opacus* sp. nov. dull, covered with dense microtrichia).

##### Redescription

(based on lectotype and additional specimens from the type locality, Syria, and Israel). **Male.** Head. Antennae black to dark brown; basoflagellomere elongated ca. two times as long as wide, and 2.2–2.5 times as long as pedicel, concave dorsally with acute apex; large fossette dorsolateral and dorsomedial; arista dark and thickened at basal one third, covered with dense microtrichia, 1.6–1.7 times as long as basoflagellomere (Fig. 19A–C); face black with gray microtrichia, covered with whitish pile; frons mostly shiny, with yellowish gray pile; oral margin microtrichose, with small, shiny lateral area; lunule shiny black, bare; eye contiguity 12–14 facets long; vertex isosceles, with long, pale whitish yellow pile, mixed with black pile on the ocellar triangle; ocellar triangle equilateral; occiput with gray-yellow pile; eyes covered with dense pile (Fig. 20A); vertical triangle: eye contiguity: frons = 1.4 : 1 : 2 .

Thorax. Scutum and scutellum black with bronze luster, covered with dense, erect, yellow pile; scutum at wing basis with short black pile; scutum usually with two or more microtrichose vittae, anteriorly connected and posteriorly reaching the scutellum; scutum dull; posterodorsal part of anterior anepisternum, posterior anepisternum (except anteroventral angle), anterior anepimeron, dorsomedial anepimeron, and posterodorsal and anteroventral parts of katepisternum with long, pale yellow pile and grayish microtrichia; wings entirely covered with microtrichia; wing veins brown; calypteres and halteres yellowish; legs mostly black, except brown tarsi ventrally in some specimens; pile on legs pale yellow, except black pile at apical one third of metafemur; metafemur moderately incrassate, ca. three times longer than wide; pile on postero- and anteroventral surface very short; pile on dorsolateral surface long and dense ca. as half of width of metafemur (Fig. 13C).

Abdomen. Tapering, ca. 1.2 times longer than mesonotum; terga dark; terga 2–4 each with a pair of white microtrichose, wide, oblique fasciae (on tergum 2 triangular); pile on terga long, all yellow (Fig. 21F); sterna dark brown, covered with long whitish yellow pile.

Male genitalia. Apical part of anterior surstyle lobe rhomboid shape, ca. two times longer than wide, covered with short pile (Fig. 14E: al); posterior surstyle lobe oval with basolateral protrusion (lateral hump) (Fig. 14E, F: bp); hypandrium sickle-shaped, without lateral projections; lingula large (Fig. 14G: l).

**Female.** Similar to the male except for normal sexual dimorphism and for the following characteristics: antennae with rounded tip, basoflagellomere 1.8–2 times longer than wide, fossette dorsolateral (Fig. 19D); frons with narrow microtrichose vittae along eye margins; frons covered with variable pilosity, from mostly gray-yellow to predominantly black; ocellar triangle covered with black pile; metafemur with shorter pile on dorsolateral surface (Fig. 13D); terga mostly with yellowish pile on tergum 2 and with whitish pile on terga 3–5, except terga 2–4 medially with short black pile; microtrichose fasciae on terga 3 and 4 narrower (Fig. 21F, G).

##### Distribution.

*Merodon hirsutus* is distributed in Israel, Syria, and south-eastern Turkey (Fig. 7).

##### Ecology.

Preferred environment: no data. Flowers visited: no data. Flight period: March-June.

##### Type material.

Described by Sack (1913: 435) based on unspecified number of specimens. Lectotype [designated by Hurkmans (1993)]: male, “Jebel al Aqra [35° 35’ N, 36° 15’ E] vi.85 [1885], N. Syria, Dr. E. Leuthner / *Lampetia hirsuta* Sack det. Sack” (NHMW) (studied).

##### Other material.

Israel • 1 ♀; Hefa, “Ma`yan Zevi” [Ma Yan Zevi]; 32°34'00"N, 34°55'60"E; 17 Apr. 1980; TAUI 04162.

Syria • 1 ♂; Jebel al Aqra; 35°55'18"N, 35°57'51"E; Jun. 1985; D. F. Leuthner leg.; NHMW 02479.

Turkey • 1 ♂; İçel, İcel-Taşucu, Silifke; 36°22'17"N, 33°54'54"E; 300 m a.s.l.; 17 Mar. 1984; FSUNS 04161 • 1 ♀; Erdemli; 36°44'59"N, 34°11'51"E; 8 Jun. 2008; Skorpik leg.; MNHN 17917 • 3 ♀♀; Pozantı-Tekir; 37°31'05"N, 34°47'42"E; 6 Jun. 2008; M. Kafka leg.; M. B. coll. 17918 to 17920 • 1 ♀; Kahramanmaraş, Andırın, Beyoluğu village; 37°45'00"N, 36°17'00"E; 1400 m a.s.l.; 7 Jun. 2002; S. Sarıbıyık leg.; S. S. coll. 02482 • 3 ♂♂; same data as for preceding; S. S. coll. 17910 to 17912 • 2 ♀♀; Kahramanmaraş, Andırın, Çiğşar village; 37°45'00"N 36°18'00"E; 1400 m a.s.l.; 7 Jun. 2002; S. Sarıbıyık leg.; S. S. coll. 17914, 17916 • 2 ♂♂; same data as for preceding; S. S. coll. 17913, 17915.

#### Merodon kawamurae

Taxon classificationAnimaliaDipteraSyrphidae

Matsumura, 1916

0BC7AEE0-0F75-51D6-AD13-D96DA7C18F97

**Figure 22.**
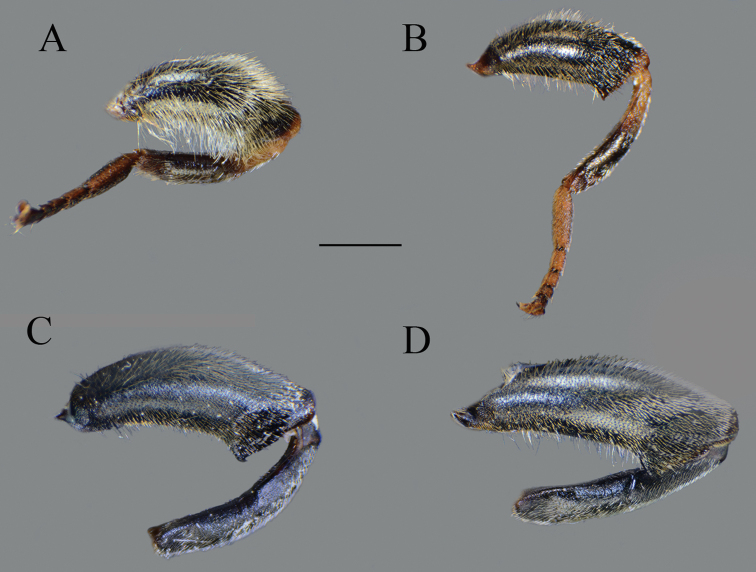
**A, B** metaleg, **C, D** metatrochanter, metafemur and metatibia, lateral view. **A***Merodon kawamurae*, male **B***Merodon kawamurae*, female **C***Merodon medium* sp. nov., male **D***Merodon medium* sp. nov., female. Scale bar: 2 mm.

**Figure 23.**
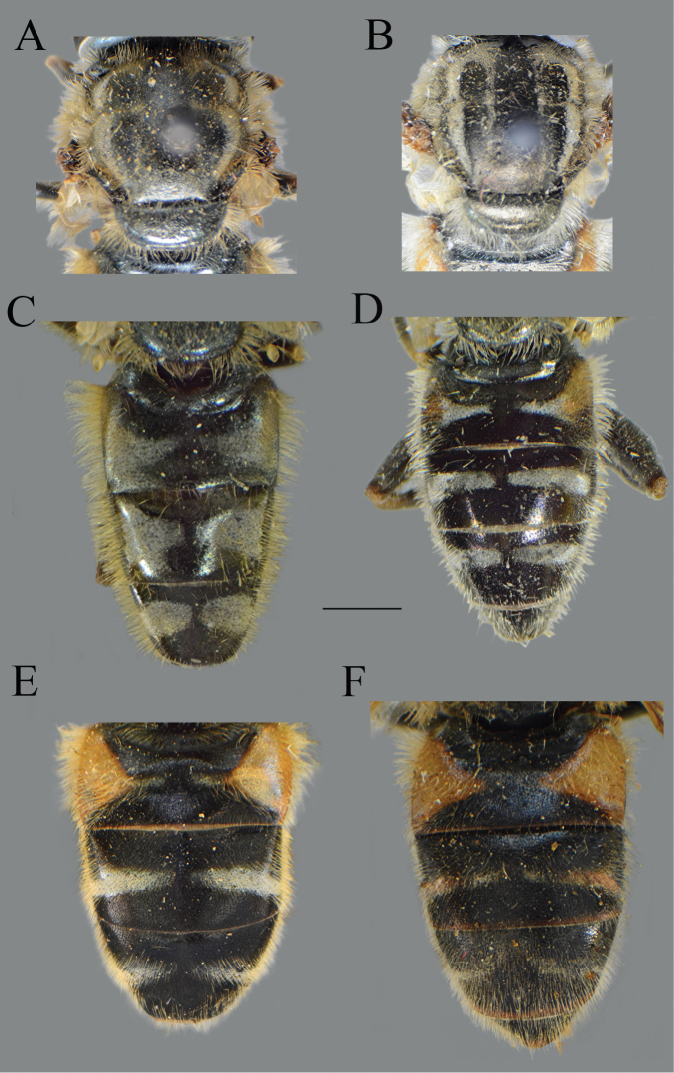
Body parts, dorsal view. **A***Merodon trianguloculus* sp. nov., thorax, male **B***Merodon trianguloculus* sp. nov., thorax, female **C***Merodon trianguloculus* sp. nov., abdomen, male **D***Merodon trianguloculus* sp. nov., abdomen, female **E***Merodon kawamurae*, abdomen, male **F***Merodon kawamurae*, abdomen, female. Scale bar: 2 mm.

Figs 18D–F , 19E–H , 22A, B , 23E, F

Lampetia micromegas Hervé-Basin, 1929: 111 – syn. published by Hurkmans 1993: 165.

##### Diagnosis.

Medium sized (7.7–11.2 mm), with olive-brown reflection; antennae reddish brown; body pile predominantly pale, except some black pile on vertex and terga 2–4 medially; basoflagellomere short, ca. 1.2 times as long as wide, with large dorsal to dorsolateral fossette, and short arista (Fig. 19E–H); tergum 2 with reddish yellow lateral maculae; tergum 3 laterally reddish or brown; metafemur incrassate with long pilosity as long as half of width of metafemur in male and as one third of width of metafemur in female (Fig. 22A, B); male genitalia: posterior surstyle lobe with small lateral hump (Fig. 18E: bp); apical part of anterior surstyle lobe rhomboid (Fig. 18D: al); lingula large (Fig. 18F: l), lateral sclerite of aedeagus elongated (Fig. 18F: s).

##### Redescription

(based on the types of *Merodon micromegas* and additional material from China). **Male.** Head. Antennae reddish brown; basoflagellomere short, ca. 1.2 times as long as wide, and ca. two times as long as pedicel, straight dorsally with acute apex; dorsal to dorsolateral fossette large; arista reddish brown and thickened at basal one third, covered with dense microtrichia, ca. 1.3 times as long as basoflagellomere (Fig. 19E–G); face and frons black with gray microtrichia, face covered with dense whitish, and frons with yellowish white pile; lunule shiny black, bare; vertex isosceles, dull, in front of anterior ocellus covered with dense microtrichia; vertex with long, pale yellow pile, in some specimens mixed with black or dark gray pile on the ocellar triangle; ocellar triangle isosceles; eyes covered with dense pile; occiput with gray-yellow pile, ventrally covered with a dense, gray microtrichia; eye contiguity ca. ten facets long; vertical triangle: eye contiguity: frons = 2.5 : 1 : 2.5.

Thorax. Scutum and scutellum black with bronze luster, covered with dense, erect yellow pile; scutum usually with indistinct microtrichose vittae; posterodorsal part of anterior anepisternum, posterior anepisternum (except anteroventral angle), anterior anepimeron, dorsomedial anepimeron, and posterodorsal and anteroventral parts of katepisternum with long, dense pale yellow pile and grayish microtrichia; wings entirely covered with microtrichia; wing veins reddish brown; calypteres and halteres pale yellow; legs mostly black, except tip of femora and basal part of tibiae and brown tarsi ventrally; pile on legs pale yellow; metafemur incrassate and curved, ca. three times longer than wide; long pile on postero- and anteroventral surface ca. as half of width of metafemur, approximately the same length as pile on dorsal surface (Fig. 22A).

Abdomen. Broad, tapering, 1.2 times longer than mesonotum; terga dark, except for a pair of reddish yellow, triangular, lateral maculae on tergum 2 (and in some specimen on 3); terga 2–4 each with a pair of white microtrichose, wide, usually oblique fasciae; pile on terga all yellow, except black pile on tergum 3 medially, and on tergum 2 posteriorly and tergum 4 anteriorly in some specimens (Fig. 23E); sterna dark brown, covered with long whitish yellow pile.

Male genitalia. Apical part of anterior surstyle lobe rhomboid shape, covered with dense, short pile (Fig. 18D: al); posterior surstyle lobe oval with small basolateral protrusion (lateral hump) (Fig. 18E: bp); hypandrium sickle-shaped, without lateral projections; lingula large (Fig. 18F: l); lateral sclerite of aedeagus elongated (Fig. 18F: s).

**Female.** Similar to the male except for normal sexual dimorphism and for the following characteristics: antennae with rounded tip, fossette dorsolateral (Fig. 19D); frons microtrichose, covered with mostly gray-yellow pile; ocellar triangle covered with black pile; long pile on postero- and anteroventral surface ca. as half of width of metafemur (Fig. 22B); microtrichose fasciae on terga 2–4 narrower (Fig. 23F).

##### Distribution.

*Merodon kawamurae* is known from Japan and China (Fig. 7). This is the only species of the genus *Merodon* in eastern Palaearctic.

##### Ecology.

Preferred environment: no data. Flowers visited: no data. Flight period: April-May.

##### Type material.

*Merodon kawamurae* was described after an unknown number of specimens from Kumamoto, Kyushu, Japan, leg. Kawamura. Matsumura’s type material is held at the Hokkaido University, Department for Systematic Entomology, at Sapporo, Japan, but the type material was inaccessible for this study.

*Merodon micromegas* Lectotype [designated by Hurkmans (1993)]: “Tchen-Kiang, 13.iv.1918 / *Lampetia micromegas* H. B. type” (MNHN) (studied).

**Paralectotypes** (*Lampetia micromegas*). CHINA • 1 ♂; Chemo; 33°44'32"N, 103°23'45"E; 25 Apr. 1918; MNHN 02520 • 1 ♂; Chemo; 33°44'32"N, 103°23'45"E; 26 Apr. 1918; MNHN 02521 • 1 ♀; Chemo; 33°44'32"N, 103°23'45"E; 23 Apr. 1918; MNHN 02522 • 1 ♀; Shia-Shu; 10 May 1918; MNHN 02525 • 1 ♀; Jiangsu, Nanking; 32°00'27"N, 118°57'22"E; 6 May 1918; MNHN 02524.

##### Other material.

CHINA • 1 ♀; Ningpo; 29°44'29"N, 121°06'02"E; 29 Apr. 1925; J. T. Chu leg.; NMNH 05118 • 1 ♂; Jiangsu, Nanking; 32°00'27"N, 118°57'22"E; 1981; H. Jettmar leg.; NHMW 02516 • 1 ♀; Jiangsu, Nanking; 32°00'27"N, 118°57'22"E; 15 Apr. 1918; NBCN 02518 • 1 ♀; same data as for preceding; 16 Apr. 1918; MNHN • 16 ♂♂; Chenkiang; 32°08'24"N, 119°23'25"E; 1–13 Apr. 1918; MNHN • 12 ♀♀; same data as for preceding; MNHN • 6 ♂♂; Chemo; 33°44'32"N, 103°23'45"E; 23 Apr. 1918; MNHN • 4 ♀♀; same data as for preceding; MNHN • 1 ♀; Chemo; 33°44'32"N, 103°23'45"E; 23 Apr. 1918; NBCN • 1 ♀; same data as for preceding; 26 Apr. 1918; NBCN • 1 ♂; Hoachan; 16 May 1918; MNHN • 2 ♀♀; same data as for preceding; MNHN • 3 ♂♂; Shia-Shu; 22 Apr. 1918; MNHN.

#### Merodon medium

Taxon classificationAnimaliaDipteraSyrphidae

Vujić, Likov & Radenković sp. nov.

C4180E85-D7E1-5724-9056-23B0878186A7

http://zoobank.org/E384B35E-E377-49B6-90CE-DEBDCBCEDDCB

**Figure 24.**
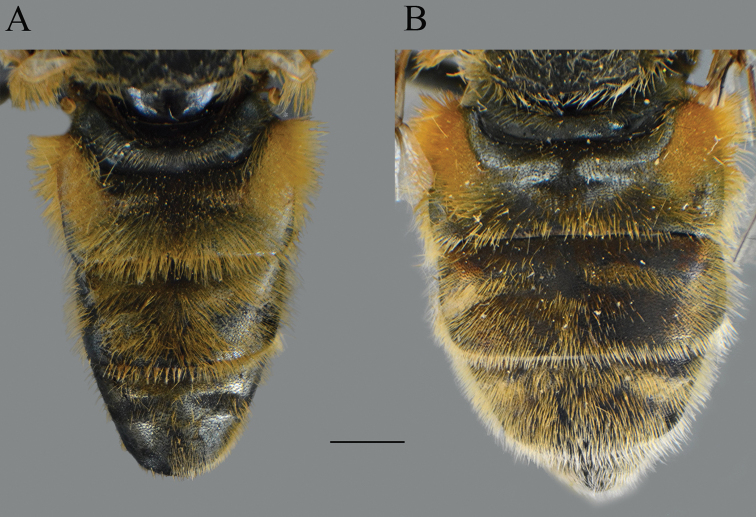
*Merodon medium* sp. nov., abdomen, dorsal view. **A** male **B** female. Scale bar: 2 mm.

**Figure 25.**
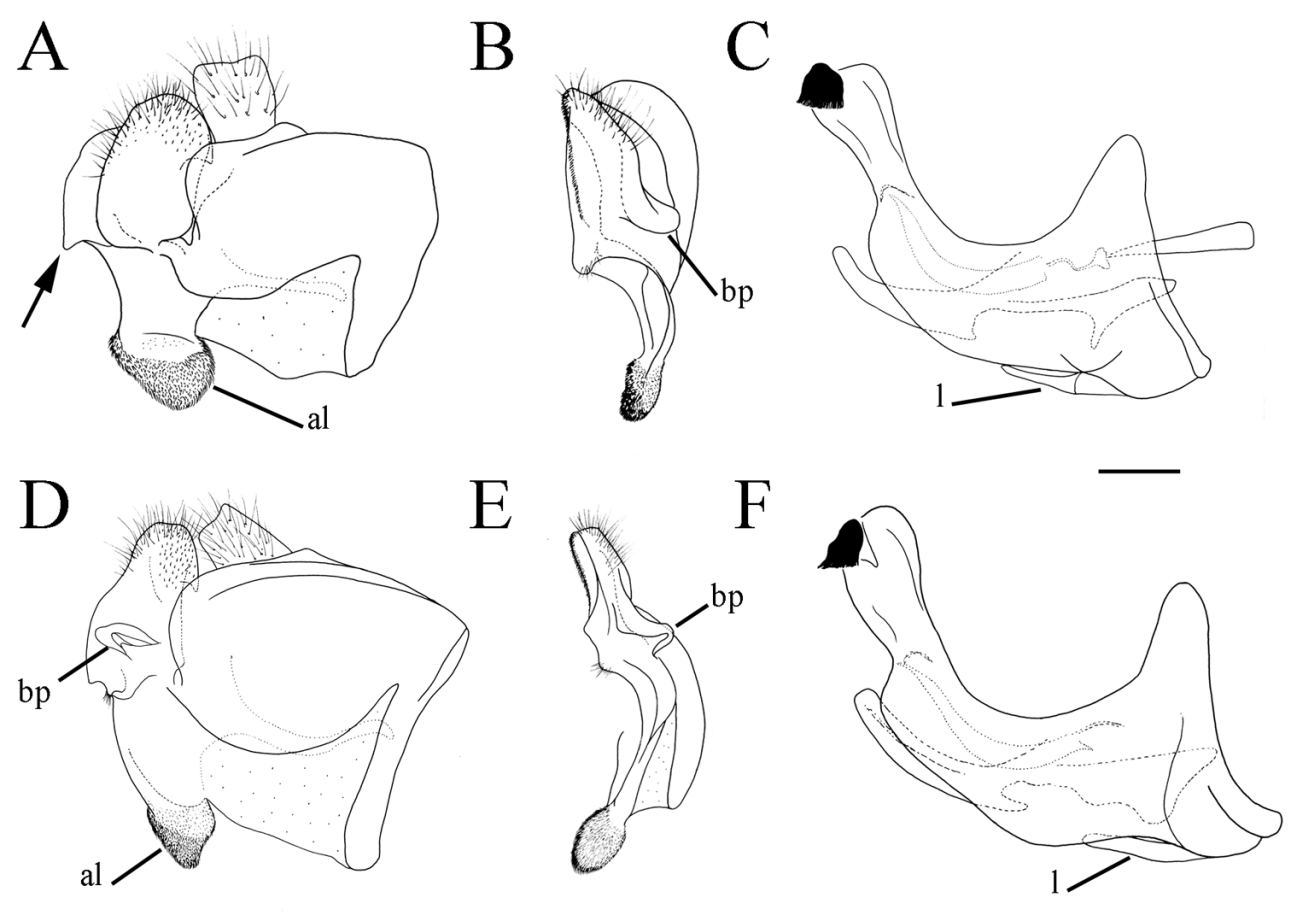
Male genitalia. **A***Merodon medium* sp. nov., epandrium, lateral view **B***Merodon medium* sp. nov., epandrium, ventral view **C***Merodon medium* sp. nov., hypandrium, lateral view **D***Merodon nigrocapillatus* sp. nov., epandrium, lateral view **E***Merodon nigrocapillatus* sp. nov., epandrium, ventral view **F***Merodon nigrocapillatus* sp. nov., hypandrium, lateral view. Abbreviations: al–anterior surstyle lobe, bp–basolateral protrusion, l–lingula; arrow marks the hook-like extension in **A**. Scale bar: 0.2 mm.

Figs 6H , 19I, J , 22C, D , 24A, B , 25A–C

##### Diagnosis.

Large species (10.3–13 mm) with wide dark brown abdomen and yellow-orange maculae on lateral sides of tergum 2 (Fig. 24A, B); basoflagellomere elongated, ca. 2.5 times longer than broad (Fig. 19 I, J,); metafemur incrassate (Fig. 22C, D); terga 2–4 with conspicuous microtrichose fasciae (Fig. 24A, B). Similar to some populations of *Merodon serrulatus*, but clearly differs in shape of abdomen: relation between maximum width of tergum 2 and its medial length is 3.3 in male and 3.5 in female of *M. medium* sp. nov. compared with 2.3 in *M. serrulatus* male and 2.7 in female; male genitalia: anterior surstyle lobe with concave margin in *M. medium* sp. nov. (Fig. 6H: marked with arrow), convex in *M. serrulatus* (Fig. 6G: marked with arrow); apical microtrichose area of anterior surstyle lobe 2.5 times broader than long in *M. medium* sp. nov. (Fig. 6G: al), less than one time in *M. serrulatus* (Fig. 6G: al); molecular data and distribution (*M. medium* sp. nov. is an endemic to the island of Crete in Greece).

##### Description.

**Male.** Head. Antennae black to dark brown; basoflagellomere elongated ca. 2.2 times as long as wide, and ca. 2.5 times as long as pedicel, concave dorsally with acute apex; dorsolateral fossette narrow; arista dark and thickened at basal one third, covered with dense microtrichia, ca. 1.5 times as long as basoflagellomere (Fig. 19I); face and frons black with gray microtrichia, face covered with dense whitish gray, and frons with yellowish gray pile; oral margin microtrichose with shiny lateral areas; lunule shiny black, bare; vertex shiny black, except microtrichose area in front of anterior ocellus; vertex isosceles, with long, pale whitish yellow pile, mixed with few black pile on the ocellar triangle; ocellar triangle equilateral; eyes covered with dense pile; occiput with gray-yellow pile, ventrally covered with a dense, gray microtrichia; eye contiguity 10–12 facets long; vertical triangle: eye contiguity: frons = 1.2 : 1 : 2.

Thorax. Scutum and scutellum black with bronze luster, covered with dense, erect, yellow pile, except sides of scutum at wing basis with patch of short black pile and fascia of black pile between wing basis; scutum with two or more microtrichose vittae, anteriorly connected and posteriorly reaching the scutellum; scutum dull; posterodorsal part of anterior anepisternum, posterior anepisternum (except anteroventral angle), anterior anepimeron, dorsomedial anepimeron, and posterodorsal and anteroventral parts of katepisternum with long, pale yellow pile and grayish microtrichia; wings entirely covered with microtrichia; wing veins brown; calypteres and halteres yellowish; legs mostly black, except brown tarsi ventrally in some specimens; pile on legs pale yellow, except few black pile in apical fifth of metafemur in some specimens; metafemur incrassate, ca. three times longer than wide; pile on postero- and anteroventral surface short, except few sparse pile approximately the same length as pile on dorsal surface (Fig. 22C).

Abdomen. Broad, tapering, 1.2 times longer than mesonotum; terga dark, except for a pair of yellow-orange, triangular, lateral maculae on tergum 2; terga 2–4 each with a pair of white microtrichose, oblique fasciae (on tergum 2 more triangular); pile on terga all yellow (Fig. 24A); sterna dark brown, covered with long whitish yellow pile.

Male genitalia. Apical part of anterior surstyle lobe rhomboid shape, 1.5 times longer than wide, covered with dense, short pile (Fig. 25A: al) and with concave margin (Fig. 6H: marked with arrow); posterior surstyle lobe oval with basolateral protrusion (lateral hump) (Fig. 25B: bp) and basal hook-like extension (Fig. 25A: marked with arrow); hypandrium sickle-shaped, without lateral projections; lingula medium sized (Fig. 25C: l).

**Female.** Similar to the male except for normal sexual dimorphism and for the following characteristics: antennae with rounded tip, basoflagellomere ca. two times longer than wide, (Fig. 19J); frons with broad microtrichose vittae along eye margins; frons covered with pilosity of variable color, from mostly gray-yellow until predominately black pile; ocellar triangle covered with black pile; metafemur incrassate, pile on postero- and anteroventral surface short (Fig. 22D); terga pale yellow pilose at lateral sides, anterior two thirds of tergum 2 and all terga 4 and 5; terga 2 and 3 medially with short adpressed black pile; microtrichose fasciae on terga 3 and 4 broad (Fig. 24B).

##### Etymology.

Medium (middle, center) refers to the species’ distribution, being the only taxon of the group found on Crete, in the middle of Mediterranean Sea.

##### Distribution.

*Merodon medium* sp. nov. is endemic to the Greek island of Crete (Fig. 7).

##### Ecology.

Preferred environment: forest/open ground; evergreen oak forest, dry *Pinus* forest; scrub with *Pistacia lentiscus* L.; well-vegetated, unimproved grassland. Flowers visited: *Ornithogalum* spp., *Potentilla* spp. and *Thymus* spp. Flight period: May.

##### Type material.

**Holotype**. Greece • ♂; Crete, Chania, Omalos plain; 35°19'21"N, 23°55'50"E; 28 May 2014; A. Vujić leg.; FSUNS 06729. Original label: “HOLOTYPE of *Merodon* / *medium* Vujić, Likov et / Radenković sp.n. 2019” [red label], “Greece, Crete, Chania, / Omalos plain / 28.05.2014. 35.322593 / 23.930496 Leg. Vujić”, “AU298”, “06729” (See Supplementary file 3: Figure 3). **Paratypes**. Greece • 1 ♀; Crete, Chania, Imbors; 35°15'08"N, 24°10'28"E; 27 May 2014; A. Vujić leg.; FSUNS 06706 • 1 ♂; Crete, Chania, Omalos plain; 35°19'06"N, 23°54'51"E; 28 May 2014; A. Vujić leg.; FSUNS 06723 • 1 ♂; Crete, Chania, Omalos plain; 35°19'21"N, 23°55'50"E; 28 May 2014; A. Vujić leg.; FSUNS 06731 • 1 ♀, Crete, Chania, Mescla, 35°24'05"N 23°56'26"E; 28 May 2014; A. Vujić leg.; FSUNS 06718.

#### Merodon nigrocapillatus

Taxon classificationAnimaliaDipteraSyrphidae

Vujić, Likov & Radenković sp. nov.

3EE0F8A4-02FF-506E-B00A-1B05E7CB8D22

http://zoobank.org/09B368F3-6BCF-4CF1-95E1-8AC43C14B1D4

**Figure 26.**
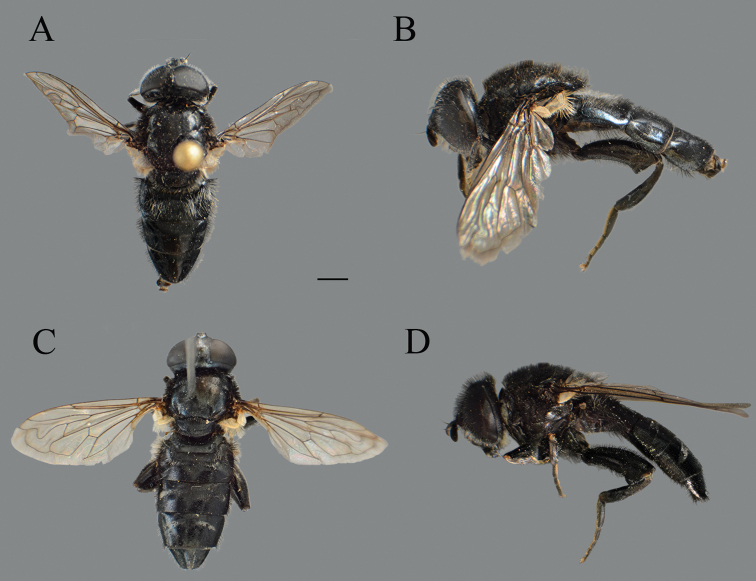
*Merodon nigrocapillatus* sp. nov., body parts. **A** dorsal view, male **B** lateral view, male **C** dorsal view, female **D** lateral view, female. Scale bar: 2 mm.

**Figure 27.**
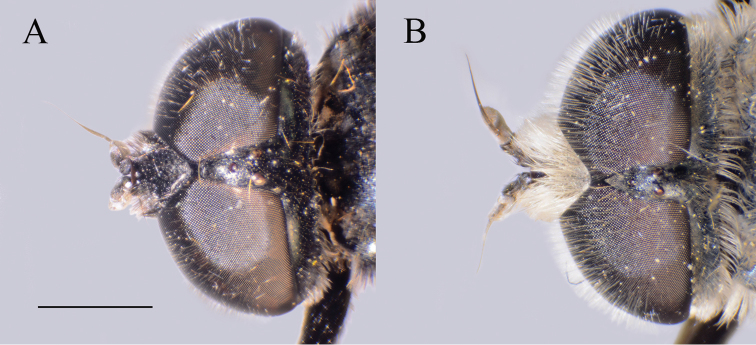
Head of male, dorsal view. **A***Merodon nigrocapillatus* sp. nov. **B***Merodon nigropunctum* sp. nov. Scale bar: 2 mm.

**Figure 28.**
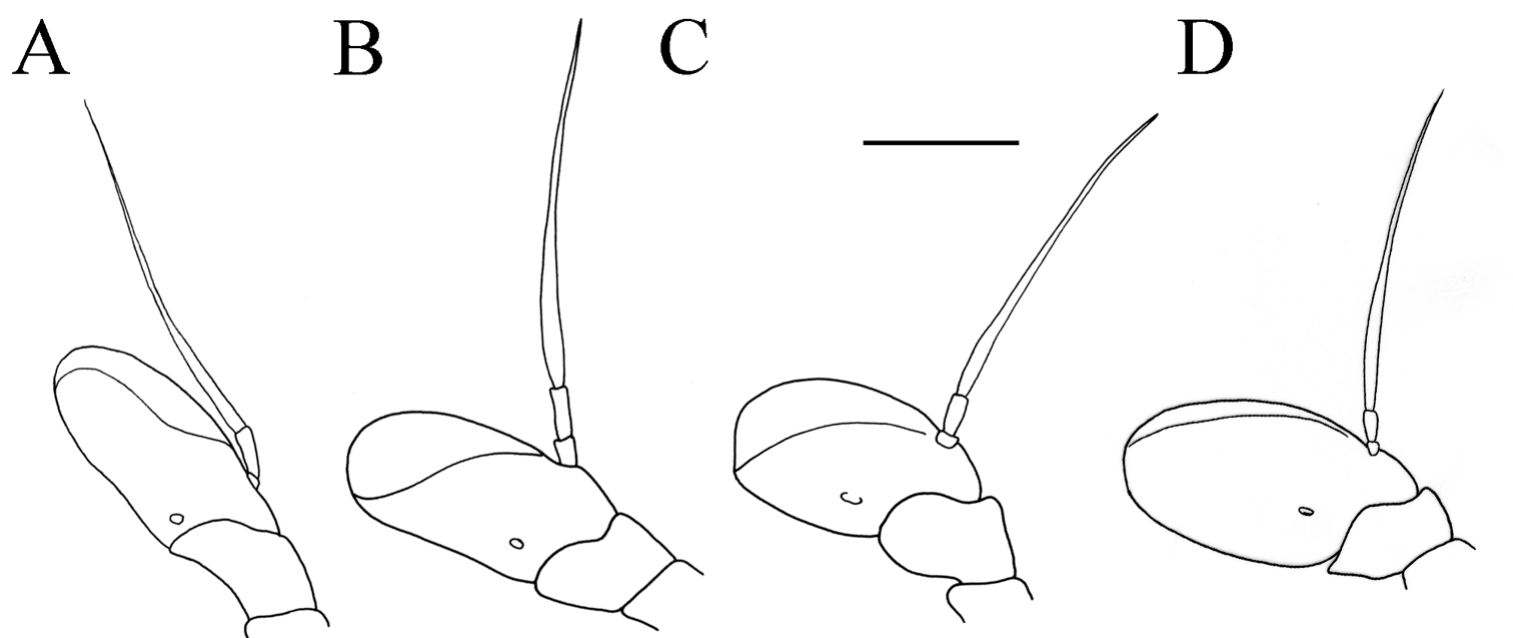
Antenna. **A***Merodon nigropunctum* sp. nov., outer side lateral view, male **B***Merodon nigropunctum* sp. nov., inner side, lateral view, male **C***Merodon nigrocapillatus* sp. nov., outer side, lateral view, male **D***Merodon nigrocapillatus* sp. nov., outer side, lateral view, female. Scale bar: 1 mm.

Figs 25D–F , 26A–D , 27A , 28C, D

##### Diagnosis.

Medium sized (10.2–10.8 mm), black and shiny species, covered with mostly black pile on scutum, terga and legs in both sexes (Fig. 26); antennae dark; legs black; male dichoptic (Fig. 27A); basoflagellomere short, 1.3 times longer than wide (Fig. 28C); terga black, without pale lateral maculae on tergum 2; microtrichose fasciae on terga 2–4 very narrow or absent in males (Fig. 26A) and narrow in females (Fig. 26C).

##### Description.

**Male.** Head. Antennae black; basoflagellomere short, 1.3 times as long as wide, and ca. two times as long as pedicel, with rounded apex; fossette dorsolateral; arista black and thickened at basal one third, covered with dense microtrichia, ca. two times as long as basoflagellomere (Fig. 28C); face and frons black with gray microtrichia, face covered with dense whitish gray or mixed black and whitish gray, and frons mostly with black pile; oral margin shiny, with lateral microtrichose area; lunule shiny black, bare; vertex isosceles, shiny black, except in front of anterior ocellus covered with microtrichia; vertex with long, black pile; ocellar triangle equilateral; eyes covered with dense whitish pile; occiput shiny covered with black pile in upper half, ventrally with gray-yellow pile and dense, gray microtrichia; eye dichoptic, separated by distance of three facets (Fig. 27A).

Thorax. Scutum and scutellum black, shiny, covered with dense, erect, black pile; scutum without microtrichose vittae (Fig. 26A); posterodorsal part of anterior anepisternum, posterior anepisternum (except anteroventral angle), anterior anepimeron, dorsomedial anepimeron, and posterodorsal and anteroventral parts of katepisternum with long, dark gray or black pile and grayish microtrichia; wings entirely covered with microtrichia, except bare area at basal one third; wing veins dark brown; calypteres gray; halteres blackish; legs black (Fig. 26B); pile on legs mostly black or dark gray; metafemur curved and medium incrassate, ca. four times longer than wide; pile on postero- and anteroventral surface ca. one third to half of width of metafemur, slightly shorter than pile dorsally.

Abdomen. Tapering, 1.2 times longer than mesonotum; terga completely dark; terga 3 and 4 without, or with indistinct pair of white microtrichose fasciae; pile on terga mostly black, except anteromedial part of tergum 2 partly covered with whitish pile (Fig. 26A); sterna dark brown to black, covered with long black and whitish pile.

Male genitalia. Apical part of anterior surstyle lobe rhomboid shape, ca. two times longer than wide, covered with dense, short pile (Fig. 25D: al); posterior surstyle lobe oval with basolateral protrusion (lateral hump) (Fig. 25D, E: bp); hypandrium sickle-shaped, without lateral projections; lingula large (Fig. 25F: l).

**Female.** Similar to the male except for normal sexual dimorphism and for the following characteristics: basoflagellomere 1.5 times longer than wide, fossette narrow (Fig. 28D); pile on face of variable color, from black to whitish; frons mostly microtrichose, covered with black pile; ocellar triangle covered with black pile; microtrichose fasciae on terga 2–4 narrow (Fig. 26C); thorax on lateral sides with variable pilosity color, from black to whitish.

##### Etymology.

The name *nigrocapillatu*s is derived from Latin adjective *niger* meaning black, dark and Latin noun *capillatus* meaning long-haired, referring to the long, black body pile of this species.

##### Distribution.

*Merodon nigrocapillatus* sp. nov. has only been recorded in Tajikistan (Fig. 7).

##### Ecology.

Preferred environment: open areas at high altitudes, unimproved grassland (Fig. 35D). Flowers visited: white Apiaceae. Flight period: June-July.

##### Type material.

**Holotype**. Tajikistan • ♂; Varzob, Kalon; 39°03'36"N, 68°52'12"E; 2440 m a.s.l.; 1–4 Jul. 2017; A. Barkalov leg.; SZMN 22625. Original label: “HOLOTYPE of *Merodon* / *nigrocapillatus* Vujić, Likov / et Radenković sp.n. 2019” [red label], “Таджикистан, Варзобское / ущ., 3 км с.-в. кишлака / Калон, 2440м н.у.м. 39,06° / N, 68,87° 1-4.07.2017 / Сб.А. Баркалов”, “*2017* / *sp. 1* / A. Barkalov det., 201” [label partly handwritten], “22625” (See Supplementary file 1: Figure 1B). **Paratypes**. Tajikistan • 1 ♀; Varzob, Kalon; 39°03'00"N, 68°52'48"E; 2484 m a.s.l.; 7–12 Jul. 2017; A. Barkalov leg.; SZMN 22626 • 1 ♀; 65km N of Dushanbe, S side ANZOP pass; 39°03'00"N, 68°19'12"E; 2380 m a.s.l.; 21 Jul. 2010; J. Dils, J. Faes leg.; G. V. W. coll. 10397 • 1 ♀; Iskanderkul kishlak, Sarytag; 39°03'00"N, 68°19'12"E; 2374 m a.s.l.; 14 Jun. 2018; A. Barkalov leg.; SZMN 24506 • 4 ♂♂; Varzob Canyon, 3 km N-E Kalon kishlak; 39°03'36"N, 68°52'12"E; 2440 m a.s.l.; A. Barkalov leg.; SZMN • 14 ♀♀; same data as for preceding; SZMN • 2 ♀♀; same data as for preceding; 4 and 7 Jul. 2017; V. Zinchenko leg.; SZMN • 3 ♀♀; same data as for preceding; 4 and 7 Jul. 2018; SZMN • 1 ♀; Varzob Canyon, 3 km N-E Kalon kishlak; 39°03'36"N, 68°52'12"E; 2356 m a.s.l.; 5 Jul. 2018; A. Barkalov leg.; SZMN 24502 • 1 ♀; same data as for preceding; 7 Jun. 2018; SZMN 24505 • 1 ♀; same data as for preceding; ~2400 m a.s.l.; 28 Jun. 2018; V. Zinchenko leg.; SZMN 24503 • 1 ♀; same data as for preceding; 30 Jun. 2018; SZMN 24504 • 1 ♀; Varzob Canyon, 4 km N-E Kalon kishlak; 39°04'48"N, 68°51'36"E; 3375 m a.s.l.; A. Barkalov leg.; SZMN • 1 ♀; Iskanderkul, Zmeinoe Lake; 39°05'20"N, 68°22'08"E; ~2217 m a.s.l.; 16 Jun. 2018; V. Zinchenko leg.; SZMN 24507.

#### Merodon nigropunctum

Taxon classificationAnimaliaDipteraSyrphidae

Vujić, Likov & Radenković sp. nov.

278701BD-9637-5CE7-886B-CFE372B8BEFD

http://zoobank.org/13F2CA9B-BD91-4FD6-B66A-B89DE0F13726

**Figure 29.**
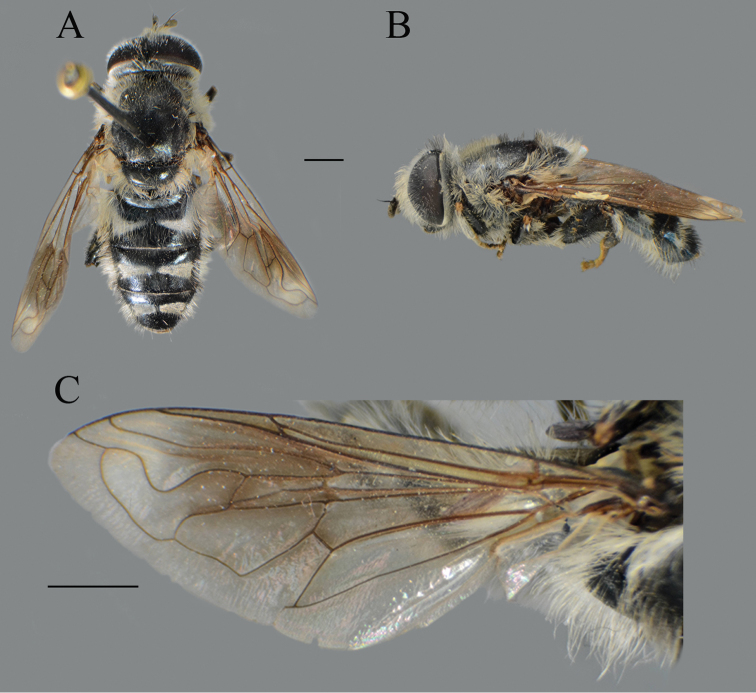
*Merodon nigropunctum* sp. nov., male. **A** body, dorsal view **B** body, lateral view **C** left wing, dorsal view. Scale bar: 2 mm.

**Figure 30.**
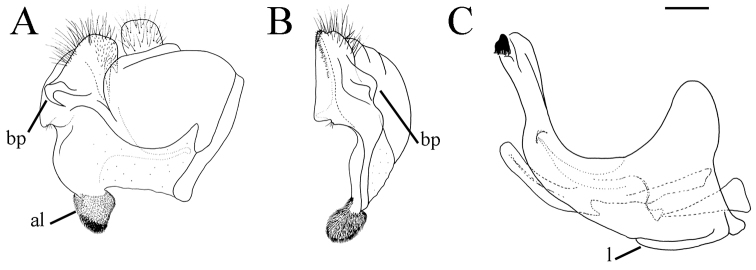
Male genitalia. **A***Merodon nigropunctum* sp. nov., epandrium, lateral view **B***Merodon nigropunctum* sp. nov., epandrium, ventral view **C***Merodon nigropunctum* sp. nov., hypandrium, lateral view. Abbreviations: al–anterior surstyle lobe, bp–basolateral protrusion, l–lingula. Scale bar: 0.2 mm.

Figs 27B , 28A, B , 29A–C , 30A–C

##### Diagnosis.

Medium sized (10.3 mm), bluish species (Fig. 29A, B) with dark macula on medial part of wing (Fig. 29C); basoflagellomere narrow and elongated, 1.8 times as long as wide, rounded at the tip (Fig. 28A, B); arista long, two times as long as basoflagellomere; body pile whitish; posterior half of scutum with square shaped area of black pile medially; terga 2–4 covered with black pile medially, except whitish pilosity on conspicuous silver microtrichose fasciae (Fig. 29A).

##### Description.

**Male.** Head. Antennae black to dark brown; basoflagellomere narrow and elongated, 1.8 times as long as wide, and 2.5 times as long as pedicel, with rounded tip; large fossette dorsomedial and dorsolateral (Fig. 28A, B); arista dark and thickened at basal one third, covered with dense microtrichia; arista long, ca. two times as long as basoflagellomere (Fig. 28A, B); face and frons black covered with whitish pile; face covered with indistinct whitish gray microtrichia; frons with dense whitish microtrichia; lunule shiny black, bare; vertex isosceles, with long whitish pile and black pilosity on the ocellar triangle; ocellar triangle equilateral; eyes covered with long, dense, whitish pile (Fig. 27B); occiput with whitish pile, covered with a dense, silver microtrichia along eye margin; eye contiguity short, approximately five facets long; vertical triangle: eye contiguity: frons = 4.5 : 1 : 4.5.

Thorax. Scutum and scutellum black with bluish luster, covered with dense, erect, white pile including wing basis; posterior half of scutum with square shaped area of black pile medially; scutum with indistinct microtrichose vittae; posterodorsal part of anterior anepisternum, posterior anepisternum (except anteroventral angle), anterior anepimeron, dorsomedial anepimeron, and posterodorsal and anteroventral parts of katepisternum with long, whitish pile and grayish microtrichia; wings entirely covered with microtrichia; wing veins dark brown; wing with distinct dark area in apical half (Fig. 29C); calypteres whitish; halteres yellowish, with darker capitulum; legs mostly black, except dark brown tarsi ventrally; pile on legs mostly whitish mixed with black ones on femora; metafemur moderately incrassate, ca. three times longer than wide; pile on postero- and anteroventral surface very long, and ca. two thirds of width of metafemur, approximately the same length as pile on dorsal surface.

Abdomen. Tapering, 1.2 times longer than mesonotum; terga dark brown to black; terga 2–4 each with a pair of white microtrichose, wide, oblique fasciae (on tergum 2 triangular); pile on terga long, whitish laterally and at microtrichose fasciae, medially black (Fig. 29A); sterna dark brown, covered with long whitish pile.

Male genitalia. Apical part of anterior surstyle lobe rhomboid shape, 1.5 times longer than wide, covered with dense, short pile (Fig. 30A: al); posterior surstyle lobe oval with basolateral protrusion (lateral hump) (Fig. 30A, B: bp); hypandrium sickle-shaped, without lateral projections; lingula large (Fig. 30C: l).

**Female.** Unknown.

##### Etymology.

The word *nigropunctum* is derived from the Latin words *niger* (black, dark whitish) and *punctum* (dot/spot) referring to the dark macula on the wing as an important diagnostic character of this new species.

##### Distribution.

*Merodon nigropunctum* sp. nov. was recorded at only one locality in Uzbekistan (Fig. 7).

##### Ecology.

Preferred environment: no data. Flowers visited: no data. Flight period: May.

##### Type material.

Holotype. Uzbekistan • ♂; Kadamžai, S of Fergana; 40°20'00"N, 71°47'39"E; 21 May 1980; Z. Padr leg.; NMPC 18248. Original label: “HOLOTYPE of *Merodon* / *nigropunctum* Vujić, Likov / Radenković sp.n. 2019” [red label], “C.ASIA, Uzbekistan / Kadanžai, S of Fergana / 21.5.1980 leg.Z.Pádr”, “18248” (See Supplementary file 4: Figure 4A).

#### Merodon opacus

Taxon classificationAnimaliaDipteraSyrphidae

Vujić, Likov & Radenković sp. nov.

5A6058B2-798E-52FA-9061-74D307F77217

http://zoobank.org/256F1010-6AAF-406A-830B-4F4A1D95126A

**Figure 31.**
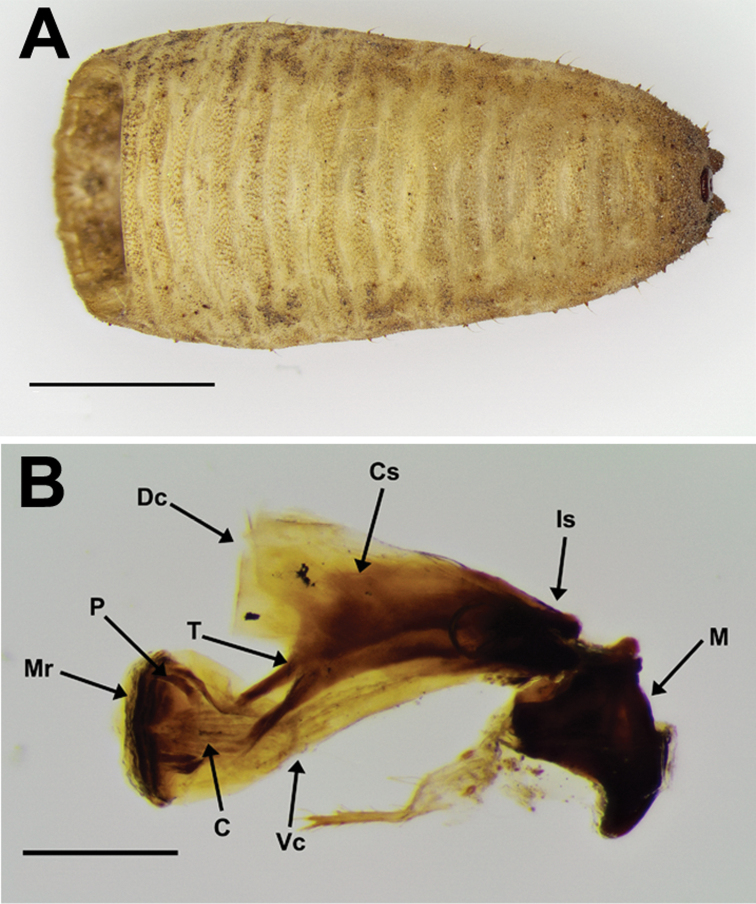
Light micrographs of *Merodon opacus* sp. nov. puparium. **A** puparium in dorsal view **B** cephalopharyngeal skeleton in lateral view. Abbreviations: C–cibarium, Cs–clypeal sclerite, Dc–dorsal cornu, Is–intermediate sclerite, M–mandibles, Mr–mortar, P–pestle, T–tentorium, Vc–ventral cornu. Scale bars: 3 mm (**A**); 500 μm (**B**).

**Figure 32.**
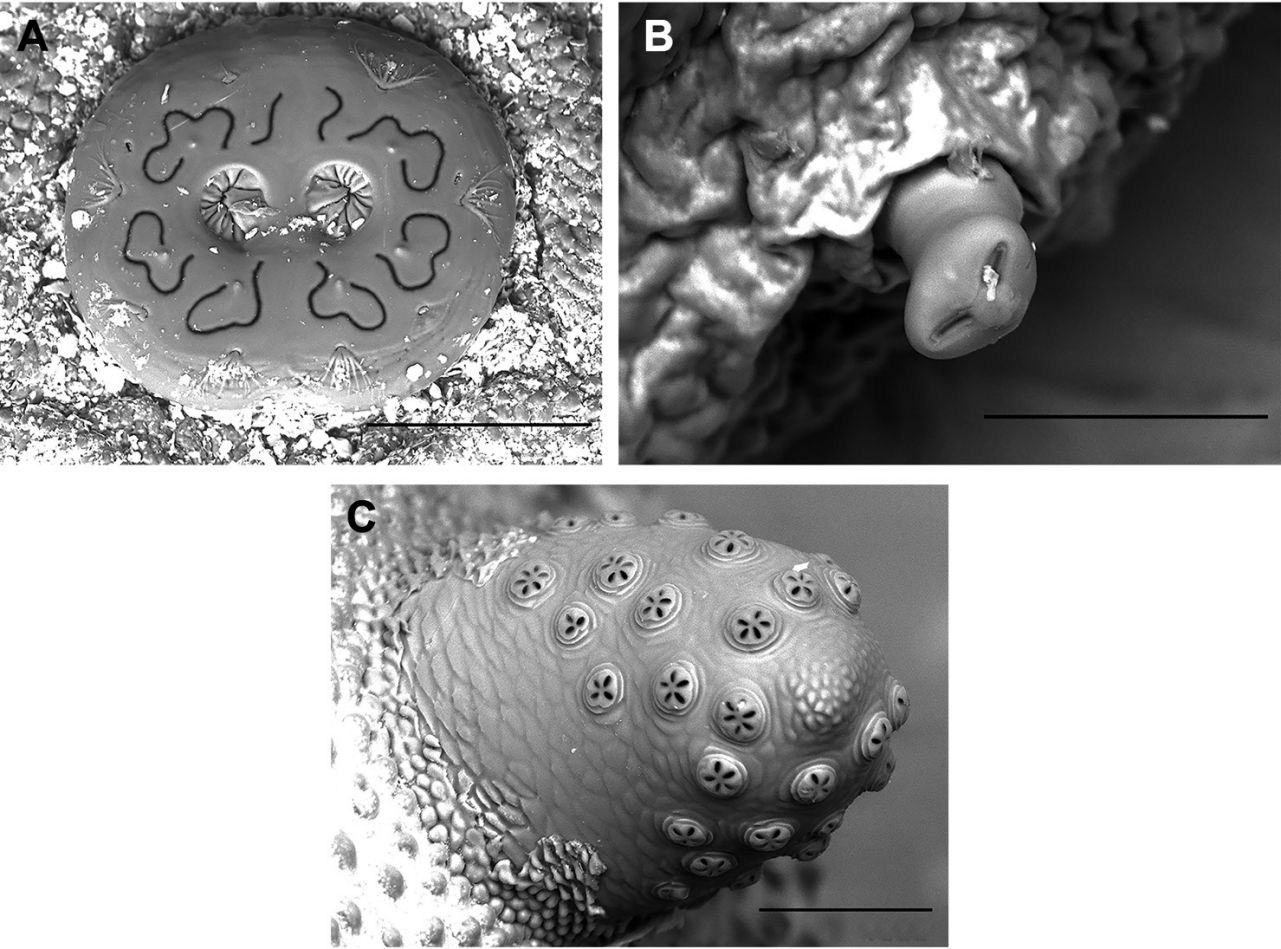
SEM micrographs of *Merodon opacus* sp. nov. puparium. **A** posterior respiratory process in polar view showing the spiracular plate **B** anterior spiracle **C** pupal spiracle. Scale bars: 200 μm (**A**); 100 μm (**B**, **C**).

Figs 12A–F , 13A, B , 14H–J , 20C , 21A–D , 31A, B , 32A–C

##### Diagnosis.

Medium sized (7.2–10.6 mm), short pilose dark species with olive-brown reflection; antennae dark; legs mostly black; basoflagellomere elongated (1.8–2 times as long as wide) obviously concave dorsally; arista short 1.5 times as long as basoflagellomere (Fig. 12A–F); terga dark (Fig. 21A, B); metafemur incrassate covered with very short pilosity (Fig. 13A, B); male genitalia: posterior surstyle lobe with small lateral hump (Fig. 14H, I: bp); apical part of anterior surstyle lobe rhomboid (Fig. 14H: al); lingula medium sized (Fig. 14J: l). Similar to *Merodon serrulatus* from which it differs in dark tergum 2 (in *M. serrulatus* with small pale lateral maculae). Related to *M. hirsutus* from which can be distinguished by frons covered with dense microtrichia (Fig. 20C) (mostly shiny in *M. hirsutus*), shorter dorsolateral and ventral pile on metafemur (Fig. 13A, B), and shorter and more adpressed pile on terga in females (Fig. 21D). Morphologically related to *M. defectus* sp. nov. from which can be distinguished by dark tergum 2 (in *M. defectus* sp. nov. tergum 2 with yellow-orange lateral maculae). Additionally, differs from *M. defectus* sp. nov. by posterior surstyle lobe with developed lateral hump (Fig. 14I: bp), reduced in *M. defectus* sp. nov. (Fig. 14B: bp).

##### Description.

**Male.** Head. Antennae black to dark brown; basoflagellomere elongated 1.8–2 times as long as wide, and 2.3 times as long as pedicel, concave dorsally with acute apex; large fossette dorsomedial and dorsolateral (Fig. 12A–C); arista dark and thickened at basal one third, covered with dense microtrichia, 1.5 times as long as basoflagellomere; face and frons black with gray microtrichia, face covered with dense whitish gray, and frons with yellowish gray pile; oral margin microtrichose with shiny lateral areas; lunule shiny black, bare; vertex covered with microtrichia (Fig. 20C); vertex isosceles, with long, pale whitish yellow pile, in some cases mixed with few black pile on the ocellar triangle; ocellar triangle equilateral; eyes covered with dense pile; occiput with gray-yellow pile, covered with a dense, gray microtrichia; eye contiguity ca. 10–12 facets long; vertical triangle: eye contiguity: frons = 1.2 : 1 : 2.

Thorax. Scutum and scutellum black with bronze luster, covered with dense, erect, yellow pile; scutum at wing basis with short black pile; scutum with two or more microtrichose vittae, anteriorly connected and posteriorly reaching the scutellum; scutum dull; posterodorsal part of anterior anepisternum, posterior anepisternum (except anteroventral angle), anterior anepimeron, dorsomedial anepimeron, and posterodorsal and anteroventral parts of katepisternum with long, pale yellow pile and grayish microtrichia; wings entirely covered with microtrichia; wing veins brown; calypteres yellowish; halteres yellowish, in some specimens with darker capitulum; legs mostly black, except brown tarsi ventrally in some specimens; pile on legs pale yellow; metafemur moderately incrassate, 3.5 times longer than wide; pile on postero- and anteroventral surface very short with few sparse pile, and ca. as one fourth of width of metafemur, approximately the same length as pile on dorsal surface (Fig. 13A).

Abdomen. Tapering, 1.2 times longer than mesonotum; terga dark brown to black; terga 2–4 each with a pair of white microtrichose, wide, oblique fasciae (on tergum 2 more triangular); pile on terga all yellow (Fig. 21A, C); sterna dark brown, covered with long whitish yellow pile.

Male genitalia. Apical part of anterior surstyle lobe rhomboid in shape, 1.5 times longer than wide, covered with dense, short pile (Fig. 14H: al); posterior surstyle lobe oval with small basolateral protrusion (lateral hump) (Fig. 14H, I: bp); hypandrium sickle-shaped, without lateral projections; lingula medium sized (Fig. 14J: l).

**Female.** Similar to the male except for normal sexual dimorphism and for the following characteristics: antennae with rounded tip, basoflagellomere ca. two times longer than wide, fossette dorsal (Fig. 12D–F); frons with broad microtrichose vittae along eye margins; frons covered with pilosity of variable color, from mostly gray-yellow to predominantly black; ocellar triangle covered with black pile; terga pale pilose, in some specimens terga 2–4 medially with short adpressed black pile; microtrichose fasciae on terga 3 and 4 narrower (Fig. 21B, D).

##### Morphological description of the puparium

(Fig. 31A). Length: 7.5 mm, width: 4 mm; light brown in color; sub-cylindrical; rough integument with larval segmentation persisting as transverse folds and wrinkles; integument covered with small domes and spicules; pronounced segmental sensilla, bearing seta. The dorsal surface of the prothorax with a pair of anterior spiracles, which are more than two times longer than broad at the base, sclerotized, cylindrical in shape, brown in color, apex with two linear spiracular openings (Fig. 32B). On the anal segment, two different pairs of lappets present: the ventro-lateral pair represented by fleshy papilla with one sensilla bearing a seta and the dorso-lateral pair with a very poorly developed basal papilla, apically divided bearing one sensilla with a long seta on top of each division. ***Cephalopharyngeal skeleton*** (Fig. 31B). Robust mandibles with dark highly sclerotized hooks, without accessory teeth, fused to the external mandibular lobes; the dorsal cornu narrowed, representing almost the whole length of the ventral cornu. Clypeal sclerite sclerotized, tentorium and intermediate sclerites highly sclerotized and apparently fused; ventral cornu elongated and narrow in profile view, wider and more heavily sclerotized at the posterior end, forming the grinding mill of pestle and mortar construction at the posterior end of the cibarium; cibarium at the base, with a clearly sclerotized end. ***Posterior respiratory process*** (Fig. 32A). Brownish, wider than long, very short (barely visible from dorsal view), button shape, base only slightly wider than apex; dorsal and lateral surface covered by a barely visible ornamentation resembling a network. The outline of the spiracular plate sub-elliptical and barely irregular. Spiracular plate with four pairs of sinuous spiracular openings (clearly separated from each other) around two central scars, first pair clearly shorter than the others; three or four very small circular nodules on each side of the surface of spiracular plate in the area of spiracular openings; four pairs of branched inter-spiracular setae emerging on the outward edges of the spiracular plate. ***Pupal spiracles*** (Fig. 32C). Sclerotized, brownish in color, stout, cylindrical in shape, almost as long as broad, slightly tapered, with not heavily rounded prominence at the end (length 0.3 mm) separated by a distance of ca. five times their length. Upper two-thirds of the lateral sides (except for the granularly surfaced apex) covered with irregularly-spaced, oval-shaped domed tubercles, leaving a more or less triangular central area free of tubercles on both ventral and dorsal surfaces; 3–7 radially-arranged spiracular openings on each tubercle. The whole spiracle surface (from the base to the apex) reticulated with a polygonal pattern, more irregular on ventral side, with polygons being noticeably smaller in the apical part. ***Material examined*.** Greece, Lesvos Island, Agiassos, C. Pérez-Bañón leg.: 1 larva (L3 instar) buried in the ground of a chestnut forest, 2 Mar 2006; reared, pupa 4 Mar 2006, adult emerged 21 Mar 2006.

**Figure 33.**
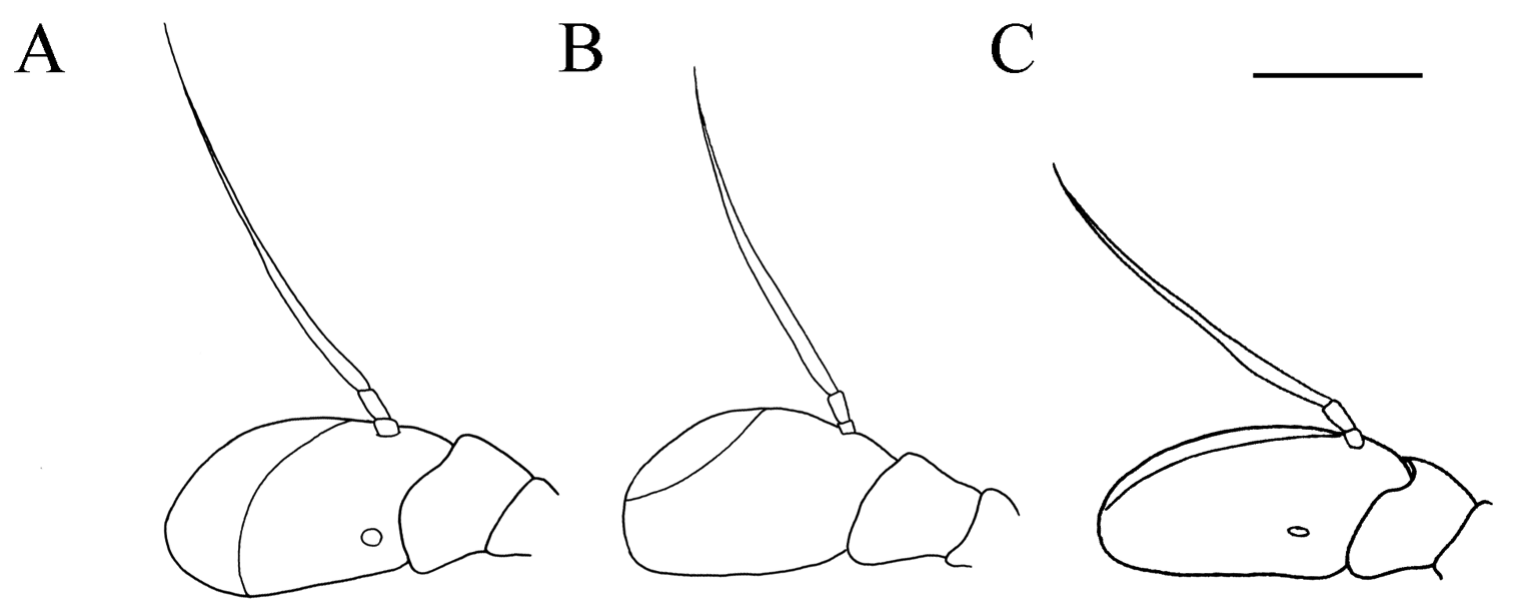
*Merodon trianguloculus* sp. nov., antenna, lateral view. **A** outer side, male **B** inner side, male outer side, female. Scale bar: 1 mm.

**Figure 34.**
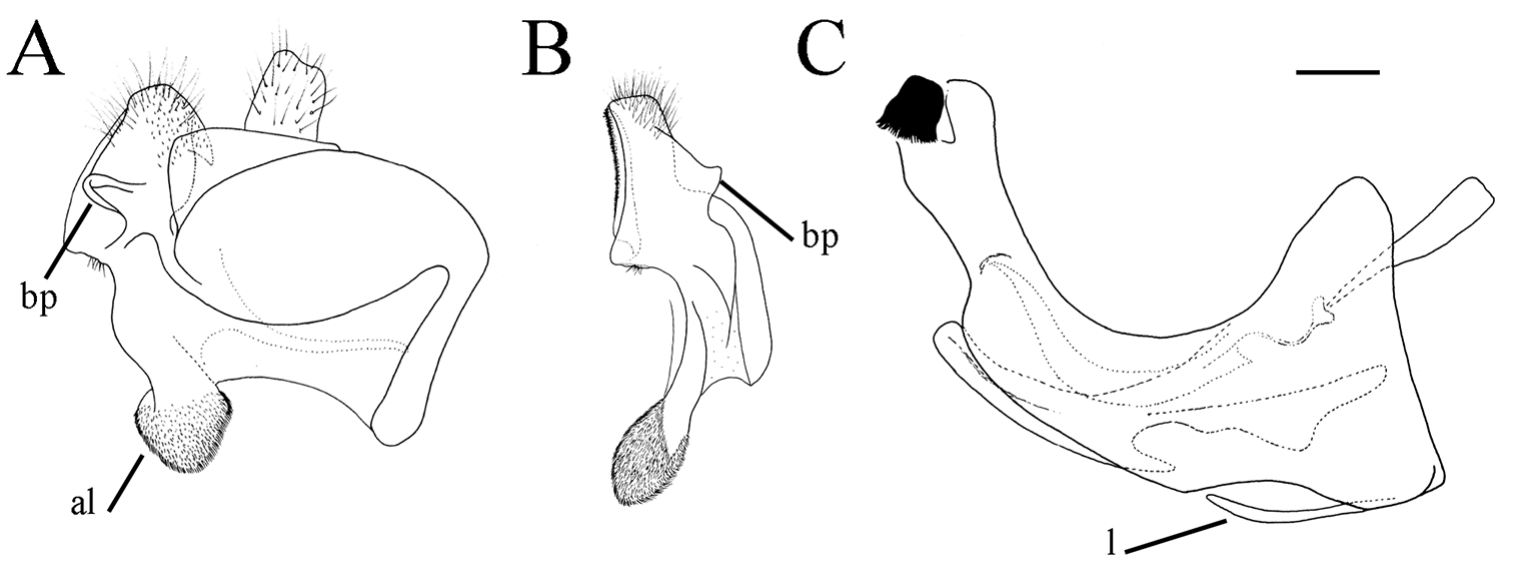
Male genitalia. **A***Merodon trianguloculus* sp. nov., epandrium, lateral view **B***Merodon trianguloculus* sp. nov., epandrium, ventral view **C***Merodon trianguloculus* sp. nov., hypandrium, lateral view. Abbreviations: al–anterior surstyle lobe, bp–basolateral protrusion, l–lingula. Scale bar: 0.2 mm.

##### Etymology.

Latin adjective *opacus* (opaque, not transparent), pertains to the dark tergum 2, without reddish yellow lateral maculae.

##### Distribution.

*Merodon opacus* sp. nov. has been recorded on the Greek island of Lesvos and in western Turkey (Fig. 7).

##### Ecology.

**Figure 35.**
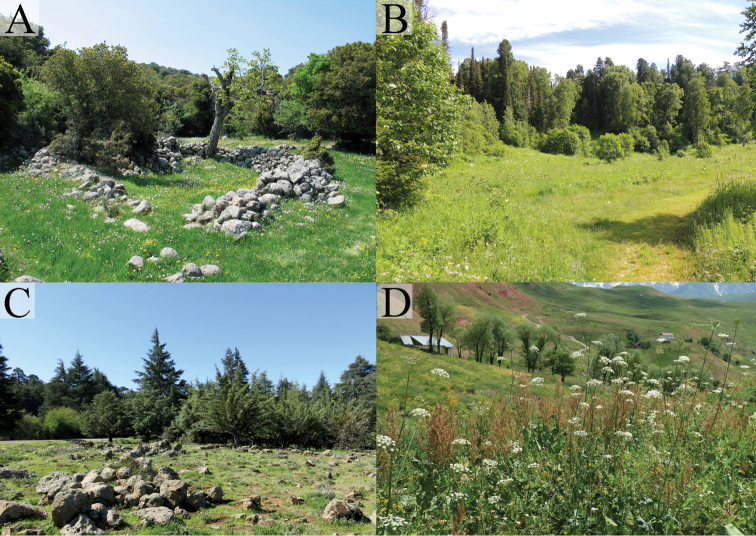
Different types of habitats of *Merodon serrulatus* species group. **A** Lesvos (Greece), habitat of *Merodon opacus* sp. nov., Photograph by Ante Vujić **B** Siberia, Teletskoye Lake (Russia), habitat of *Merodon serrulatus*, Photograph by Jeroen van Steenis **C** Morocco, habitat of *Merodon sophron*, Photograph by Ante Vujić **D** Tajikistan, habitat of *Merodon nigrocapillatus* sp. nov., Photograph by Anatolij Barkalov.

Preferred environment: forest/open ground; thermophilous and evergreen *Quercus* forest; *Castanea* forest, dry *Pinus* forest; unimproved grassland and tracksides (Fig. 35A). Flowers visited: *Ornithogalum* spp. and *Potentilla* spp. Flight period: March-September.

##### Type material.

**Holotype**. Greece • ♂; Lesvos; Polichnitos; 39°05'02"N, 26°09'13"E; 30 Apr. 2008; A. Vujić leg.; FSUNS 03758. Original label: “HOLOTYPE of *Merodon* / *opacus* Vujić, Likov et / Radenković sp.n. 2019” [red label], “Greece, Lesvos, / Polichnitos 30.IV 2008. / Leg. A. Vujic”, “03758” (See Supplementary file 4: Figure 4B). **Paratypes**. Greece, Lesvos • 1 ♂; Ag. Ermogenis; 39°01'07"N, 26°32'44"E; 2 May 2008; A. Vujić leg.; FSUNS 03760 • 1 ♂; Neochori II; 39°01'10"N, 26°20'02"E; 2 May 2016; A. Vujić, J. Ačanski leg.; FSUNS 11396 • 1 ♀; Agiassos; 39°03'00"N, 26°22'60"E; 6 Jun. 2004; M. Kapsali leg.; MAegean • 3 ♀♀; Agiassos; 39°03'00"N, 26°22'60"E; 23 May 2004; A. Kyriakopoulos leg.; MAegean • 1 ♂; same data as for preceding; 24 May 2001; MAegean • 1 ♀; same data as for preceding; MAegean • 1 ♂; Agiassos; 39°03'00"N, 26°22'60"E; Mar. 2006; C. Pérez-Bañón leg.; CEUA • 4 ♂♂; same data as for preceding; 11 Jun. 2005; CEUA • 4 ♂♂; same data as for preceding; 10 Jun. 2005; CEUA • 8 ♀♀; same data as for preceding; CEUA • 4 ♀♀; same data as for preceding; 6 Jun. 2005; CEUA • 4 ♂♂; same data as for preceding; CEUA • 1 ♂; Agiassos; 39°03'09"N, 26°22'57"E; 860 m a.s.l.; 23 May 2004; M. Kapsali leg.; MAegean • 2 ♂♂; 3.5 km S Agiassos; 39°03'09"N, 26°22'57"E; 860 m a.s.l.; 23 May 2004; M. Kapsali leg.; FSUNS 02487, 02503 • 1 ♂; 3.5 km S Agiassos; 39°03'09"N, 26°22'57"E; 860 m a.s.l.; 23 May 2004; A. Kyriakopoulos leg.; FSUNS 03762 • 1 ♀; 3.5 km S Agiassos; 39°03'09"N, 26°22'57"E; 860 m a.s.l.; 23 May 2004; M. Kapsali leg.; FSUNS 03764 • 3 ♀♀; Agiassos; 39°03'17"N, 26°23'50"E; 760 m a.s.l.; 10 Jun. 2004; M. Kapsali leg.; MAegean • 1 ♂; same data as for preceding; 24 May 2004; MAegean • 2 ♂♂; same data as for preceding; A. Kyriakopoulos leg.; MAegean • 1 ♀; same data as for preceding; MAegean • 2 ♀♀; same data as for preceding; 10 Jun. 2004; MAegean • 1 ♀; 3.8 km SSE Agiassos; 39°03'17"N, 26°23'50"E; 760 m a.s.l., 10 Jun. 2004; A. Kyriakopoulos leg.; FSUNS 03763 • 1 ♀; Agiassos; 39°03'45"N, 26°23'30"E; 700 m a.s.l.; 20 May 2004; A. Kyriakopoulos leg.; MAegean • 1 ♀; same data as for preceding; 6 Jun. 2004; M. Kapsali leg.; MAegean • 1 ♂; Agiassos; 39°03'92"N, 26°22'87"E; 27 May 2009; M. Taylor leg.; MZH http://id.luomus.fi/GJ.1133 • 1 ♂; Agiassos; 39°04'09"N, 26°23'17"E; 600 m a.s.l.; 15 May 2004; T. Petanidou leg.; MAegean • 5 ♂♂; Agiassos; 39°04'17"N, 26°22'22"E; Sep. 2009; A. Vujić leg.; FSUNS Č64, Č65, Ž9 to Ž11 • 10 ♀♀; Agiassos; 39°04'17"N, 26°22'22"E; Sep. 2009; A. Vujić leg.; FSUNS Ž12 to Ž19, Ž28, Ž29 • 2 ♂♂; Agiassos; 39°04'17"N, 26°22'22"E; 8 Jun. 2009; G. Ståhls leg.; MZH GJ.1139, GJ.1141• 4 ♀♀; same data as for preceding; MZH GJ.1135 to GJ.1138 • 4 ♂♂; same data as for preceding; 25 May 2009; MZH GJ.1140, GJ.1142, GJ.1143, GJ.1145 • 1 ♂; Agiassos; 39°04'25"N, 26°22'35"E; 8 May 2007; G. Ståhls leg.; MZH GJ.1144 • 1 ♀; same data as for preceding; MZH GJ.1126 • 5 ♂♂; same data as for preceding; 30 May 2009; MZH GJ.1119 to GJ.1123 • 2 ♀♀; same data as for preceding; MZH GJ.1124, GJ.1125 • 1 ♂; same data as for preceding; 27 May 2009; MZH • 1 ♂; 39°10'17"N, 26°18'14"E; FSUNS 02504 • 1 ♀; 39°10'17"N, 26°18'14"E; FSUNS 02505 • 3 ♀♀; 39°10'17"N, 26°18'14"E; 4 Jun. 2012; A. Vujić, L. Likov leg.; FSUNS G1747 to G1749 • 1 ♂; Vatousa; 39°13'51"N, 26°01'23"E; 200 m a.s.l.; 28 May 2001; FSUNS • 1 ♀; 2.5 km S Gavathas; 39°14'54"N, 25°58'60"E; 28 Apr. 2010; M. Hull leg.; WML 05042 • 6 ♂♂; near Sikaminea; 39°21'14"N, 26°17'56"E; 11 May 2009; G. Ståhls leg.; MZH GJ.1127 to GJ.1129, GJ.1132, GJ.1134, GJ.1147 • 2 ♀♀; same data as for preceding; 2 Jun. 2009; MZH GJ.1130, GJ.1131 • 2 ♀♀; 5.7 km NW Mantamados; 39°21'19"N, 26°17'52"E; 600 m a.s.l.; 2–10 May 2001; FSUNS 02488, 02507 • 3 ♂♂; Mantamados; 39°21'19"N, 26°17'52"E; 600 m a.s.l.; 10 May 2001; FSUNS • 2 ♀♀; same data as for preceding; FSUNS • 3 ♂♂; same data as for preceding; 17 May 2001; FSUNS • 1 ♀; same data as for preceding; FSUNS • 1 ♂; same data as for preceding; 23 May 2001; FSUNS • 1 ♂; Sikaminia; 39°21'42"N, 26°17'47"E; 10 May 2001; C. Pérez-Bañón, S. Rojo leg.; CEUA • 3 ♂♂; same data as for preceding; 14 May 2001; CEUA • 15 ♂♂; same data as for preceding; 17 May 2001; CEUA • 1 ♀; same data as for preceding; CEUA • 2 ♂♂; Sikaminia; 39°21'44"N, 26°17'49"E; 3 May 2008; A. Vujić leg.; FSUNS 03757, 03759 • 1 ♂; Sikaminia; 39°21'44"N, 26°17'49"E; 2 May 2001; FSUNS 02506 • 1 ♀; Near Lepetimnos; 39°21'47"N, 26°16'32"E; 1 May 2016; A. Vujić, J. Ačanski leg.; FSUNS 11243.

Turkey • 3 ♀♀; 12 km SW of Muğla; 37°07'40"N, 28°16'28"E; 660 m a.s.l.; 23 May 2011; M. Bartak, Kubik leg.; M. B. coll. 17921 to 17923 • 26 ♂♂; Muğla, University Campus; 37°09'42"N, 28°22'13"E; 700 m a.s.l.; 17–22 May 2011; M. Bartak, Kubik leg.; M. B. coll. 17927, 17928, 17930, 17931, 17937, 17938, 17940 to 17943, 17946, 17951, 17955 to 17960, 17965 to 17972 • 20 ♀♀; Muğla, University Campus; 37°09'42"N, 28°22'13"E; 700 m a.s.l.; 17–22 May 2011; M. Bartak, Kubik leg.; M. B. coll. 17929, 17932 to 17936, 17939, 17944, 17945, 17947 to 17950, 17952 to 17954, 17961 to 17964 • 1 ♀; Muğla, University campus; 37°09'42"N, 28°22'21"E; 700 m a.s.l.; Apr.–May 2014; O. Dursun leg.; M. B. coll. 10463 • 3 ♀♀; Muğla, 13 km NE pine wood; 37°14'50"N, 28°30'00"E; 1200 m a.s.l.; 23–27 Jun. 2015; M. Bartak, Kubik leg.; M. B. coll. 17924 to 17926 • 4 ♀♀; Bozdağ mountain, Near Bozdağ; 38°22'28"N, 28°04'38"E; 1140 m a.s.l.; 7 Jun. 2014; A. Vujić, J. Ačanski leg.; FSUNS 06945, 06946, 06948, 06951 • 2 ♂♂; Bozdağ mountain, Near Bozdağ; 38°22'28"N, 28°04'38"E; 1140 m a.s.l.; 7 Jun. 2014; A. Vujić, J. Ačanski leg.; FSUNS 06947, 06949.

##### Other material.

Greece, Lesvos • 1 ♀; Agiassos; 39°03'00"N, 26°22'60"E; 28 May 2010; Horsfield leg.; NMS • 1 ♀; Agiassos; 39°03'00"N, 26°22'60"E; 3 Jun. 2010; Wilkinson leg.; NMS [published in Ricarte et al. (2012), as *Merodon serrulatus*] • 1 ♀; same data as for preceding; 8 Jun. 2010; NMS • 1 ♀; Agiassos; 39°03'00"N, 26°22'60"E; 1–8 Jun. 2010; Hancock leg.; GLAHM • 2 ♂♂; same data as for preceding; 28–29 May–Jun. 2010; GLAHM • 2 ♀; Agiassos; 39°03'36"N, 26°23'30"E; 1 Jun. 2010; Horsfield leg.; NMS [published in Ricarte et al. (2012), as *Merodon serrulatus*] • 1 ♀; Agiassos; 39°03'36"N, 26°23'30"E; 28 May 2010; Horsfield leg.; NMS • 1 ♂; 3.5 km S Agiassos; 39°04'15"N 26°22'17"E; 860 m a.s.l.; 8 Jun. 2004; M. Kapsali leg.; MZH • 1 ♂; Agiassos; 39°04'17"N, 26°22'22"E; 1910 m a.s.l.; 26 May 2003; C. J. Palmer leg.; WML 298/03 • 1 ♀; same data as for preceding; WML 310/03 • 1 ♂; SW from Agiassos; 39°04'35"N, 26°22'03"E; 24 May 1988; NBCN • 1 ♀; Potamia river; 39°13'36"N, 26°06'48"E; 1–8 Jun. 2010; Hancock leg.; GLAHM • 1 ♂; 2 km S Gavathas; 39°16'11"N, 25°58'33"E; 5 May 2005; M. Hull leg.; WML • 1 ♂; 5.7 km NW Mantamados; 39°21'19"N, 26°17'52"E; 31 May 2004; E. Lamborn leg.; MZH.

#### Merodon sacki

Taxon classificationAnimaliaDipteraSyrphidae

(Paramonov, 1936)

777DD37A-62F9-53C1-B578-3C8FEBCB004E

Figs 8C , 9D–F , 10C, D , 11D–F

##### Diagnosis.

Large (9.5–11.6 mm) dark brown species with lack of microtrichose fasciae on terga 2–4 in males (Fig. 10C) and curved and very incrassate metafemur with long pile on ventral margin; the longest pile as long as half of width of metafemur (Fig. 8C). Similar to *Merodon bequaerti* but differs by strongly curved metafemur and generally longer body pile, clearly visible on tergum 4 (Fig. 10D).

##### Redescription

(based on holotype and additional material from the type area, Spain). **Male.** Head. Antennae black to dark brown; basoflagellomere ca. two times as long as wide, and ca. two times as long as pedicel, concave dorsally; large fossette dorsolateral; arista dark and thickened at basal one third, covered with dense microtrichia, 1.6 times as long as basoflagellomere (Fig. 11D–F); face and frons black with gray microtrichia, face covered with dense whitish gray, and frons with yellowish gray pile; oral margin microtrichose with shiny lateral areas; lunule shiny black, bare; vertex covered with golden microtrichia around ocellar triangle; vertex isosceles, with long, pale whitish yellow pile mixed with black pile on the ocellar triangle; ocellar triangle equilateral; eyes covered with dense pile; occiput with gray-yellow pile, covered with a dense, gray microtrichia; eye contiguity 10–14 facets long.

Thorax. Scutum and scutellum black with bronze luster, covered with dense, erect, yellow pile; scutum at wing basis with short black pile; scutum with two or more microtrichose vittae, anteriorly connected and posteriorly reaching the scutellum; scutum dull; posterodorsal part of anterior anepisternum, posterior anepisternum (except anteroventral angle), anterior anepimeron, dorsomedial anepimeron, and posterodorsal and anteroventral parts of katepisternum with long, pale yellow pile and grayish microtrichia; wings entirely covered with microtrichia; wing veins brown; calypteres and halteres pale yellowish; legs mostly black, except brown tarsi ventrally in some specimens; pile on legs pale yellow, except black pile at apical one fourth of metafemur; metafemur curved and incrassate, approximately three to four times longer than wide; pile on postero- and anteroventral surface long, and ca. half of width of metafemur (Fig. 8C).

Abdomen. Broad, tapering, 1.2 times longer than mesonotum; terga dark brown to black, usually without microtrichose fasciae; tergum 2 with orange lateral maculae; pile on terga all yellow (Fig. 10C, D); sterna dark brown, covered with long whitish yellow pile.

Male genitalia. Apical part of anterior surstyle lobe rhomboid in shape, 1.5 times longer than wide, covered with dense, short pile (Fig. 9D: al); posterior surstyle lobe oval with basolateral protrusion (lateral hump) (Fig. 9D, E: bp); hypandrium sickle-shaped, without lateral projections; lingula large (Fig. 9F: l).

**Female.** Unknown.

##### Distribution.

*Merodon sacki* is known only from Spain (Fig. 7).

##### Ecology.

Preferred environment: forest/open ground; open areas in evergreen oak forest (*Quercus ilex* and *Q. suber*) and Mediterranean scrub. Flowers visited: no data. Flight period: April-July.

##### Type material.

Holotype (original designation): male, “Holotypus Lampetia / sacki Paramononv, 1936 / G.V. POPOV des. 2007” [red label], “*Lampetia* / *sacki n. sp.* / ♂ *Typus* / *Paramonov d.*” [pink label handwritten], “*Merodon* / *mir únbekannt*” [yellow label handwritten], “*14 VII 81*” “*Chiclana*” [handwritten on the back side] (SIZK) (See Supplementary file 6: Figure 6B) (studied).

Note (Popov pers. comm.). The species was described by examining a single male, with the type clearly indicated on the label by Paramonov (discovered and deposited in SIZK). The type specimen is considered lost (Liepa 1969). Hurkmans (1993: 178, 179) incorrectly considered *M. sacki* as a junior synonym of *M. clavipes* (Fabricius, 1781). Hurkmans (1993) also provided an incorrect year for the description of *Lampetia sacki* (1937 instead of the correct 1936), and also incorrectly designated the lectotype and paralectotype [Articles 73 and 74 of the ICZN (1999)] for two *M. clavipes* females with the same label “Chiklana”, which are not syntypes [a violation of Articles 74.1 and 74.2 of ICZN (1999)]. The holotype was established by the original designation according to Article 73.1.1 of the ICZN (1999), as well as by a monotype according to Article 73.1.2 (ibid.).

##### Other material.

Spain • 3 ♂♂; La Corte; 37°57'41"N, 6°49'09"W; 28 Apr. 2015; A. Vujić, D. Obreht leg.; FSUNS 09340, 09343, 09345.

#### Merodon serrulatus

Taxon classificationAnimaliaDipteraSyrphidae

(Wiedemann in Meigen, 1822)

13F007B4-C081-58EC-892E-77228DCD6BAF

Figs 1A–G , 2A–D , 3A–J , 4A–F , 5A–D , 6A–G , 14C

Merodon alexeji Paramonov, 1925: 155 – syn. published in Vujić et al. 2011: 84.
Merodon lusitanicus Hurkmans, 1993: 181 – syn. published in Marcos-García et al. 2007: 566.
Merodon tener Sack, 1913: 443 syn. nov.

##### Diagnosis.

Medium sized (7.1–10.9 mm), short pilose dark species with olive-brown reflection; antennae dark brown; legs mostly black; body pile predominantly pale yellow, except black pile on vertex and scutum, terga 2–4 in some specimens and apical one third of femora in some specimens and populations; basoflagellomere elongated (1.7–2.2 times as long as wide) obviously concave dorsally, arista short (Fig. 3); tergum 2 usually with small pale orange-yellow lateral maculae (Fig. 2); metafemur incrassate, ca. three times longer than wide, with short pilosity, except few long pile on postero- and anteroventral surface of metafemur (Fig. 4); male genitalia: apical part of anterior surstyle lobe triangular (Fig. 1A: al, C, D); posterior surstyle lobe with lateral hump (Fig. 1A, B: lp); lingula large (Fig. 1E: l).

##### Redescription

(based on the types and specimens from the type area of nominal taxon, Iberian Peninsula; variability includes populations from all of the range). **Male.** Head. Antennae black to dark brown; basoflagellomere (Fig. 3A–C, G–J) elongated, 1.7–2.2 times as long as wide, and 2.5–3 times as long as pedicel, concave dorsally, tapering to the apex; dorsolateral and dorsomedial (if present) fossette large with variable shape (see variability) (as on Fig. 3); arista dark and thickened at basal one third, covered with dense microtrichia; arista short, 1.2–1.5 times as long as basoflagellomere (Fig. 3); face and frons black with gray microtrichia, face covered with dense whitish, and frons with yellowish gray pile; oral margin shiny, with small lateral microtrichose area; lunule shiny black, bare; eye contiguity 8–10 facets long; vertex isosceles, shiny black, except in front of anterior ocellus, covered with microtrichia; vertex with long, pale whitish yellow pile, in some cases mixed with few black pile on the ocellar triangle; ocellar triangle from equilateral to isosceles (see variability); occiput with gray-yellow pile, ventrally covered with a dense, gray microtrichia; eyes covered with dense whitish pile (Fig. 5A, B); ratio of length of vertical triangle: eye contiguity: frons = 3 : 1 : 3.

Thorax. Scutum and scutellum black with bronze luster, covered with dense, erect, usually yellow pile; scutum at wing basis in some specimens and populations with patch of black pile, or with fascia of black pile between wing basis; scutum usually with two or four microtrichose vittae (see variability), anteriorly connected and posteriorly reaching the scutellum (Fig. 2A); anterior half of scutum from dull until shiny black (see variability); posterodorsal part of anterior anepisternum, posterior anepisternum (except anteroventral angle), anterior anepimeron, dorsomedial anepimeron, and posterodorsal and anteroventral parts of katepisternum with long, dense pale yellow pile and grayish microtrichia; wings entirely covered with microtrichia; wing veins brown; calypteres pale yellow; halteres yellow, in some cases with dark capitulum; legs (Fig. 4) without spinae or other protuberances; legs mostly black, except brown tarsi ventrally in some specimens; pile on legs pale yellow, except black pile at apical one third of metafemur in some populations (see variability); metafemur moderately incrassate, ca. three times longer than wide; long pile on postero- and anteroventral surface sparse, and ca. one third to one fourth (see variability) of width of metafemur, approximately the same length as pile on dorsal surface (Fig. 4).

Abdomen. Tapering posteriorly, ca. 1.2 times longer than mesonotum; terga dark brown to black, except for a pair of pale yellow-orange, triangular, lateral maculae on tergum 2 (in some specimens less visible: see variability); terga 3 and 4 each with a pairs of white microtrichose, oblique fasciae (on tergum 2 triangular); color of pile on terga variable, from all yellow to specimens with many black pile on terga 2–4 (see variability) (Fig. 2C, D); sterna dark brown, covered with long whitish yellow pile.

Male genitalia. Apical part of anterior surstyle lobe triangular shape, 1.1–1.4 times longer than wide, covered with dense, short pile (Fig. 1A: al, C, D); posterior surstyle lobe oval with basolateral protrusion (lateral hump) (Fig. 1A, B: bp, 14C: bp); cercus rectangular (Fig. 1A: c); hypandrium sickle-shaped, without lateral projections; lingula large (Fig. 1E: l).

**Female.** Similar to the male except for normal sexual dimorphism and for the following characteristics: antennae with rounded tip, basoflagellomere ca. two times longer than wide, fossette dorsal (Fig. 3D–F); frons with microtrichose vittae along eye margins variable in shape and size (see variability); frons covered with variable color of pilosity, from mostly gray-yellow until predominately black (see variability) (Fig. 5C, D); ocellar triangle covered with black pile; lateral side of terga, anterior two third of tergum 2 and all tergum 5 with yellow pile; central part of terga 2–4 with short adpressed black pile; microtrichose fasciae on terga 3 and 4 conspicuous (Fig. 2D).

##### Variability.

There is some intra- and interpopulation variability in the morphological characters of *Merodon serrulatus*, which are summarized in Table 1.

**Table 1.** Inter- and intrapopulation variability of *Merodon serrulatus*.

**Character**	**Variability (intra- and/or interpopulation)**	**Intra**	**Inter**
color of antenna	from black to brown	+	-
length of basoflagellomere	1.7–2.2 times as long as wide	+	+ shorter in Balkans populations (1.7–1.9)
position of antennal fossette in male	dorsal to lateral or dorsal and medial (Fig. 3)	+	+ Iberian populations with medial fossette
length of arista	1.0–1.5 times as long as basoflagellomere	-	+ longer in Balkans populations (1.4)
ocellar triangle in male	equilateral or isosceles	+	-
microtrichose vittae on scutum	from 2–4, posterior half dull without microtrichia	+	-
black pile on scutum	few, or fascia of black pile between wing basis, or many black pile on scutum	+	-
color of pile on metafemur in male	all yellowish to whitish or with many black in apical one third	+	+ some Iberian populations with only pale pile
length of pile on metafemur in male	one third to one fifth of width of metafemur	+	+ in eastern populations (southern Russia to Siberia) longer, from one third to one fourth of width of metafemur
color of knees, apex of tibiae and tarsi	from black to brown	+	-
lateral maculae on tergum 2	distinct, indistinct, to almost absent	+	-
pile on terga 3–4 in male	from all pale yellow to many black	+	+ Balkans populations with more black pile
microtrichose vittae on frons in female	from narrow unconnected to broad and connected near ocellar triangle	+	-
color of pile on frons in female	almost all black to mostly whitish	+	-
male genitalia	the shape of surstyle and size of area covered with dense short marginal setulae on anterior surstyle lobe (Figs 1, 6)	+	+ in eastern populations (southern Russia to Siberia) basolateral protrusion less distinct (Fig. 6G)

##### Distribution.

As shown in Fig. 7, this *Merodon* taxon is characterized by the greatest range, extending from Iberian Peninsula in the south-west, through Greece and eastern Turkey to the south, and eastward to Siberia and Mongolia (Doczkal pers. comm.).

##### Ecology.

Preferred environment: forest/open ground; thermophilous *Quercus* forest; *Castanea* forest (Ståhls et al. 2009), evergreen oak forest (*Quercus ilex* L. and *Q. suber* L.), dry *Pinus* forest; lentisc scrub; dry, well-vegetated, calcareous and non-calcareous unimproved grassland and tracksides; hedgehog heath (Speight 2018); *Pinus*, *Picea*, and *Larix* forests (Siberia) (Fig. 35B). Flowers visited: Umbelliferae; *Cirsium* spp., *Helianthemum* spp., *Potentilla* spp., *Rosa* spp., *Thapsia* spp., and *Thymus* spp. (Speight 2018). Flight period: April-August.

##### Type material.

Holotype of *Merodon serrulatus* [original designation in Meigen (1822: 360)]: Wiedemann in Meigen (1822) as *Lampetia serrulata*: “Portugal / Hoffmannsegg S.” 1 ♀, (ZHMB) (studied).

*Merodon alexeji*: Described by Paramonov (1925) based on two specimens (male and female). Lectotype [designated by Marcos-García et al. (2007)]: male, “*Merodon* / *alexeji n. sp.* / Typus / Paramonov d.”, “Kohanovka / Baltsk. u. / Odes. g. [in Cyrillic] 1.VI.24. Ucraina” (PC) (SIZK) (studied).

*Merodon lusitanicus*: Holotype [original designation by Hurkmans (1993: 181)]: female, “Portugal, Algarve, Quarteira 27.iv.1985, J.A.W. Lucas” (NBCN) (studied). Paratypes. Portugal • 1 ♀; Algarve, Quarteira; 37°03'29"N, 8°04'47"W; 27 Apr. 1985; NBCN • 1 ♀; Algarve, Vilamoura; 37°04'35"N, 8°07'46"W; 27 Apr. 1985; NBCN.

*Merodon tener*: Described by Sack (1913: 443) based on three male and three female syntypes. Lectotype [designated by Hurkmans (1993)]: female “Sarepta [= Krasnoarmeysk near Volgograd, after Peck 1988] / *M. tener* Sack det. Sack / coll. Lichtwardt / coll. D. E. I. Eberswalde” (ZHMB) (studied). Original label: “LECTOTYPE of / M. tener Sack / des. 1988 Hurkmans” [red label handwritten], “Sarepta” [yellow label handwritten], “M. tener Sack / ♀ det. Sack” [label partly handwritten], “Coll. DEI / Eberswalde”, “Coll. Lichtwardt”. (See Supplementary file 6: Figure 6A). Lectotype is conspecific with type of *M. serrulatus*, sharing the same morphological characters.

##### Other material.

Croatia • 1 ♀; Velebit, Brušane; 44°29'55"N, 15°16'43"E; 600 m a.s.l.; 13 Jun. 1969; NBCN 02489.

France • 1 ♀; Languedoc-Roussillon, Corbieres, Carcassonne; 43°13'00"N, 2°21'00"E; 18 Jun. 1974; NBCN • 1 ♂; Provence Alpes Cote d’Azur, Saint-Maximin-la-Sainte-Baume; 43°25'23"N, 5°50'11"E; 17–20 Jun. 1951; M. Bequaert leg.; NBCN 02491 • 1 ♀; same data as for preceding; NBCN 02492 • 1 ♀; Source du Lez, Saint Clement; 43°43'05"N, 3°50'39"E; 24 May 1989; Maldes leg.; MNHN 22629 • 1 ♂; Provence Alpes Cote d’Azur, Montagne du Luberon, W from Bonnieux; 43°48'00"N, 5°22'00"E; 3 Jun. 1993; NBCN • 1 ♀; Feuilla, Route de Treilles, en face du village Panais; 43°53'53"N, 2°00'16"E; 7 Jun. 1988; J. Hamon leg.; MNHN 17973 • 3 ♂♂; Departement du Gard, Mas Mejean; 44°05'24"N, 3°35'26"E; 29 May 1952; NBCN • 2 ♂♂; P. N. Mercantour, Le Bor, on, Umgebung, mesophiles pot. Argent; 44°06'47"N, 7°16'42"E; 1380 m a.s.l.; 21 Jun. 2011; A. Ssymank leg.; A. S. coll. G1057 • 1 ♂; Causse de Sauveterre; 44°22'03"N, 3°13'49"E; 20 Jul. 1971; MNHN 22628 • 1 ♀; Larche (Basses Alpes); 44°26'59"N, 6°50'60"E; 22 Jul. 1925; R. Benoist leg.; MNHN PM0383 • 1 ♂; same data as for preceding; MNHN PM0429 • 1 ♂; same data as for preceding; 3 Jul. 1925; MNHN 22630 • 1 ♂; Provence Alpes Cote d’Azur, Larche; 44°26'59"N, 6°50'60"E; 22 Jul. 1923; NBCN • 1 ♂; Drome, La Chapelle en Vercors; 44°58'12"N, 5°23'39"E; 28 Jun. 1970; Roman Emile leg.; MNHN PM0377 • 2 ♂♂; Isere, Villars de Lans Pic st Michel hill top; 45°05'24"N, 5°37'12"E; 1970 m a.s.l.; 20 Jul. 2010; J. van Steenis leg.; J. v. S. coll. • 1 ♀; L’Arselle; 45°23'10"N, 7°04'19"E; 14 Jun. 1909; MNHN PM0357.

Greece • 2 ♀♀; Mountain Taygetos; 22 km SW Sparta; 36°58'60"N, 22°24'09"E; 6 May 1990; NBCN • 1 ♂; Mountain Taygetos; 37°05'20"N, 22°18'55"E; 950–1800 m a.s.l.; 15–19 May 1990; ZMUC 00513256 • 7 ♂♂; Laconia, Karyes, 25 km N from Sparta; 37°18'15"N, 22°25'16"E; 930 m a.s.l.; 23 May 2014; A. Vujić, J. Ačanski leg.; FSUNS 06535, 06542, 06547, 06549, 06556, 06563, 06560 • 6 ♀♀; Laconia, Karyes, 25 km N from Sparta; 37°18'15"N, 22°25'16"E; 930 m a.s.l.; 23 May 2014; A. Vujić, J. Ačanski leg.; FSUNS 06543, 06544, 06546, 06553, 06555, 06565 • 3 ♂♂; Chelmos, Kalavryta ski center; 38°00'25"N, 22°11'40"E; 6 Jun. 2017; A. Vujić, Z. Nedeljković, L. Likov, M. Miličić, T. Tot, leg.; FSUNS 15980 to 15982 • 2 ♀♀; Chelmos, Kalavryta ski center; 38°00'25"N, 22°11'40"E; 6 Jun. 2017; A. Vujić, Z. Nedeljković, L. Likov, M. Miličić, T. Tot leg.; FSUNS 15983, 15984 • 1 ♂; Achaia, Mountain Chelmos above Kalavryta; 38°00'31"N, 22°07'08"E; 1700 m a.s.l.; 17–19 Jun. 1982; B. Skule, S. Langemark leg.; ZMUC 00513264 • 3 ♀♀; Achaia, Mountain Chelmos above Kalavryta; 38°00'31"N, 22°07'08"E; 1700 m a.s.l.; 17–19 Jun. 1982; B. Skule, S. Langemark leg.; ZMUC 00513265, 00513273, 00513301 • 2 ♂♂; Corfu; 39°40'00"N, 19°45'00"E; NHMW 02485, 02486 • 2 ♂♂; Corfu; 39°40'00"N, 19°45'00"E; 1400 m a.s.l.; NHMW • 1 ♀; Peristeri mountain; 39°40'36"N, 21°07'06"E; 2030 m a.s.l.; 24–28 May 1994; V. Michelsen leg.; ZMUC 00513259; • 1 ♂; 15 km NO Metsovo; 39°47'19"N, 21°11'58"E; 4 Jun. 1994; M. Ohl leg.; ZHMB • 4 ♂♂; Mountain Pindos, Katara Pass; 39°47'46"N, 21°13'44"E; 20 May 1997; S. Radenković leg.; FSUNS • 3 ♂♂; same data as for preceding; S. Šimić leg.; FSUNS • 4 ♀♀; same data as for preceding; A. Vujić leg.; FSUNS • 6 ♂♂; Mountain Pindos, Katara Pass; 39°47'48"N, 21°13'45"E; 1700 m a.s.l.; 20 May 1997; FSUNS 01779, 01781 to 01785 • 3 ♀♀; same data as for preceding; FSUNS 01786, 01787, 01780 • 1 ♀; Mountain Pindos, Katara Pass; 39°54'00"N, 21°11'00"E; 13 Jul. 1979; M. C. D Day, G. R. Else, D. Morgan leg.; NHMUK • 1 ♂; Mountain Pindos, “Iznad Panagije” [Panagia]; 39°48'25"N, 21°19'44"E; 850 m a.s.l.; 15 May 2011; A. Vujić leg.; FSUNS H38 • 15 ♂♂; Mountain Olympos, Litochoras-Prionia 3, “proplanak pored puta”; 40°04'36"N, 22°00'46"E; 17 May 2012; A. Vujić leg.; FSUNS H82, H83, H86 to H90, H92, H93, H95 to H97, H99, I6, I7 • 1 ♂; Mountain Olympos, Litochoras-Prionia 3, “proplanak pored puta”; 40°04'36"N, 22°00'46"E; 17 May 2012; FSUNS H94 • 1 ♂; Mountain Olympos, Near Litochoro; 40°06'30"N, 22°28'41"E; 21 May 2014; A. Vujić, J. Ačanski leg.; FSUNS 06499 • 1 ♀; Mt Olympos, Litochoro; 40°06'41"N, 22°28'37"E; 650 m a.s.l.; 17 May 2016; A. Vujić, J. Ačanski, M. Miličić, Z. Nedeljković leg.; FSUNS 11679 • 8 ♂♂; Mountain Olympos, Litochoras-Prionia 4; 40°06'43"N, 22°28'08"E; 18 May 2011; A. Vujić leg.; FSUNS I11 to I14, I17, I20 to I22 • 2 ♀♀; same data as for preceding; FSUNS I15, I16.

Italy • 1 ♀; Sicily, Etna, Rifugio Filiciusa; 37°43'14"N, 15°02'51"E; 1400–1500 m a.s.l.; 22–28 Jul. 1961; V. S. van der Goot leg.; NBCN • 1 ♂; Toscana, Florence, Careggi; 43°48'45"N, 11°15'07"E; 19 May 1986; NBCN • 2 ♂♂; Piedmont, Colle di Sestrieres; 44°57'00"N, 6°52'60"E; 1800/2100 m a.s.l.; 23–31 Jul. 1837; Zerny leg.; NHMW.

MONTENEGRO • 1 ♂; Lovćen, Lovćen 1; 42°22'59"N, 18°53'54"E; 17 May 2018; A. Vujić, A. Šebić, M. Ranković leg.; FSUNS 19017 • 1 ♂; Boka Kotorska, Morinj; 42°29'25"N, 18°38'56"E; 16–18 May 1998; FSUNS 03600 • 1 ♂; same data as for preceding; 18–19 May 1998; FSUNS 03601 • 2 ♀♀; same data as for preceding; FSUNS 03602, 03603.

Kazakhstan • 1 ♀; East Kazakhstan, Markakol’ District, 20 km N settlement Alekseevka, Souther slop of Matobaj Mountain range; 48°42'22"N, 85°57'00"E; 2318 m a.s.l.; 6 Jul. 1996; V. Zinchenko leg.; SZMN • 1 ♀; Kazakhstan, 9 km S settlement Karaoj, Kyzyl-Tass Mountain; 29 Jun. 1996; V. Zinchenko leg.; SZMN.

NORTH MACEDONIA • 1 ♂; Kožuf, Golema poljana; 41°10'54"N, 22°12'05"E; 15 Jun. 1955; FSUNS 00165 • 1 ♂; same data as for preceding; 18 Jun. 1956; FSUNS 00166 • 5 ♂♂; Kožuf, Golema poljana; 41°10'54"N, 22°12'05"E; 14 Jun. 1975; FSUNS 00154, 00156 to 00158, 00162 • 1 ♀; Kožuf, Golema poljana; 41°10'54"N, 22°12'05"E; 15 Jun. 1968; FSUNS 00167 • 2 ♀♀; same data as for preceding; 17 Jun. 1956; FSUNS 00168, 00169 • 6 ♀♀; Kožuf, Golema poljana; 41°10'54"N, 22°12'05"E; 14 Jun. 1975; FSUNS 00155, 00159 to 00161, 00163, 00164 • 2 ♀♀; Kožuf; 41°25'38"N, 21°30'45"E; 14 Jun. 1975; FSUNS 00152, 00153.

Russia • 1 ♂; Sarepta, “Russia, RUS, Sarepta, now a suburb of Volgograd city (Christoph)”; 48°31'40"N, 44°29'01"E; ZHMB 02501 • 4 ♀♀; same data as for preceding; ZHMB 02500, 02502, 02508, 02509 • 32 ♂♂; Altai, 10 km S-W of Katanda; 50°06'51"N, 86°07'21"E; 6 Jul. 1983; A. Barkalov leg.; SZMN • 9 ♀♀; same data as for preceding; SZMN • 1 ♂; SW Altai, Katun valley 10 km W Katanda; 50°09'46"N, 86°06'50"E; 7 Jul. 1983; H. Hippa leg.; MZH • 1 ♂; same data as for preceding; 22–27 Jun. 1983; MZH; “exp. Mikkola, Hippa et Jalava” • 1 ♂; Altai, Kurayskaya Step’; 50°12'00"N, 87°47'60"E; 1662 m a.s.l.; 9 Jul. 2006; A. Barkalov leg.; SZMN • 17 specimens; Altai, Terekta; 50°16'12"N, 85°58'12"E; 1098 m a.s.l.; Jun. 1973; SZMN • 1 ♂; Tuva, Erzin river; 50°19'12"N, 95°30'00"E; 1288 m a.s.l.; 27 Jun.–1. Jul. 1989; D. Logunov leg.; SZMN • 16 ♀♀; same data as for preceding; SZMN • 2 ♂♂; Tuva, Tere Khol’ Lake; 50°42'11"N, 97°20'2"E; 27 Jun.–1 Jul. 1989; D. Logunov leg.; SZMN • 1 specimen; Altai, Tuyekta; 50°51'00"N, 85°49'48"E; 944 m a.s.l.; Jun. 1979; SZMN • 2 specimens; Tuva, Chagytay; 50°58'30"N, 94°38'47"E; 1963; SZMN • 1 specimen; Altai, Baragash; 51°16'48"N, 85°12'36"E; 927 m a.s.l.; Jun. 1973; SZMN • 2 ♀♀; Altaj, Erlagol; 51°22'21"N, 86°05'22"E; 27 Jun. 1995; FSUNS 00171, 00173 • 3 ♂♂; Altaj, Erlagol; 51°22'23"N, 86°05'29"E; 27 Jun. 1995; A. Tepavčević leg.; FSUNS 00170, 00172, 02476 • 1 ♀; same data as for preceding; FSUNS 02477 • 1 ♂; Gornyi Altai, Turochaksky r-n kordon Obogo; 51°35'47"N, 87°05'45"E; 950 m a.s.l.; 15 Jun. 2003; D. Kropačeva leg.; SZMN 22631 • 18 ♂♂; Altai mountains, Teletskoye Lake; 51°41'20"N, 87°33'43"E; 23–25 Jun. 2013; A. Vujić, S. Radenković leg.; FSUNS NJ56, NJ57, NJ59 to NJ62, NJ64 to NJ71, NJ73 to NJ75, NJ77 • 5 ♀♀; same data as for preceding; FSUNS NJ63, NJ72, NJ76, NJ78, NJ79 • 2 ♂♂; Altai, Turochaksky r-n, Teletskoe Lake, 14 km S of Iogach; 51°42'0"N, 87°17'60"E; 598 m a.s.l.; 27 Jun. 2006; V. Zinchenko leg.; SZMN • 2 ♀♀; same data as for preceding; SZMN • 1 ♀; Mountain Ural, Orenburg; 51°46'12"N, 54°59'53"E; ZHMB 02519 • 15 ♂♂; Siberia, Altaya, Teletskoe Lake; 51°46'60"N, 87°18'00"E; 24–19 Jun. 2006; A. Barkalov, V. Zinchenko leg.; SZMN • 6 ♀♀; same data as for preceding; SZMN • 2 ♂♂; same data as for preceding; 27 Jun. 2006; J. T. Smit leg.; FSUNS 03972, 03973 • 1 ♀; same data as for preceding; 24 Jun. 2006; FSUNS 03974 • 1 ♂; Siberia, Republic Alatai, Teletskoe lake, Artybash.; 51°47'57"N, 87°14'58"E; 25 Jun. 1990; G. Ståhls leg.; MZH • 8 ♂♂; Altai, Teletskoe Lake, Artibash; 51°47'57"N, 87°14'58"E; 12 Jun. 1990; A. Barkalov, Čekanov leg.; SZMN • 1 ♀; same data as for preceding; SZMN • 17 ♂♂; same data as for preceding; 11–25 Jun. 1990; SZMN • 8 ♀♀; same data as for preceding; SZMN • 13 ♂♂; same data as for preceding; 18–20 Jun. 1990; SZMN • 10 ♀♀; same data as for preceding; SZMN • 1 ♀; same data as for preceding; 23 Jul. 1979; SZMN • 82 specimens; same data as for preceding; Jun. 1979; SZMN • 1 ♂; Altaj, Gorno-Altaysk; 51°57'08"N, 85°57'19"E; 21 Jun. 1983; MZH; “exp. Mikkola, Hippa et Jalava” • 152 specimens; Altai, Gorno-Altaysk; 51°57'08"N, 85°57'19"E; Jun.–Jul. 1979; A. Barkalov leg.; SZMN • 2 ♂♂; same data as for preceding; 22 Jun. 1983; SZMN • 3 ♀♀; same data as for preceding; SZMN • 1 ♂; Altai Republic; 52°30'00"N, 83°00'00"E; 25 Jun. 1979; NBCN • 1 ♀; same data as for preceding; NBCN • 2 ♂♂; Sayan Mountains, Abaza; 52°41'17"N, 90°05'27"E; 30 May–11 Jun. 1981; A. Barkalov, T. Varlamova leg.; SZMN • 7 specimens; same data as for preceding; Jun. 1969; SZMN • 2 specimens; Novosibirsk; 55°06'56"N, 82°51'33"E; 1972–1974; SZMN • 1 specimen; Tuva, Sosnovka; 56°18'18"N, 51°14'44"E; 1949; SZMN.

Spain • 1 ♂; Sierra Nevada, second valley; 37°06'10"N, 3°27'19"W; 1430 m a.s.l.; 17 Jun. 2014; A. Vujić, S. Radenković, C. Pérez-Bañón leg.; FSUNS 07410 • 3 ♂♂; Sierra Nevada, Ski Centar Sierra Nevada; 37°06'45"N, 3°25'10"W; 2190 m a.s.l.; 16 Jun. 2014; A. Vujić, S. Radenković, C. Pérez-Bañón leg.; FSUNS 07275, 07287, 07302 • 1 ♂; prov. Granada Sierra Nevada ri. Valetta; 37°06'55"N, 3°29'32"W; 1 Jun. 1982; NBCN • 11 ♂♂; Sierra Nevada, First valley; 37°07'40"N, 3°26'44"W; 1630 m a.s.l.; 17 Jun. 2014; A. Vujić, S. Radenković, C. Pérez-Bañón leg.; FSUNS 07325, 07326, 07328, 07338, 07342, 07344, 07358, 07368, 07371, 07373, 07384 • 10 ♀♀; same data as for preceding; FSUNS 07324, 07340, 07341, 07343, 07355, 07375, 07381, 07385, 07392, 07401 • 4 ♂♂; Sierra Nevada, road to hotel Duque; 37°08'17"N 3°25'46"W; 16 Jun. 2014; A. Vujić, S. Radenković, C. Pérez-Bañón leg.; FSUNS 07248, 07251, 07255, 07256 • 3 ♀♀; same data as for preceding; FSUNS 07262, 07263, 07265 • 1 ♂; Sierra Nevada Lugros, Horcajo del Camarate; 37°11'50"N, 3°15'13"W; 1370 m a.s.l.; 18 Jun. 2014; A. Vujić, S. Radenković, C. Pérez-Bañón leg.; FSUNS 07428 • 1 ♂; Andalusia, Sierra de Baza, Prados del Roy; 37°22'33"N, 2°51'06"W; 2000/2100 m a.s.l.; 9 Jun. 2003; D. Doczkal leg.; D. D. coll. 04805 • 1 ♀; same data as for preceding; D. D. coll. 04808 • 2 ♂♂; Andalusia, Sierra de Baza, Santa Barbara; 37°23'16"N, 2°50'43"W; 1890 m a.s.l.; 9 Jun. 2003; D. Doczkal leg.; D. D. coll. 04806, 04807 • 1 ♀; La Corte; 37°57'41"N, 6°49'09"W; 28 Apr. 2015; A. Vujić, D. Obreht leg.; FSUNS 09333 • 1 ♂; Alicante, Alcoy-Font Roja; 38°42'00"N, 00°28'00"W; 31 May 1994; P. M. Isidro leg.; FSUNS 02494 • 1 ♀; Valensija, Utiel; 39°34'13"N, 1°11'15"W; 9 May 1994; C. Pérez-Bañón leg.; FSUNS 02495 • 1 ♂; Val de Cabras; 40°09'23"N, 2°01'48"W; 10 Jun. 1980; H. G. M. Tenuissen leg.; NBCN • 1 ♂; between Leon and Oviedo, Puerto de Pajares; 43°00'00"N, 5°46'00"W; 12 Jul. 1972; NBCN.

Turkey • 5 ♂♂; “Kop Dağı geçidi” [Kop mountain pass], Bayburt; 40°15'00"N, 40°15'00"E; 16 Jul. 1992; NBCN.

#### Merodon sophron

Taxon classificationAnimaliaDipteraSyrphidae

Hurkmans, 1993

A85A9F8C-3E4F-5B4A-B97C-8D5800F9F5EB

Figs 8D , 9G–I, K , 11G–I

##### Diagnosis.

Medium sized (7.8–9.2 mm), dark species with olive-brown reflection; antennae dark; legs mostly black; body pile predominantly pale, except few black pile on vertex and scutum; basoflagellomere elongated (1.8 times as long as wide) obviously concave dorsally, arista 1.8 times as long as basoflagellomere (Fig. 11G–I); tergum 2 with pale lateral maculae; metafemur incrassate with medium long pile on ventral surface, length approximately one third of its width (Fig. 8D); male genitalia: posterior surstyle lobe with lateral hump; apical part of anterior surstyle lobe rhomboid; lingula medium size (Fig. 9G–I). Related to *Merodon serrulatus* from which differs in absence of medial fossette (Fig. 11H), present in geographically related Iberian populations of *M. serrulatus* (Fig. 3B), molecular data and distribution (Fig. 7). Related to *M. bequaerti*, but differs by shorter pile on ventral margin of metafemur in both sexes (Fig. 8D), narrower and oval to triangular apical part of anterior surstyle lobe (Fig. 9G), with rounded margin in *M. bequaerti* (Fig. 9A), and light yellow and less dense marginal pile on apical part of anterior surstyle lobe (Fig. 9G, K: al), dark brown and dense in *M. bequaerti* (Fig. 9A: al, J).

##### Redescription

(based on the material from type locality, Middle Atlas, Azrou). **Male.** Head. Antennae black to dark brown; basoflagellomere elongated, ca. 1.8 times as long as wide, and ca. 2.5 times as long as pedicel, concave dorsally with acute apex; fossette dorsolateral; arista dark and thickened at basal one third, covered with dense microtrichia, ca. 1.8 times as long as basoflagellomere (Fig. 11G, H); face and frons black with gray microtrichia, face covered with dense whitish, and frons with yellowish gray pile; oral margin shiny, with small lateral microtrichose area; lunule shiny black, bare; vertex shiny black, except in front of anterior ocellus, covered with microtrichia; vertex isosceles, with long, pale whitish yellow pile, mixed with black pile on the ocellar triangle; ocellar triangle isosceles; eyes covered with dense pile; occiput with gray-yellow pile, ventrally covered with a dense, gray microtrichia; eye contiguity 8–11 facets long; vertical triangle: eye contiguity: frons = 3 : 1 : 3.

Thorax. Scutum and scutellum black with bronze luster, covered with dense, erect, usually yellow pile; sides of scutum at wing basis with patch of black pile or fascia of short black pile and few black pile between wing basis; scutum with two microtrichose vittae, anteriorly connected and posteriorly reaching the scutellum; anterior half of scutum dull; posterodorsal part of anterior anepisternum, posterior anepisternum (except anteroventral angle), anterior anepimeron, dorsomedial anepimeron, and posterodorsal and anteroventral parts of katepisternum with long, dense pale yellow pile and grayish microtrichia; wings entirely covered with microtrichia; wing veins brown; calypteres and halteres pale yellow; legs mostly black, except brown tarsi ventrally in some specimens; pile on legs pale yellow; metafemur moderately incrassate, ca. three times longer than wide; pile on postero- and anteroventral surface medium long, and ca. as one third of width of metafemur, approximately the same length as pile on dorsal surface (Fig. 8D).

Abdomen. Tapering, 1.2 times longer than mesonotum; terga dark, except for a pair of pale yellow-orange, triangular, lateral maculae on tergum 2; terga 3 and 4 each with a pair of white microtrichose and oblique fasciae (on tergum 2 triangular); pile on terga all yellow; sterna dark brown, covered with long whitish yellow pile.

Male genitalia. Apical part of anterior surstyle lobe rhomboid shape, ca. 1.5 times longer than wide, covered with dense, short pile (Fig. 9G, J: al); posterior surstyle lobe oval with basolateral protrusion (lateral hump) (Fig. 9G, H: bp); hypandrium sickle-shaped, without lateral projections; lingula medium size (Fig. 9I: l).

**Female.** Similar to the male except for normal sexual dimorphism and for the following characteristics: antennae with rounded tip, basoflagellomere ca. two times longer than wide (Fig. 11I); frons with broad microtrichose vittae along eye margins; frons covered with variable pilosity, from mostly gray-yellow until predominantly black; ocellar triangle covered with black pile; lateral side of terga, anterior two thirds of tergum 2 and all of tergum 5 with yellow pile; terga 2–4 with short adpressed black pile.

##### Distribution.

*Merodon sophron* is distributed in north-western Africa (Morocco) (Fig. 7).

##### Ecology.

Preferred environment: forest/open ground; open areas in evergreen oak maquis, dry *Pinus* forest; unimproved grassland and tracksides (Fig. 35C). Flowers visited: no data. Flight period: May-June.

##### Type material.

Holotype [original designation by Hurkmans (1993: 168)]. Morocco • ♂; Azrou; 33°25'00"N, 5°20'00"W; 29 May 1925; E. Hartert leg.; NHMUK (studied).

##### Other material.

Morocco • 1 ♂; Azrou; 30°40'00"N, 7°30'00"W; 31 May 1953; G. L. Spoek leg.; NBCN • 2 ♂♂; Moyen Atlas, Azrou; 30°40'00"N, 7°30'00"W; 19 Jun. 1928; R. Benoist leg.; MNHN PM0344, PM0350 • 1 ♀; Moyen Atlas, Azrou; 30°40'00"N, 7°30'00"W; 16 Jun. 1928; R. Benoist leg.; MNHN PM0371 • 1 ♀; Middle Atlas, Azrou; 33°24'51"N, 5°11'36"W; 1789 m a.s.l.; 25–26 Jun. 2014; A. Vujić, S. Radenković, J. Ačanski, S. Veselić leg.; FSUNS 07044 • 1 ♂; Moyen Atlas, Azrou; 33°25'48"N, 5°12'36"W; 16 Jun. 1928, R. Benoist leg.; MNHN 22624 • 1 ♂; Middle Atlas, Maknes, Azrou; 33°25'48"N 5°12'36"W; 1800 m a.s.l.; 25 May 1995; C. Kassebeer leg.; FSUNS 02496.

#### Merodon trianguloculus

Taxon classificationAnimaliaDipteraSyrphidae

Vujić, Likov & Radenković sp. nov.

F928E918-8021-50FF-AEEB-8775298BCCD1

http://zoobank.org/343FE864-04B3-4B5A-BC75-217DDEFA7CDE

Figs 23A–D , 33A–C , 34A–C

##### Diagnosis.

Medium sized (7.5–11.6 mm), dark brown species with characteristic large silver microtrichose fasciae on terga 2–4 in males (Fig. 23C), and silver michrotrichose ornamentation on scutum in both sexes (Fig. 23A, B); basoflagellomere with rounded apex, 1.6–1.8 times longer than wide in male (Fig. 33A, B).

##### Description.

**Male.** Head. Antennae black to dark brown; basoflagellomere rounded, 1.6–1.8 times as long as wide, and ca. 2.3 times as long as pedicel; large fossette dorsomedial and dorsolateral; arista brown and thickened at basal one third, covered with dense microtrichia, ca. 1.8 times as long as basoflagellomere (Fig. 33A, B); face and frons black with gray microtrichia, face covered with dense whitish gray, and frons with yellowish gray pile; oral margin shiny with microtrichose lateral areas; lunule shiny black, bare; vertex covered with gray microtrichia; vertex isosceles, with long, pale whitish yellow pile mixed with black pile on the ocellar triangle; ocellar triangle equilateral; eyes covered with dense pile; occiput with gray-yellow pile, covered with a dense, gray microtrichia; eye contiguity 8–12 facets long.

Thorax. Scutum and scutellum black with bronze luster, covered with dense, erect, yellow pile; scutum with conspicuous silver microtrichose ornamentation (Fig. 23A); posterodorsal part of anterior anepisternum, posterior anepisternum (except anteroventral angle), anterior anepimeron, dorsomedial anepimeron, and posterodorsal and anteroventral parts of katepisternum with long, pale yellow pile and grayish microtrichia; wings entirely covered with microtrichia; wing veins brown; calypteres and halteres pale yellow; legs mostly black, except yellowish tip of femora, basal and apical part of tibiae and brown tarsi ventrally; pile on legs pale yellow; metafemur moderately incrassate, ca. four times longer than wide; pile on metafemur long, and ca. half to two thirds of width of metafemur.

Abdomen. Tapering, 1.2 times longer than mesonotum; terga dark, with broad silver microtrichose fasciae; tergum 2 with pale orange lateral maculae; pile on terga all yellow (Fig. 23C); sterna dark brown, covered with long whitish yellow pile.

Male genitalia. Apical part of anterior surstyle lobe rhomboid shape, approximately as long as wide, covered with dense, short pile (Fig. 34A: al); posterior surstyle lobe oval with basolateral protrusion (lateral hump) (Fig. 34A, B: bp); hypandrium sickle-shaped, without lateral projections; lingula (Fig. 34C: l).

**Female.** Similar to the male except for normal sexual dimorphism and for the following characteristics: basoflagellomere ca. 1.8 times longer than wide, fossette dorsolateral (Fig. 33C); frons with microtrichose vittae along eye margins; frons covered with mostly gray-yellow pile mixed with black ones; ocellar triangle covered with black pile; ventral margin of metafemur with sparse pilosity, only individual pile longer; lateral side of terga, anterior two third of tergum 2 and all tergum 5 with whitish pile; terga 2–4 with short adpressed black pile medially; microtrichose fasciae on terga 3 and 4 narrower (Fig. 23D).

##### Etymology.

The name *trianguloculus* derives from the Latin adjective *triangulus* (triangular) and Latin noun *loculus* (spot) and describes the distinctive triangular silver pollinose fasciae on the abdomen.

##### Distribution.

*Merodon trianguloculus* sp. nov. was recorded only in Turkmenistan (Fig. 7).

##### Ecology.

Preferred environment: open areas extending to the forest zone; unimproved grassland; adults resting on the stones and in flight between grasses at the top of Dushak Mountain. Flowers visited: no data. Flight period: May-June.

##### Type material.

**Holotype**. Turkmenistan • ♂; 120 km SW Geok-Tepe town; 38°10'31"N, 57°58'01"E; 11 May 1988; A. Barkalov leg.; SZMN 05818. Original label: “HOLOTYPE of *Merodon* / *trianguloculus* Vujić, Likov / et Radenković sp.n. 2019” [red label], “Туркмения, I20 км / юз Геок–Тепе 11.У. I988 / Сб.А. Баркалов”, “05818” (See Supplementary file 5: Figure 5). **Paratypes.** Turkmenistan • 1 ♀; 15 km k-s pos. Firjuza settlement, Dushak Mountain; 18 May 1988; A. Barkalov leg.; SZMN 05819 • 1 ♀; same data as for preceding; SZMN 05837 • 1 ♀; Centr. Kopetdag g. Dušak; 2100–2300 m a.s.l.; 6 Jun. 1986; Dubatolov leg.; SZMN 05844 • 1 ♂; Firjuza settlement 15 km zap., Dushak Mountain; 16 May 1988; A. Barkalov leg.; SZMN 05816 • 1 ♂; same data as for preceding; 8 May 1987; SZMN 05840 • 1 ♂; 120 km SW Geok-Tepe town; 38°10'31"N, 57°58'01"E; 11 May 1988; A. Barkalov leg.; SZMN 05817.

#### Merodon trizonus

Taxon classificationAnimaliaDipteraSyrphidae

(Szilády, 1940) nomen dubium

3EF7414E-A1FA-5C65-961F-5D765282F57D

##### Remarks.

The identity of *Merodon trizonus* remains unclear. The species was described based on two male and two female syntypes labelled “La Calle [el Kala], Algeria” and “Ain Draham, Tunisia”, which were not examined. Originally, the syntypes were located in the Hungarian National Museum in Budapest, but the Diptera collection was destroyed by a fire in 1956. The description of Szilády (1940) is incomplete and based on a few differences from the related species *M. hirsutus*. The types of *M. trizonus* are assumed lost and the description is insufficiently accurate to associate name to one of these species. Currently, two species from the *M. serrulatus* species group, to which *M. hirsutus* belongs, occur in northern Africa, namely *M. bequaerti* and *M. sophron.* Therefore, we propose to leave the name *Merodon trizonus* (Szilády, 1940) as *nomen dubium*.

### Key to the *Merodon serrulatus* species group

**Table:** 

1	Posterior part of mid coxa without long pile (***Merodon avidus-nigritarsis* lineage**)	**2**
–	Posterior part of mid coxa with long pile	**other *Merodon* lineages** ^1^
2	Taxa with characteristic basolateral protrusion (lateral hump) on posterior surstyle lobe (as on Figs 1, 6: bp). Species with dark scutum and usually whitish microtrichose fasciae on terga 2–4, at least in females (as on Fig. 15C); tergum 2 usually with a pair of reddish orange lateral maculae; abdomen elongated, usually narrow and tapering, slightly longer than scutum and scutellum together (as on Figs 26, 29); legs mostly black; metafemur incrassate (as on Fig. 4); tarsi black dorsally and dark brown ventrally; antennae usually dark; basoflagellomere usually obviously concave dorsally (as on Figs 11, 12); male genitalia: apical part of anterior surstyle lobe more or less of rhomboid to triangular shape, covered with dense short pile (as on Fig. 1: al); cercus rectangular, without prominences (as on Fig. 14A: c); hypandrium elongated and sickle-shaped (as on Fig. 1); lateral sclerite of aedeagus finger-like with basal thorn-like process (Fig. 1G: s); lingula usually present (as on Fig. 1E: l) (***Merodon serrulatus* group**)	**3**
–	Species without basolateral protrusion (lateral hump) on posterior surstyle lobe in males and with different combinations of characters in females	**other species groups belonging to the *Merodon avidus-nigritarsis* lineage** ^2^
3	Males	**4**
–	Females	**19**
4	Eyes dichoptic (as on Figs 16B, 27A)	**5**
–	Eyes holoptic (as on Fig. 5B)	**6**
5	Black species with predominantly black body pile, especially on thorax (Fig. 26A, B); terga 2–4 bare or with indistinct microtrichose fasciae (Fig. 26A); distribution: Tajikistan (Fig. 7)	***Merodon nigrocapillatus* sp. nov.**
–	Species with olive-brown reflection, predominantly covered with pale yellow pile; terga 2–4 with conspicuous lateral microtrichose fasciae (Fig. 15A); distribution: Kyrgyzstan and Kazakhstan (Fig. 7)	***Merodon disjunctus* sp. nov.**
6	Bluish species (Fig. 29A) with dark macula on the medial part of the wing (Fig. 29C) and whitish pale body pile; distribution: Uzbekistan (Fig. 7)	***Merodon nigropunctum* sp. nov.**
–	Dark brown species without dark macula on wings	**7**
7	Tergum 2 entirely dark brown to black (as on Fig. 21A)	**8**
–	Tergum 2 with yellow-orange lateral maculae (at least small ones) (as on Fig. 23E)	**10**
8	Scutum without black pile, except few black setae at wing basis in some specimens; terga 2–4 with a conspicuous microtrichose fasciae (as on Fig. 21A)	**9**
–	Scutum with black pile, at least on fascia between wing basis; terga 2–4 with a less conspicuous microtrichose fasciae (as on Fig. 2A)	***Merodon serrulatus* (Wiedemann in Meigen, 1822) (part)**
9	Dorsolateral pile on metafemur dense and longer (Fig. 13C); terga with longer and erect pile (Fig. 21F); tergum 2 shiny; posterior surstyle lobe with big lateral hump, clearly visible in ventral view (Fig. 14E, F: bp); distribution: Syria, Israel and south-eastern Turkey (Fig. 7)	***Merodon hirsutus* Sack, 1913**
–	Dorsolateral pile on metafemur shorter (Fig. 13A); terga with shorter pile, adpressed at tergum 4 (Fig. 21C); tergum 2 dull; posterior surstyle lobe with small lateral hump, less distinct in ventral view (Fig. 14H, I: bp); distribution: Lesvos Island (Greece) and western Turkey (Fig. 7)	***Merodon opacus* sp. nov.**
10	Terga 3 and 4 with a pair of broad silver microtrichose maculae (Fig. 23C); scutum with characteristic silver microtrichose ornamentation (Fig. 23A); distribution: Turkmenistan (Fig. 7)	***Merodon trianguloculus* sp. nov.**
–	Terga 3 and 4 without or with a less conspicuous pair of broad silver microtrichose fasciae (as on Fig. 10)	**11**
11	Terga 3 and 4 without microtrichose fasciae	**12**
–	Terga 3 and 4 with a pair of white microtrichose, oblique fasciae (as on Fig. 23E)	**13**
12	Metafemur strongly curved (Fig. 8C); body pile longer, clearly visible on tergum 4 (Fig. 10D); distribution: Iberian Peninsula (Fig. 7)	***Merodon sacki* (Paramonov, 1936)**
–	Metafemur less curved (Fig. 8A); body pile shorter, clearly visible on tergum 4 (Fig. 10B); distribution: north-west Africa (Fig. 7)	***Merodon bequaerti* Hurkmans, 1993 (part)**
13	Antennae reddish yellow; basoflagellomere short and broad, ca. 1.2 times as long as wide, with large dorsal to dorsolateral fossette (Fig. 19E–G); tergum 2 with large reddish yellow lateral maculae (Fig. 23E); tergum 3 laterally reddish or brown; tibiae and tarsi partly reddish brown; metafemur incrassate with long pilosity as long as half of width of metafemur (Fig. 22A); distribution: Japan and China (Fig. 7)	***Merodon kawamurae* Matsumura, 1916**
–	Antennae dark brown/black; basoflagellomere elongated; legs mostly black	**14**
14	Abdomen broad, tergum 2 at least 2.5 times wider than long (as on Fig. 24A); metafemur incrassate and curved (as on Fig. 8C)	**15**
–	Abdomen narrower, tergum 2 ca. two times wider than long (as on Fig. 2); metafemur less incrassate and with almost straight lateral margin (as on Fig. 4)	**16**
15	Pile on ventral margin of metafemur very short (Fig. 22C); distribution: Crete Island (Greece) (Fig. 7)	***Merodon medium* sp. nov.**
–	Pile on ventral margin of metafemur long and dense (Fig. 8A); distribution: north-west Africa (Fig. 7)	***Merodon bequaerti* Hurkmans, 1993 (part)**
16	Male genitalia: basolateral protrusion (lateral hump) on posterior surstyle lobe reduced (Fig. 14A: bp); distribution: western Turkey (Fig. 7)	***Merodon defectus* sp. nov.**
–	Male genitalia: basolateral protrusion (lateral hump) on posterior surstyle lobe well developed (as on Fig. 1, 6: bp), visible at least from ventral view (Fig. 14C: bp)	**17**
17	Medial fossette absent (as on Fig. 11H)	**18**
–	Medial fossette present (Fig. 3B)	***Merodon serrulatus* (Wiedemann in Meigen, 1822)^3^**
18	GenBank acc. no. MN623564-MN623581; distribution: Palaearctic, extending from France in the west to Turkey in the south-east, and to Siberia toward north-east (Fig. 7)	***Merodon serrulatus* (Wiedemann in Meigen, 1822) (part)**
–	GenBank acc. no. MN623540; distribution: north-west Africa (Fig. 7)	***Merodon sophron* Hurkmans, 1993**
19	Tergum 2 dark (as on Fig. 26C, D)	**20** ^4^
–	Tergum 2 with reddish lateral maculae (as on Fig. 23F)	**22**
20	Black species (Fig. 26C, D); body covered with predominantly black pilosity, especially on thorax; distribution: Tajikistan (Fig. 7)	***Merodon nigrocapillatus* sp. nov.**
–	Species with olive-brown reflection, predominantly covered with pale pile	**21**
21	Pile on dorsolateral margin of metafemur long (Fig. 13D); distribution: Syria and south-east Turkey (Fig. 7)	***Merodon hirsutus* Sack, 1913**
–	Pile on dorsolateral margin of metafemur short (Fig. 13B); distribution: Lesvos Island (Greece) and western Turkey (Fig. 7)	***Merodon opacus* sp. nov.**
22	Metafemur with long pile on the entire surface of ventral margin (as on 22B) (unknown female of *Merodon sacki* (Paramonov, 1936), probably keys out here)	**23**
–	Metafemur with mostly short pile on ventral margin (as on Fig. 4C)	**27**
23	Scutum with characteristic silver microtrichose ornamentation (Fig. 23B); terga 3 and 4 with broad lateral microtrichose fasciae; distribution: Turkmenistan (Fig. 7)	***Merodon trianguloculus* sp. nov.**
–	Scutum with indistinct microtrichose vittae; terga 3 and 4 with narrower lateral microtrichose fasciae	**24**
24	Scutum at wing basis with only yellowish pilosity; metafemur with long, dense dorsal pile (as on Fig. 22B)	**25**
–	Scutum at wing basis with short black pile; metafemur with sparse dorsal pile (as on Fig. 8B)	**26**
25	Blackish species; terga 3 and 4 with broad lateral microtrichose fasciae; basoflagellomere dark brown to black; distribution: Kyrgyzstan and Kazakhstan (Fig. 7)	***Merodon disjunctus* sp. nov.**
–	Brownish species; terga 3 and 4 with narrower lateral microtrichose fasciae (Fig. 23F); basoflagellomere yellowish; distribution: Japan and China (Fig. 7)	***Merodon kawamurae* Matsumura, 1916**
26	Distribution: north-west Africa (Fig. 7)	***Merodon bequaerti* Hurkmans, 1993**
–	Distribution: France	***Merodon serrulatus* (Wiedemann in Meigen, 1822) (part)**
27	Females of these three species can be separated by distribution and genetic data:
–	GenBank acc. no. MN623540. Distribution: North-west Africa (Fig. 7)	***Merodon sophron* Hurkmans, 1993**
–	GenBank acc. no. MN623564-MN623581. Distribution: Palaearctic, extending from Iberian Peninsula in the west to Turkey in the south-east and toward Siberia in the north-east (Fig. 7)	***Merodon serrulatus* (Wiedemann in Meigen, 1822) (part)**
–	Distribution: western Turkey (Fig. 7)	***Merodon defectus* sp. nov.**

### Molecular inference

The final aligned and pruned dataset including two-gene data matrix (COI+28S rRNA) comprised 1,859 nucleotide characters (421 parsimony informative sites) pertaining to 81 specimens (79 in-group specimens of the studied genus *Merodon* lineages along with two outgroups). The final number of aligned sites for COI gene (concatenated 3’ and 5’ fragments of the gene) included 1,273 nucleotides, while 586 nucleotide characters (with gaps) were included in analyses for the D2−3 region of the 28S rRNA gene.

**Figure 36.**
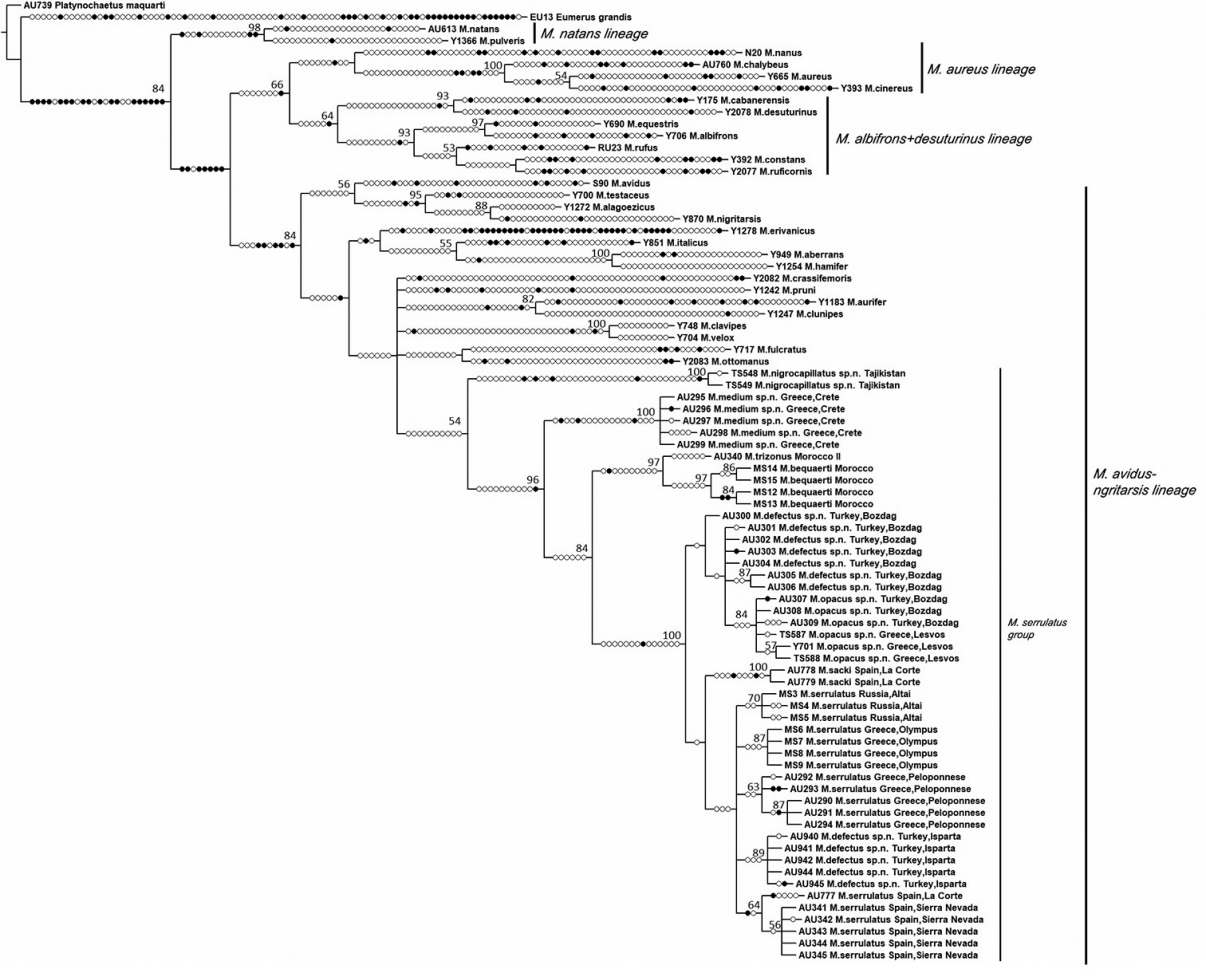
Strict consensus tree of 41 most parsimonious trees from the analysis of combined COI mitochondrial and 28S nuclear genes sequences. Length 2093 steps, Consistency Index (CI) 37, Retention Index (RI) 65. Bootstrap support values are depicted near nodes (≥ 50). Filled circles represent non-homoplasious changes and open circles are homoplasious changes. Four lineages observed in the genus *Merodon*, as well as the *M. serrulatus* group, are marked on the tree.

Both obtained phylogenetic trees (Maximum Parsimony, Fig. 36 and Maximum Likelihood, Supplementary file 7: Figure 7) resolved the four previously described lineages as clades, while the *M. serrulatus* species group was recovered as monophyletic within the *Merodon avidus-nigritarsis* lineage (MP = 54, ML = 75). Within the *serrulatus* species group, specimens belonging to *M. nigrocapillatus* sp. nov., *M. medium* sp. nov., *M. bequaerti* and *M. sacki* were clearly grouped together with high bootstrap support (MP = 100, ML = 100; MP = 100, ML = 100; MP = 97, ML = 94; MP = 100, ML = 100, respectively). The single sequenced specimen of *M. sophron* was resolved as sister taxon of *M. bequaerti*. Unfortunately, although morphologically differentiated, specimens identified as *M. defectus* sp. nov. clustered with *M. serrulatus* in a clade without support, but also with *M. opacus* sp. nov. in another clade without support. These three species together with *M. sacki* were resolved in a group with high support value. High level of inter-population molecular variability within *M. serrulatus* species was also detected.

## Discussion

### Taxon delimitation and integrative taxonomy

Likov et al. (2019) reported the monophyly of the *Merodon avidus-nigritarsis* lineage. Within this lineage, the *Merodon serrulatus* species group is supported in our phylogenetic analyses.

The *M. serrulatus* species group comprises six already described species (*Merodon bequaerti*, *M. hirsutus*, *M. kawamurae*, *M. sacki*, *M. serrulatus*, and *M. sophron*) and seven new species described here. Based on the present results, six species of this group, namely *M. disjunctus* sp. nov., *M. kawamurae*, *M. medium* sp. nov., *M. nigrocapillatus* sp. nov., *M. nigropunctum* sp. nov., and *M. trianguloculus* sp. nov., are delimited on differences of morphological characters. Moreover, two pairs of very similar species can be separated from other species of the group by some distinct characters, but the distinction between the species in each pair is based on characters with more subtle differences. These two pairs are *M. bequaerti* / *M. sacki*, with the metafemur incrassate and long pile on postero- and anteroventral surface of the metafemur, and *M. hirsutus* / *M. opacus* sp. nov., with tergum 2 dark, without yellow-orange lateral maculae in both sexes.

The remaining three species within the *M. serrulatus* species group are morphologically very similar to each other. *Merodon defectus* sp. nov. has subtle, but stable differences in structures of the male genitalia serving as diagnostic characters (lateral hump on posterior surstyle lobe reduced). Closely related and very similar, *M. serrulatus* and *M. sophron* are distinguished by molecular data, in addition to a clear morphological diagnostic character in males (presence or absence of medial antennal fossette).

Using different methodologies to assess various aspects of the diversity of the genus *Merodon*, previous authors (Mengual et al. 2006; Marcos-García et al. 2007, 2011; Ståhls et al. 2009; Radenković et al. 2011; Vujić et al. 2012, 2013, 2015) have shown the potential of the integrative taxonomy to indicate cryptic taxa, to define new species and to point out different evolutionary lineages. The integration of multiple data sources, combining different molecular (Popović et al. 2014, 2015; Ačanski et al. 2016; Šašić et al. 2016; Kočiš Tubić et al. 2018; Radenković et al. 2018a), morphological (Popović et al. 2015), distributional (Radenković et al. 2018a), and environmental (Popović et al. 2015; Vujić et al. 2015; Šašić et al. 2016) information, has proven to be significant in re-evaluating taxonomic delimitations within the *Merodon* genus. Although results of this integrative approach have not been always congruent (Mengual et al. 2006; Ståhls et al. 2009; Popović et al. 2015; Radenković et al. 2018a).

In the present study we applied this integrative approach, i.e., to combine morphology, genetic data, and distribution, to support the taxonomic status and systematic decisions made for the *M. serrulatus* species group. For example, the species *M. sophron* and *M. serrulatus*, although morphologically similar, are conspicuously separated from each other based on molecular data. The same situation is found between *M. bequaerti* and *M. sacki*. In contrast, the morphologically distinct species *M. defectus* sp. nov., *M. serrulatus*, and *M. opacus* sp. nov. cluster together in the molecular analysis. Discordance between morphological and molecular data has been observed in some previous studies concerning closely related taxa within the family Syrphidae (e.g., Ståhls et al. 2009; Francuski et al. 2014; Haarto and Ståhls 2014), as well as in recently conducted studies on *Merodon* species groups (Likov et al. 2019). In the present study the molecular data for *M. defectus* sp. nov. show some interpopulation differentiation: while the specimens from Bozdag (Turkey) were resolved in the same cluster with *M. opacus* sp. nov., the specimens of *M. defectus* sp. nov. from Isparta (Turkey) and *M. serrulatus* were resolved in another cluster. Unfortunately, these two clades do not have support and the whole cluster, including *M. sacki*, could be resolved in a large polytomy when collapsing nodes without high support. Different molecular profile of different populations of one species was also detected by Likov et al. (2019). The suggested reasons for the low COI divergence between these species are retained polymorphism or mitochondrial introgression between the taxa, as it has been hypothesized in previous studies (e.g., Ståhls et al. 2009; Francuski et al. 2014; Haarto and Ståhls 2014).

It is important to do further taxonomic research with the populations of *Merodon serrulatus* with high inter-population morphological and genetic variability. These populations may be also geographically isolated and are posited to exhibit low genetic flow. The very wide distributional range of *M. serrulatus*, extending from Iberian Peninsula to Mongolia, is highly unusual in the genus *Merodon*, thus exemplifying a complex population structure that might contain evolutionary units at different levels of speciation.

### Immature stages

One of the main reasons for the gap in extant knowledge on the immature stages of *Merodon* species is the difficulty of finding specimens in the field, since host plants, the larval food-plants and the breeding and oviposition sites, have not been recorded for the great majority of *Merodon* species (Hurkmans 1993; Rotheray 1993; Speight 2018). The description of the puparium of *Merodon opacus* sp. nov. in this work is based on a single specimen reared from the larva found in the soil near the bulbs of *Fritillaria*, *Gagea*, *Muscari*, and *Ornithogalum*. In extant studies, the immature stages of *Merodon* species were linked to bulbous geophytes, mostly belonging to plant families Asparagaceae (Ricarte et al. 2008; Andrić et al. 2014; Preradović et al. 2018), Iridaceae (Stuckenberg 1956) and Amaryllidaceae (Heiss 1938; Ricarte et al. 2017).

The morphology of the puparium of *M. opacus* sp. nov. shows similarities with the puparium of *M. avidus* in terms of the morphology of the posterior respiratory process (prp) and ornamentation of pupal spiracles (Preradović et al. 2018). In fact, these species share the button-shaped prp and the poorly defined outline of the spiracular plate, whereas the spiracular openings of *M. opacus* sp. nov. are less convoluted than those in *M. avidus*. The pupal spiracles are stout in shape (almost as long as broad) and are clearly shorter than in *M. avidus*, but share the reticulated ornamentation (polygonal pattern).

A single larva of *Merodon opacus* sp. nov. was found in the ground surrounded with bulbs of different plant genera (*Fritillaria*, *Gagea*, *Muscari*, *Ornithogalum*). Recent larval records suggest that groups of related *Merodon* species could have the same plant genus as a host. These close relationships could be suspected between: *M. constans* species group and *Galanthus* L. (Amaryllidaceae) [Popov and Mishustin (pers. comm) confirmed that eight species of the *constans* species group feed on bulbs of eleven snowdrop species], *M. aureus* species group and *Crocus* L. (Iridaceae) [Speight 2018; Popov pers. comm.], and *M. geniculatus* species group and *Narcissus* L. (Amaryllidaceae) [i.e., *M. eques* (Fabricius, 1805) (see Pehlivan and Akbulut 1991), *M. geniculatus* Strobl, 1909 (see Ricarte et al. 2017), and *M. neofasciatus* Ståhls & Vujić, 2018 (see Vujić et al. 2018)]. Based on these findings, we suggest that the host plant for the members of the *M. serrulatus* species group should be a plant genus present on its large range, extending from North Africa, throughout the entire Palaearctic region to Japan. Two bulb genera with native ranges (WCSP 2019) fitting this distribution, *Gagea* and *Fritillaria* (Liliaceae), might be the larval food-plants. Future research in this field could thus focus on more detailed field work in areas characterized by numerous populations of species from the *M. serrulatus* species group.

### Distribution and species diversity

Being distributed from the Iberian Peninsula in the south-west, along the Mediterranean and Balkan Peninsula, through Turkey and southern Russia to Siberia and Mongolia in the north-east, *Merodon serrulatus* is the species of the genus *Merodon* with the largest distributional range. Other species of the *M. serrulatus* species group can be found at the edges of this distributional range, albeit with a much more restricted distribution. For example, *M. sacki* has been found in southern Spain, *M. medium* sp. nov. is endemic to Crete Island, whereas *M. defectus* sp. nov. and *M. opacus* sp. nov. have been recorded in western Turkey, with the latter species also being found on Lesvos Island, and *M. hirsutus* found in south-eastern Turkey, Israel and Syria. The *M. serrulatus* species group includes two North-African species, i.e., *M. sophron* restricted to Morocco, and *M. bequaerti* more widely distributed along the Mediterranean coast of the African continent. Only one species of the group, *M. kawamurae*, is found in the Far East of the Palearctic region, i.e., in central and south-eastern China and Japan. It is worth noting that four of the seven newly described species are distributed in Central Asia, the central and somewhat isolated part of the distribution range of the *M. serrulatus* species group. *Merodon disjunctus* sp. nov. is found in Kyrgyzstan and Kazakhstan, *M. nigrocapillatus* sp. nov. has been collected in Tajikistan, whereas *M. nigropunctum* sp. nov. and *M. trianguloculus* sp. nov. are found in Uzbekistan and Turkmenistan, respectively.

The genus *Merodon* is known to be widespread in regions such as the Mediterranean Basin, with high diversity of geophytes, whereby underground storage organs serve as larval food sources for *Merodon* species (Ricarte et al. 2008, 2017). Such potential for the development of a high diversity of *Merodon* taxa might explain their current geographical distributions (Vujić et al. 2011, 2013). The highest number of *Merodon* species and the greatest endemicity level in the Mediterranean Basin was noted for the Anatolian region (Vujić et al. 2011), which represents the main center of *Merodon* diversity within the Palaearctic region, along with the Iberian Peninsula (Marcos-García et al. 2007). The high number of endemic species in the eastern Mediterranean Basin has been suggested to be related to the intense orogenic activity favoring isolation and allopatric speciation (Vujić et al. 2011). The biologically diverse Anatolian region, characterized by a rich geological history, comprises of an extensive system of high mountain chains and closed basins, thus providing a wide range of habitats. Throughout history, different parts of this topographically complex area, connecting diverse geographic regions of Asia and Europe, have served not only as natural barriers but also as highly important refugia and corridors providing passages for species spreading (Vujić et al. 2013, 2015).

Central Asia is characterized by many mountains exceeding 6,500 m in elevation, as well as by major desert basins, which have thus far remained understudied. This is particularly the case for the alpine areas, and especially in terms of the invertebrate fauna (CEPF 2017). The very diverse flora of this region harbors a large number of endemics, including many bulbous plants (CEPF 2017) which can support high diversity of *Merodon* taxa, including the four endemic species of the *M. serrulatus* species group described here. Major mountain ranges located in Central Asia represent an extensive zone for faunistic evolution and differentiation, not only ecologically, but also orographically and biogeographically (Mani 1968). Heterogeneous topography with various isolated habitats along altitudinal gradients fosters high rates of speciation, species diversity and endemism. Climatic fluctuations and tectonic processes throughout the complex geological history of this region have contributed to its unique climate and have promoted high levels of floristic diversification and alpine endemism, while also affecting the distributions and structure of many taxa (e.g., Djamali et al. 2012; Zinenko et al. 2015). Having a long history as the crossroads between east and west, this region has historically been subjected to high levels of anthropogenic disturbance that continue to the present day, and populations of many species have declined due to habitat modifications (Djamali et al. 2012; CEPF 2017). The results yielded by the present study confirm previous conclusions emphasizing the importance of such underexplored regions as centers of endemicity, hosting habitats potentially harboring hidden diversity within the genus *Merodon* (Vujić et al. 2019).

## Supplementary Material

XML Treatment for Merodon bequaerti

XML Treatment for Merodon defectus

XML Treatment for Merodon disjunctus

XML Treatment for Merodon hirsutus

XML Treatment for Merodon kawamurae

XML Treatment for Merodon medium

XML Treatment for Merodon nigrocapillatus

XML Treatment for Merodon nigropunctum

XML Treatment for Merodon opacus

XML Treatment for Merodon sacki

XML Treatment for Merodon serrulatus

XML Treatment for Merodon sophron

XML Treatment for Merodon trianguloculus

XML Treatment for Merodon trizonus

## Footnotes

not treated here

not treated here

part: Iberian populations

unknown female of Merodon nigropunctum sp. nov. probably keys out here

## Supplementary materials

Supplementary material 1 Figure S1 Data type: type specimens' data Explanation note: **A***Merodon bequaerti*, holotype and labels **B***Merodon nigrocapillatus* sp. nov., holotype and labels. https://binary.pensoft.net/file/377398This dataset is made available under the Open Database License (http://opendatacommons.org/licenses/odbl/1.0/). The Open Database License (ODbL) is a license agreement intended to allow users to freely share, modify, and use this Dataset while maintaining this same freedom for others, provided that the original source and author(s) are credited.Ante Vujić, Laura Likov, Snežana Radenković, Nataša Kočiš Tubić, Mihajla Djan, Anja Šebić, Celeste Pérez-Bañón, Anatolij Barkalov, Rüstem Hayat, Santos Rojo, Andrijana Andrić, Gunilla Ståhls

Supplementary material 2 Figure S2 Data type: type specimens' data Explanation note: **A***Merodon defectus* sp. nov., holotype and labels **B***Merodon disjunctus* sp. nov., holotype and labels. https://binary.pensoft.net/file/377399This dataset is made available under the Open Database License (http://opendatacommons.org/licenses/odbl/1.0/). The Open Database License (ODbL) is a license agreement intended to allow users to freely share, modify, and use this Dataset while maintaining this same freedom for others, provided that the original source and author(s) are credited.Ante Vujić, Laura Likov, Snežana Radenković, Nataša Kočiš Tubić, Mihajla Djan, Anja Šebić, Celeste Pérez-Bañón, Anatolij Barkalov, Rüstem Hayat, Santos Rojo, Andrijana Andrić, Gunilla Ståhls

Supplementary material 3 Figure S3. *Merodon medium* sp. nov., holotype and labels Data type: type specimens' data Explanation note: *Merodon medium* sp. nov., holotype and labels. https://binary.pensoft.net/file/377400This dataset is made available under the Open Database License (http://opendatacommons.org/licenses/odbl/1.0/). The Open Database License (ODbL) is a license agreement intended to allow users to freely share, modify, and use this Dataset while maintaining this same freedom for others, provided that the original source and author(s) are credited.Ante Vujić, Laura Likov, Snežana Radenković, Nataša Kočiš Tubić, Mihajla Djan, Anja Šebić, Celeste Pérez-Bañón, Anatolij Barkalov, Rüstem Hayat, Santos Rojo, Andrijana Andrić, Gunilla Ståhls

Supplementary material 4 Figure S4 Data type: type specimens' data Explanation note: **A***Merodon nigropunctum* sp. nov., holotype and labels **B***Merodon opacus* sp. nov., holotype and labels https://binary.pensoft.net/file/377401This dataset is made available under the Open Database License (http://opendatacommons.org/licenses/odbl/1.0/). The Open Database License (ODbL) is a license agreement intended to allow users to freely share, modify, and use this Dataset while maintaining this same freedom for others, provided that the original source and author(s) are credited.Ante Vujić, Laura Likov, Snežana Radenković, Nataša Kočiš Tubić, Mihajla Djan, Anja Šebić, Celeste Pérez-Bañón, Anatolij Barkalov, Rüstem Hayat, Santos Rojo, Andrijana Andrić, Gunilla Ståhls

Supplementary material 5 Figure S5. *Merodon trianguloculus* sp. nov., holotype and labels Data type: type specimens' data https://binary.pensoft.net/file/377402This dataset is made available under the Open Database License (http://opendatacommons.org/licenses/odbl/1.0/). The Open Database License (ODbL) is a license agreement intended to allow users to freely share, modify, and use this Dataset while maintaining this same freedom for others, provided that the original source and author(s) are credited.Ante Vujić, Laura Likov, Snežana Radenković, Nataša Kočiš Tubić, Mihajla Djan, Anja Šebić, Celeste Pérez-Bañón, Anatolij Barkalov, Rüstem Hayat, Santos Rojo, Andrijana Andrić, Gunilla Ståhls

Supplementary material 6 Figure S6 Data type: type specimens' data Explanation note: **A***Merodon tener*, lectotype and labels **B***Merodon sacki*, holotype and labels. https://binary.pensoft.net/file/377403This dataset is made available under the Open Database License (http://opendatacommons.org/licenses/odbl/1.0/). The Open Database License (ODbL) is a license agreement intended to allow users to freely share, modify, and use this Dataset while maintaining this same freedom for others, provided that the original source and author(s) are credited.Ante Vujić, Laura Likov, Snežana Radenković, Nataša Kočiš Tubić, Mihajla Djan, Anja Šebić, Celeste Pérez-Bañón, Anatolij Barkalov, Rüstem Hayat, Santos Rojo, Andrijana Andrić, Gunilla Ståhls

Supplementary material 7 Figure S7 Data type: phylogenetic data Explanation note: Maximum likelihood tree based on analysis of combined COI mitochondrial and 28S nuclear genes sequences. Bootstrap support values of the main clades of the analysed *Merodon serrulatus* group of species are depicted near nodes (≥ 50). https://binary.pensoft.net/file/377404This dataset is made available under the Open Database License (http://opendatacommons.org/licenses/odbl/1.0/). The Open Database License (ODbL) is a license agreement intended to allow users to freely share, modify, and use this Dataset while maintaining this same freedom for others, provided that the original source and author(s) are credited.Ante Vujić, Laura Likov, Snežana Radenković, Nataša Kočiš Tubić, Mihajla Djan, Anja Šebić, Celeste Pérez-Bañón, Anatolij Barkalov, Rüstem Hayat, Santos Rojo, Andrijana Andrić, Gunilla Ståhls

Supplementary material 8 Table S1. Data for the specimens used in the molecular analysis, including GenBank accession numbers Data type: molecular specimens' dataset https://binary.pensoft.net/file/377405This dataset is made available under the Open Database License (http://opendatacommons.org/licenses/odbl/1.0/). The Open Database License (ODbL) is a license agreement intended to allow users to freely share, modify, and use this Dataset while maintaining this same freedom for others, provided that the original source and author(s) are credited.Ante Vujić, Laura Likov, Snežana Radenković, Nataša Kočiš Tubić, Mihajla Djan, Anja Šebić, Celeste Pérez-Bañón, Anatolij Barkalov, Rüstem Hayat, Santos Rojo, Andrijana Andrić, Gunilla Ståhls
