# Supplementary material for: Revision of the Merodon serrulatus group (Diptera, Syrphidae)
Source: Zookeys. 2020 Feb 5;909:79–158. doi: 10.3897/zookeys.909.46838 (PMC7015954; doi:10.3897/zookeys.909.46838)
Supplement: Supplementary material 1 [file zookeys-909-079-s008.pdf]

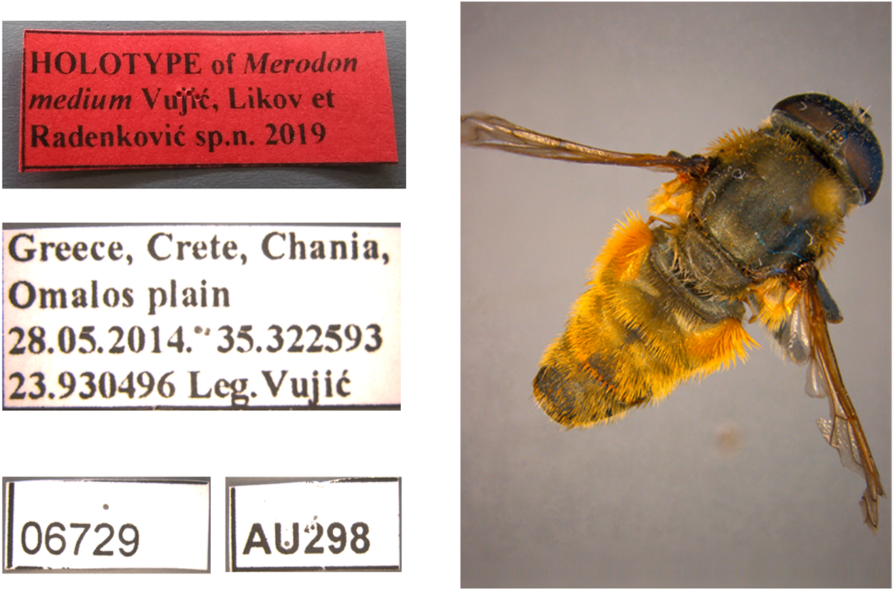
HOLOTYPE of Merodon
medium Vujić, Likov et
Radenković sp.n. 2019
Greece, Crete, Chania,
Omalos plain
28.05.2014. 35.322593
23.930496 Leg. Vujić
06729
AU298
